# *APOE* alleles are associated with sex-specific structural differences in brain regions affected in Alzheimer’s disease and related dementia

**DOI:** 10.1371/journal.pbio.3001863

**Published:** 2022-12-13

**Authors:** Chloé Savignac, Sylvia Villeneuve, AmanPreet Badhwar, Karin Saltoun, Kimia Shafighi, Chris Zajner, Vaibhav Sharma, Sarah A. Gagliano Taliun, Sali Farhan, Judes Poirier, Danilo Bzdok

**Affiliations:** 1 Department of Biomedical Engineering, Faculty of Medicine, McGill University, Montreal, Quebec, Canada; 2 Department of Neurology and Neurosurgery, Montreal Neurological Institute (MNI), Faculty of Medicine, McGill University, Montreal, Quebec, Canada; 3 McConnell Brain Imaging Centre (BIC), MNI, Faculty of Medicine, McGill University, Montreal, Quebec, Canada; 4 Department of Psychiatry, Faculty of Medicine, McGill University, Montreal, Quebec, Canada; 5 Centre for Studies in the Prevention of Alzheimer’s Disease, Douglas Mental Health Institute, McGill University, Montreal, Quebec, Canada; 6 Department of Pharmacology and Physiology, Faculty of Medicine, Université de Montréal, Montreal, Quebec, Canada; 7 Centre de recherche de l’Institut universitaire de gériatrie de Montréal (CRIUGM), Montreal, Quebec, Canada; 8 Department of Neurosciences & Department of Medicine, Faculty of Medicine, Université de Montréal, Montreal, Quebec, Canada; 9 Montreal Heart Institute, Montréal, Quebec, Canada; 10 Department of Human Genetics, Faculty of Medicine, McGill University, Montreal, Quebec, Canada; 11 School of Computer Science, McGill University, Montreal, Quebec, Canada; 12 Mila—Quebec Artificial Intelligence Institute, Montreal, Quebec, Canada; University of California Davis School of Medicine, UNITED STATES

## Abstract

Alzheimer’s disease is marked by intracellular tau aggregates in the medial temporal lobe (MTL) and extracellular amyloid aggregates in the default network (DN). Here, we examined codependent structural variations between the MTL’s most vulnerable structure, the hippocampus (HC), and the DN at subregion resolution in individuals with Alzheimer’s disease and related dementia (ADRD). By leveraging the power of the approximately 40,000 participants of the UK Biobank cohort, we assessed impacts from the protective *APOE* ɛ2 and the deleterious *APOE* ɛ4 Alzheimer’s disease alleles on these structural relationships. We demonstrate ɛ2 and ɛ4 genotype effects on the inter-individual expression of HC-DN co-variation structural patterns at the population level. Across these HC-DN signatures, recurrent deviations in the CA1, CA2/3, molecular layer, fornix’s fimbria, and their cortical partners related to ADRD risk. Analyses of the rich phenotypic profiles in the UK Biobank cohort further revealed male-specific HC-DN associations with air pollution and female-specific associations with cardiovascular traits. We also showed that *APOE* ɛ2/2 interacts preferentially with HC-DN co-variation patterns in estimating social lifestyle in males and physical activity in females. Our structural, genetic, and phenotypic analyses in this large epidemiological cohort reinvigorate the often-neglected interplay between *APOE* ɛ2 dosage and sex and link *APOE* alleles to inter-individual brain structural differences indicative of ADRD familial risk.

## Abstract 2

This study of the effects of APOE alleles ɛ2 and ɛ4 using structural brain scans from UK Biobank participants shows that the protective effect of the APOE ɛ2 allele on Alzheimer’s disease risk is sex-specific and linked to distinct hippocampus-default network co-variation regimes.

## Introduction

Around the globe, >50 million people are living with dementia—a global burden of >1 trillion USD$ annually [1]. By 2050, an estimated 3-fold increase in affected individuals is projected as a result of increased longevity [2]. The anticipated explosion in the number of dementia cases will put a strain on the 82 billion hours of annual informal care provided by caretakers worldwide [1]. In contrast to this secular trend, the age-specific prevalence of dementia is expected to decrease in certain high-income countries, which can be attributable to improvement in underlying health and socioeconomic determinants [2]. A recent authoritative report on dementia prevention has identified about a dozen potentially modifiable risk factors that could explain the disparity in Alzheimer’s disease and related dementia (ADRD) incidence [3]. The disparate risk dimensions include personal habits and lifestyle, physical and mental health, as well as societal and external factors. New public health policies targeted at reducing mid- to late-life risk factors (e.g., physical inactivity, social disengagement, loneliness) thus have the potential to delay dementia onset in the most disadvantaged older adults. As the global prevalence of dementia is quickly rising, there is an unprecedented need to characterise the impact of genetic predisposition (e.g., Apolipoprotein E (*APOE*) polymorphism [4]) and modifiable risk factors on ADRD-vulnerable brain structures before the onset of cognitive decline.

Over the past 2 decades, brain-imaging studies have converged on the disruption of a coherent network of higher association regions that involve key nodes of the default network (DN) in individuals with ADRD compared to healthy controls [5]. Extensive efforts have mobilised resting-state functional connectivity analyses to investigate patients with ADRD, with converging results in the DN [6]. However, delineating a definitive profile of functional connectivity deviations related to ADRD risk in healthy subjects was plagued with slow progress. Most such biomarker studies have attempted to identify functional connectivity patterns that reliably tell apart ɛ4 carriers from non-carriers. Yet, most other *APOE* variants have been largely neglected, perhaps because they occur much more infrequently in the general population. The extensive literature on altered DN connectivity in ɛ4 carriers has yet to reach consensus as reports of both increased [7] and decreased [8] connectivity within nodes of the DN have repeatedly led to contradictory conclusions. Among the few studies that could investigate concurrent connectivity alterations in the hippocampus (HC) and regions of the DN in ɛ4 carriers, the HC was typically treated as a monolithic structure [9] rather than appreciating its functional and structural heterogeneity. That is, it was studied as a single node when interrogating its coupling links to other DN nodes [10]. These inconsistencies are probably also due in part to data acquisition and preprocessing methods for functional connectivity analysis, which have made some findings in ɛ4 carriers hard to replicate [11]. Moreover, because of the overwhelming singular focus on ɛ4 carriers in the research community, the neural correlates associated with other *APOE* variants remain underspecified. Of particular appeal, illuminating the allegedly opposing effects of *APOE* ɛ2 and ɛ4 on DN and HC integrity could be crucial in guiding potential treatment avenues, given the ɛ2-associated protective outcome on brain structure [12].

A parallel stream of literature has focused on changes in hippocampal microstructure over the course of ADRD progression, mostly by performing thorough post-mortem autopsy on patients with probable ADRD. The HC formation is known for subfield-specific vulnerability to ADRD, at least since the late 1990s [13]. Yet, the HC is still routinely treated as if it was an anatomically homogeneous structure in common brain-imaging studies [9,14,15]. By extension, such an analytical approach is blind to the distinct links between HC subregions and DN subregions. In vivo examinations in the macaque monkey have shown that the HC formation receives important axon projections from the retrosplenial cortex and posterior cingulate cortex in the presubiculum and parasubiculum subregions [16]. Yet, the fornix, which carries the axons from the CA1 and subiculum subregions, forwards the only hippocampal output signals that directly go to the ventromedial and orbitofrontal cortex (OFC) of the DN [17,18]. Glossing over these known microanatomical nuances could explain reports of poor predictive value of hippocampal atrophy in early ADRD stages when measuring the whole HC as a single unit. In a randomised clinical trial, baseline HC volumes, manually traced and corrected for inhomogeneity, predicted conversion to ADRD over a 3-year period at 60.4% accuracy [19]. With the advent of ultra-high-resolution atlases and advanced automatic sub-segmentation techniques, assessment of the subfield-specific vulnerability of both hippocampi to ADRD progression in an observer-independent fashion is now coming into reach. Instead of relying primarily on post-mortem autopsy from patients to ultimately confirm ADRD status, we will soon be able to directly, non-invasively, quantify the level of risk of a given patient based on subfield-level granular information. From the perspective of clinical translation, coming up with individual profiles of microstructural alterations characteristic of ADRD risk could usher a principled path toward precision medicine in neurology.

For these reasons, here we opted for structural brain imaging to relate genetic risk to robust codependence principles between neocortical DN and allocortical HC at subregion granularity. Given the panoply of individual factors that may affect cortical blood flow (e.g., vigilance, mood, cortisol levels, and coffee intake), functional connectivity would likely paint a more circumstantial portrait of ADRD vulnerability. We therefore designed an analytical framework for doubly multivariate decomposition to zoom in on the structural correspondence between HC and DN subregions at the population level. The two-pronged approach was carefully tailored to derive coherent signatures of HC-DN co-variation sensitive to the subregion-specific vulnerability of these neural circuits in ADRD. We were able to quantify the level of risk by looking for structural deviation in individuals with and without family history of ADRD by deep inspection of concomitant regimes of HC-DN co-variation. Capitalising on the rich phenotyping available for 40,000 UK Biobank participants, our study could confront the effects of *APOE* ɛ2 and ɛ4 on inter-individual expressions of HC-DN co-variation—something out of reach in traditional brain-imaging studies involving small to medium sample sizes. In doing so, our study was also uniquely positioned to illuminate possible sex-specific associations across less prevalent *APOE* gene variants that previous brain-imaging investigations systematically ignored.

## Results

### Rationale

In post-mortem autopsy of patients with ADRD, structural alterations of microanatomically defined subregions composing the human HC have been described in extenso [20]. Despite such insights from rigorous invasive studies, the overwhelming majority of existing brain-imaging studies have treated the HC as a monolithic brain structure. Hence, the specific vulnerability of its heterogeneous subregions to ADRD pathology remains largely concealed today. Advances in automatic segmentation techniques for the HC using ex vivo brain imaging allow for subject-specific parcellations that respect the diversity of distinct subregions identified post-mortem. Capitalising on these ultra-high-resolution segmentations, we are now equipped to assess microstructural alterations of the human HC in a newly detailed way that scales to the approximately 40,000 UK Biobank (UKB) participants [21]. These advances enabled us to describe ADRD-related patterns of structural co-variation in 91 DN subregions, which were in lockstep with 38 fine-grained HC subregions. Working at a population scale made it possible for us to investigate the effect of rare genotypes on brain structure. This approach was especially fruitful for the less common *APOE* ɛ2/2 genotype, which has a prevalence of <1% among the general population [22]. Given this setup, our investigation was uniquely positioned to carry out sex-specific examinations across all *APOE* gene variants that previous brain-imaging studies systematically ignored. The availability of deep profiling of the UKB participants further allowed us to chart brain–behaviour associations across the whole phenome in an impartial data-driven approach.

### Population signatures of HC-DN co-variation capture subregion-level structural ties

We first delineated the structural dependencies in regional grey matter volume between the subregion atlas of the HC and that of the DN to identify deviations that jointly go hand-in-hand. We benefitted from canonical correlation analysis (CCA), a doubly multivariate pattern-learning tool (cf. methods), to identify the sources of common population variation between the full sets of 38 HC subregions and that of 91 DN subregions. This algorithmic approach finds principled signatures of structural co-variation between 2 sets of variables [23]. Patterns of shared co-variation (canonical variates, cf. methods*)* embed the effects of HC or DN subregion sets in a new representational space where the 2 sets were most strongly correlated with each other. Pairs of canonical variates, 1 for the HC and 1 for the DN, are what we henceforth call modes. By construction, these are ranked by importance; each mode carries unique information by being uncorrelated from each other. Each mode thus represented a different brain signature that accounted for increasingly less shared variance between the neocortical and allocortical atlas at subregion resolution.

We focused on the leading 25 modes, mode 1 being the most explanatory signature of HC-DN co-variation under the elected model. The explanatory power of a given mode was quantified by Pearson’s correlation between inter-individual variation tracked by its associated HC and DN patterns (canonical correlation, cf. method). The leading signature of HC-DN co-variation (mode 1) achieved a canonical correlation of rho = 0.51, whereas the second and third signatures achieved correlations of rho = 0.42 and 0.39, respectively. Canonical correlations accounted for increasingly less joint variation between the HC and DN subregions up to the last signature (mode 25), which achieved a correlation of rho = 0.06. The full list of correlation coefficients for the remaining modes has been published elsewhere [24] and is openly accessible online (https://figshare.com/articles/figure/Loneliness_Suppplement_July_22_docx/15060684). This multivariate decomposition served as the backbone for all subsequent analyses that aimed to elucidate how individual expressions of HC-DN co-variation varied in relation to ADRD risk.

### Signatures of HC-DN co-variation illuminate concomitant deviations in ADRD risk

To interrogate the neurobiological manifestations of ADRD family history in our UKB cohort, we performed a rigorous group difference analysis that highlighted any statistically robust ADRD-related divergences in each HC-DN population signature. In doing so, we uncovered the precise subset of anatomical subregions contributing to structural HC-DN co-variation that systematically diverged in individuals with versus without family history of ADRD. An HC or DN subregion observed to have a robustly different co-variation expression in individuals with and without family history of ADRD is henceforth termed a hit. We observed a total of 28 HC and 135 DN hits across the leading 25 modes. As a general trend, HC hits were mainly located in the cornu ammonis (CA) subregions (42.9% of total divergences). Parallel DN hits were predominantly observed in the prefrontal cortex (dorsomedial prefrontal cortex (dmPFC) and ventrolateral prefrontal cortex (vlPFC); 45.9% of total divergences) and posterior midlines structures (posterior cingulate cortex (PCC), precuneus (PCu), and retrospenial cortex (RSC); 27.4% of total divergences).

**Fig 1:**
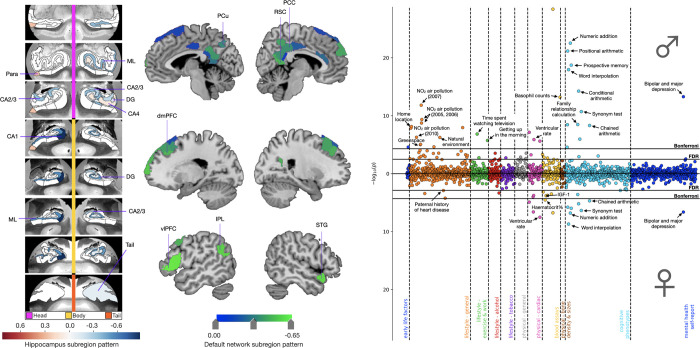
Cognitive, environmental, and cardiovascular phenotypes show sex-specific associations with *APOE* dosage in the context of mode 1. The leftmost and central panels display structural divergences in the HC and DN, respectively, on mode 1 for the group difference analysis of ADRD family history. We identified 12 HC hits, mostly located in the CA subfields and molecular layer. We also showed 34 DN hits, most of them located in the prefrontal cortex and midline structures. In separate analyses for males (*N* = 17,561) and females (*N* = 19,730), *APOE* dosage was regressed on HC and DN co-variation patterns from mode 1. We then used these sex-specific models to predict *APOE* dosage based on inter-individual expressions of mode 1. *APOE* dosage predicted for each individual was then correlated to 977 UKB phenotypes in separate analyses for males and females. The rightmost panel displays the Miami plot for the correlations between predicted *APOE* dosage in the context of mode 1 and UKB traits. The upper and lower part of the Miami plot displays the correlations for males and females, respectively. The y-axis indicates negative decimal logarithms for the *p*-values of each correlation represented by a dot. We highlight important brain–behaviour associations between *APOE* dosage pooled across subject-specific expressions of mode 1 and verbal-numerical reasoning, supplemented by male-specific correlations with environmental phenotypes. Females showed a specific profile of brain–behaviour associations with cardiovascular phenotypes (e.g., systolic and diastolic blood pressure, IGF-1, and urea) that extended beyond physical traits shared with males (e.g., cardiorespiratory fitness and ventricular and pulse rate). Data underlying this figure can be found at https://github.com/dblabs-mcgill-mila/HCDMNCOV_AD/tree/master/Miami_Plots (DOI: 10.5281/zenodo.7126809). ADRD, Alzheimer’s disease and related dementia; *APOE*, Apolipoprotein E; CA, cornu ammonis; DG, granule cell layer of the dentate gyrus; dmPFC, dorsomedial prefrontal cortex; DN, default network; FDR, false discovery rate correction; HC, hippocampus; IPL, inferior parietal lobule; ML, molecular layer; Para, parasubiculum; PCC, posterior cingulate cortex; PCu, precuneus; RSC, retrosplenial cortex; STG, superior temporal gyrus; vlPFC, ventrolateral prefrontal cortex.

**Fig 2:**
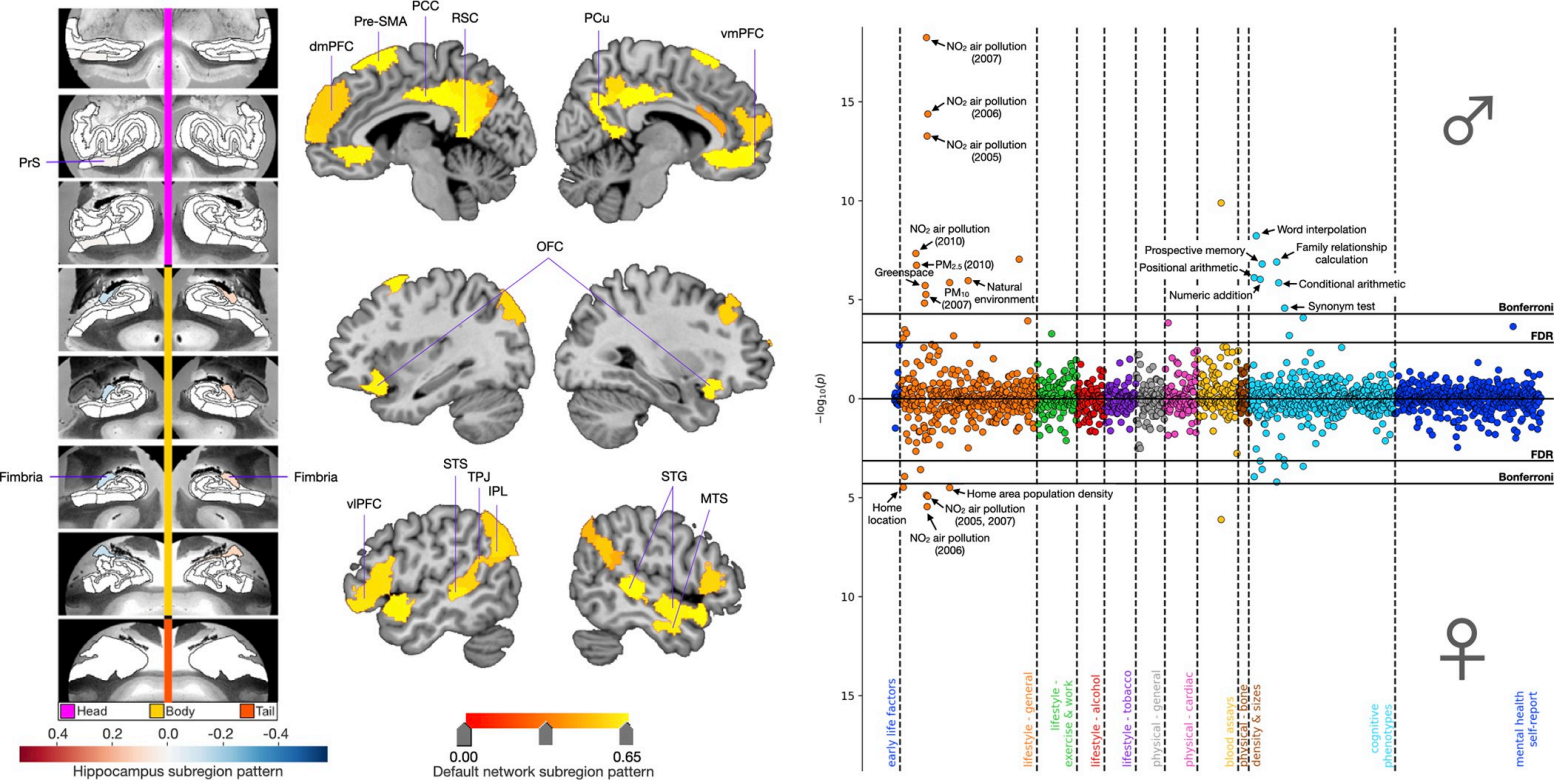
*APOE* associations for mode 3 revealed a prominence of cognitive and environmental phenotypes in males. Shown here are ADRD-related subregion divergences for mode 3 for the HC (leftmost panel) and DN (central panel). We identified focalized hits to the fimbria and presubiculum with corresponding grey matter differences across the whole DN. In males and females separately, we regressed *APOE* dosage on HC and DN co-variation patterns from mode 3. We then used these sex-specific models to predict *APOE* dosage based on inter-individual expressions of mode 3. The rightmost panel displays the Miami plot for the correlations between *APOE* scores in the context of mode 3 and the portfolio of UKB phenotypes for males (upper half) and females (lower half). We highlighted significant associations with environmental phenotypes that were again more prominent in males than females. We additionally showed significant correlations with the fluid intelligence battery that were male specific. Data underlying this figure can be found at https://github.com/dblabs-mcgill-mila/HCDMNCOV_AD/tree/master/Miami_Plots (DOI: 10.5281/zenodo.7126809). ADRD, Alzheimer’s disease and related dementia; *APOE*, Apolipoprotein E; dmPFC, dorsomedial prefrontal cortex; FDR, false discovery rate correction; IPL, inferior parietal lobe; MTS, middle temporal sulcus; OFC, orbitofrontal cortex; PCC, posterior cingulate cortex; Pre-SMA, pre-supplementary motor area; PrS, presubiculum; PCu, precuneus; RSC, retrosplenial cortex; STG, superior temporal gyrus; STS, superior temporal sulcus; TPJ, temporoparietal junction; vlPFC, ventrolateral prefrontal cortex; vmPFC, ventromedial prefrontal cortex.

**Fig 3:**
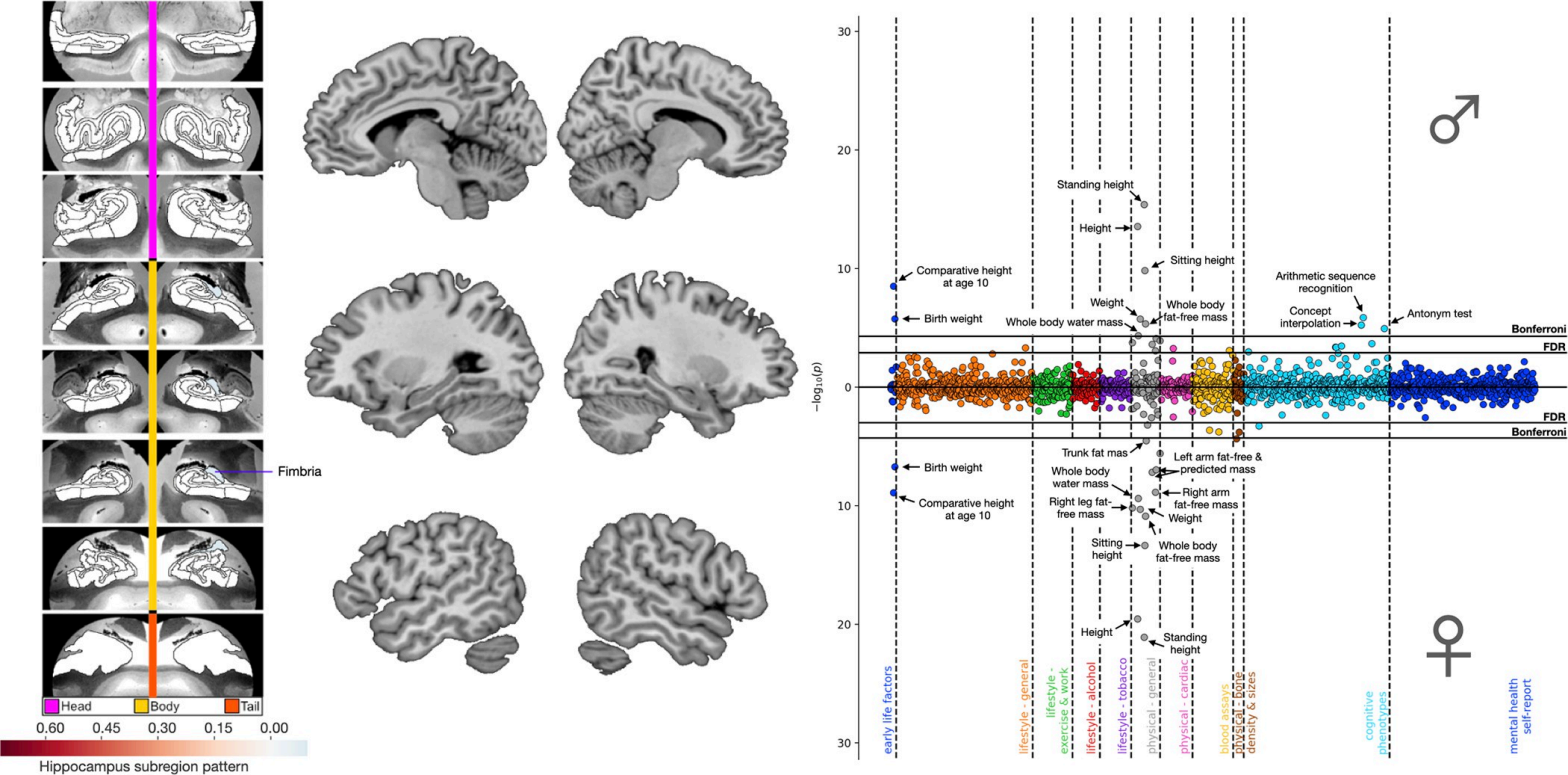
*APOE* associations for mode 8 linked lipid metabolism to deviation of the fimbria. Shown here are ADRD-related subregion divergences for mode 8 for the HC (leftmost panel) and DN (central panel). We identified a focalized divergence to the fimbria with no corresponding DN hits. In males and females separately, we regressed *APOE* dosage on HC and DN co-variation patterns from mode 8. We then used these sex-specific models to predict *APOE* dosage based on inter-individual expressions of mode 8. The rightmost panel displays the Miami plot for the correlations between *APOE* scores in the context of mode 8 and the portfolio of UKB phenotypes for males (upper half) and females (lower half). We show associations with phenotypes related to lipid metabolism and height, supplemented by male-specific associations with the fluid intelligence battery. Data underlying this figure can be found at https://github.com/dblabs-mcgill-mila/HCDMNCOV_AD/tree/master/Miami_Plots (DOI: 10.5281/zenodo.7126809). ADRD, Alzheimer’s disease and related dementia; *APOE*, Apolipoprotein E; DN, default network; FDR, false discovery rate correction; HC, hippocampus.

In mode 1, we identified 12 HC hits as indicative for family history of ADRD, with the strongest subregion effects identified in CA1, CA2/3, molecular layer, and granule cell layer of the dentate gyrus (DG) (66.7% of HC divergences in mode 1). The remaining HC hits for mode 1 were either located in the parasubiculum, CA4 or HC tail (Fig 1). We revealed 34 concomitant DN hits, most of them located in the prefrontal cortex (dmPFC and vlPFC) and posterior midline structures (RSC, PCC, and PCu) that represented 55.9% and 35.3% of total DN hits in mode 1, respectively. As for mode 2, 80.0% of the 10 identified HC hits were located in the left hemisphere (S1 Fig). Of those hits, the strongest weights were found in the presubiculum and CA2/3. The remaining HC hits were identified in the CA1, CA4, hippocampal fissure, and DG. While the majority of the 30 DN divergences for mode 2 were located in the prefrontal cortices (dmPFC; 30.0%) and posterior midline structures (PCC and RSC; 26.6%), a substantial proportion of hits were located in the temporal and posterior cortices. In particular, 23.3% of DN divergences for mode 2 were located in the temporal cortices (superior temporal sulcus (STS), middle temporal sulcus (MTS), and temporal pole) compared to 20.0% to the left posterior cortex (inferior parietal lobule (IPL) and superior parietal lobule (SPL)). Mode 3 in turn showed 3 statistically relevant HC hits to the fornix’s fimbria and presubiculum, in concordance with 56 DN divergences (Fig 2). Of the DN hits identified for mode 3, 35.7% were located in the frontal lobe (dmPFC, ventromedial prefrontal cortex (vmPFC), vlPFC, pre-supplementary motor area (pre-SMA), and OFC), 30.3% to posterior midline structures (PCC, RSC, and PCu), 17.9% to the temporal cortices (STS, MTS, and superior temporal gyrus (STG)), and 16.1% to the parietal cortices (IPL, SPL, and temporoparietal junction (TPJ)). A minority of the modes only showed HC hits, either located in the fimbria (mode 8; Fig 3) or in the hippocampus–amygdala transition area (modes 6 and 10; S2 and S3 Figs) without any concomitant DN hits. Inversely, some modes only showed DN divergences in the absence of HC hits. This was the case for mode 4 for which we identified 4 DN hits in the dmPFC (S4 Fig), mode 7 for which 9 DN hits were identified in the PFC (dmPFC and OFC; S5 Fig), mode 11 for which 1 DN hit was identified in the PCC (S6 Fig), and mode 13 for which 1 DN hit was identified in the STS (S7 Fig).

Across HC-DN co-variation signatures, we noted a prominence of HC structural deviation in the CA1, CA2/3, and fimbria for the group analysis of ADRD risk. As for the DN divergences, we highlighted a constellation of structural deviations involving the prefrontal cortices and posterior midline structures. Modes 1 and 2 showed the highest relative numbers of HC divergences (i.e., 12 and 10 hits, respectively) as compared to any other modes. While the third signature of HC-DN co-variation only showed 3 statistically relevant HC hits, it showed the highest relative number of DN divergences. Together with mode 8, the focalized divergences found in the fimbria for mode 3 highlighted the importance of the fornix in ADRD risk. We further uncovered concomitant structural divergences in HC and DN subregions with known direct anatomical connections in macaque monkeys, such as the presubiculum with RSC [16] and molecular layer with OFC/vmPFC [17]. Ultimately, we revealed an intertwined collection of structural divergences in highly coupled HC and DN subregions that have been linked to ADRD risk and progression by previous research, such as the CA1, CA2/3, presubiculum, and the fornix’s fimbria [13,25–28], as well as the dlPFC, OFC, PCC, and PCu [29–32].

### Phenome-wide fingerprints of brain–behaviour associations uncover sex-specificity in ADRD risk

We next conducted a phenome-wide analysis to systematise domains of UKB traits in terms of their association with HC-DN signatures and ADRD risk. To quantify genetic risk, we created a bivariate dosage scale that tested for the opposing effects of *APOE* ɛ2, often suspected to confer protective benefits [33], and ɛ4, classically believed to escalate dementia risk [4]. We fitted linear regression models to relate inter-individual expressions of HC-DN co-variation from the 25 signatures to subject-level *APOE* ɛ2 versus ɛ4 dosage. Subject-level *APOE* dosage was predicted from a collection of HC-DN signatures using these linear models and subsequently tested against 977 curated UKB phenotypes in a phenome-wide assay conducted separately in males and females. Only the top 3 modes with the most brain–behaviour associations across sexes, i.e., modes 1, 3, and 8, are presented below (Figs 1–3). The phenome-wide profiles for each of the remaining modes with statistically defensible deviations with respect to family history of ADRD are available as part of the online Supporting information (S1–S7 Figs).

The phenome-wide profile for mode 1 highlighted brain–behaviour associations with cognitive traits in addition to male-specific correlations with environmental phenotypes (Fig 1). After carrying out Bonferroni’s correction for multiple comparisons, *APOE* dosage pooled across subject-specific expressions of mode 1 yielded 31 and 13 significant associations in males and females, respectively. Cognitive traits represented 35.5% of significant mode–trait associations in males and 53.8% of those identified in females. Baseline cognitive performance on the fluid intelligence battery accounted for most of the cognitive associations, with 7 questions yielding significant associations in males compared to 6 in females. Significant associations with baseline prospective memory were also identified for both sexes, measured as the correct recalling of the object previously shown to participants on the screen. The phenome-wide profiles for both sexes also included ventricular rate on electrocardiogram measured at rest, the completion status of electrocardiogram during exercise, and bipolar and major depression status. At the more lenient false discovery rate (FDR) correction, we observed additional phenotypes linked with erythrocytes count for both sexes. The second most dominant sets of associations for mode 1 centred on environmental phenotypes, such as NO_2_ exposure, natural environment, and greenspace, representing 29.0% of significant mode–trait correlations identified in males. Other male-specific associations included lifestyle (time spent watching television and difficulty waking up in the morning) and physiological (hand grip strength, arm mass, and height) phenotypes. At the more lenient FDR correction, males showed additional brain–behaviour associations including exposure to particulate matter of 2.5 μm and 10 μm or less in diameter (PM_2.5_ and PM_10_). After applying Bonferroni’s correction, females showed unique associations with diastolic blood pressure and hematocrit percentage. When applying FDR correction, additional cardiovascular phenotypes showed significant associations in females, such as a paternal history of heart attack, systolic blood pressure, insulin-like growth factor 1 (IGF-1), and haemoglobin concentration. In sum, our phenotypical profiling assay highlighted important phenome-wide associations between *APOE* dosage pooled across subject-specific expressions of mode 1 and verbal-numerical reasoning, supplemented by male-specific correlations with environmental phenotypes. Females instead showed a specific profile of brain–behaviour associations with cardiovascular phenotypes that extended beyond physical traits shared with males.

In the phenome-wide profile for mode 3, we uncovered brain–behaviour associations with cognitive and environmental phenotypes, again more prominent in males than females (Fig 2). After Bonferroni’s correction, *APOE* dosage in the context of mode 3 expressions yielded 19 and 6 significant mode–trait associations in males and females, respectively. Environmental phenotypes represented 52.6% of significant associations in males and 83.3% of those identified in females. Significant associations with NO_2_ exposure and home area population density were observed for both sexes. Males also showed significant associations with baseline cognitive performance on 6 questions from the fluid intelligence battery as well as with baseline prospective memory. Females did not show significant associations beyond those shared with males, with the exception of home location. At the more lenient FDR correction, females showed additional associations with prospective memory and baseline cognitive performance on 5 questions from the fluid intelligence battery. As such, *APOE* dosage pooled across subject-specific expressions of mode 3 allowed us to uncover a rich portfolio of associations with environmental and cognitive phenotypes that were more robust in males than females.

In comparison to the overlapping portfolio of brain–behaviour associations derived from modes 1 and 3, the phenome-wide profile for mode 8 emphasised a unique set of physiological phenotypes (Fig 3). After Bonferroni’s correction, *APOE* dosage pooled across subject-specific expressions of mode 8 yielded 11 and 15 significant mode–trait associations in males and females, respectively. Physical phenotypes related to body mass and height represented 55.5% of significant correlations in males and 80.0% of those identified in females. After Bonferroni’s correction, males showed significant associations with cognitive performance on 3 questions from the fluid intelligence battery assessed in the online follow-up. At the more lenient FDR correction, males showed further associations with cognitive performance on 2 additional questions from the fluid intelligence battery and with the maximum number of digits remembered correctly on the numeric memory test, both assessed in the online follow-up. After Bonferroni’s correction, females showed significant associations with trunk fat mass and heel bone mineral density. In sum, we highlighted important phenome-wide associations between *APOE* dosage pooled across subject-specific expressions of mode 8 and proxies of cardiovascular health, supplemented by male-specific correlations with cognitive phenotypes. A formal assessment of the difference in associations between males and females for the 3 modes with the most brain-phenotypic associations across sexes (i.e., modes 1, 3, and 8) is presented in the Supporting information (S8–S10 Figs) and serves as a complement to their respective Miami plots (Figs 1–3) (cf. methods). The phenome-wide profiles derived across these 3 concomitant regimes of HC-DN co-variation emphasised sex differences in ADRD risk, with recurring associations with air pollution and verbal-numerical reasoning that were more prominent in males than females.

### *APOE* gene variants are associated with distinct clusters of risk-anatomy links

**Fig 4:**
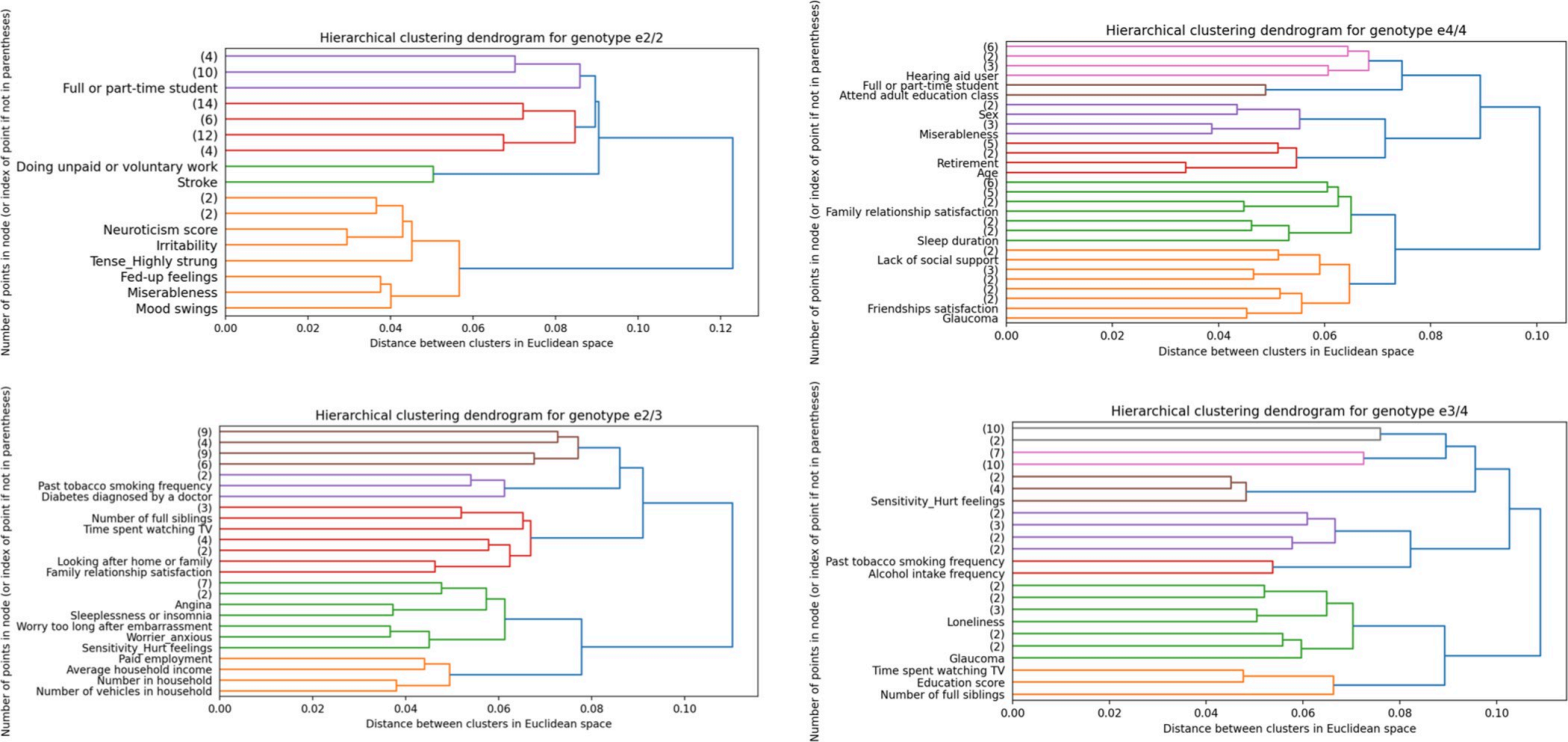
Neuroticism-related phenotypes show unique risk-anatomy links in ɛ2 carriers. To test for risk-anatomy links, we computed the Spearman’s correlations between the population-wide HC and DN co-variation patterns, multiplied by each of the 6 *APOE* genotypes and the 63 preselected Alzheimer’s disease risk factors [34]. We performed an agglomerative clustering analysis on these Spearman’s correlations, which consists of repeatedly merging Spearman’s correlations with similar variance until all observations are merged into a single cluster. Here are shown the dendrograms that indicate the distance between each cluster identified when retaining 3 levels of branching for *APOE* ɛ2/2 (upper left; *N* = 217), ɛ2/3 (lower left; *N* = 4,625), ɛ4/4 (upper right; *N* = 822), ɛ3/4 (lower right; *N* = 8,613). The dendrograms for ɛ3/3 and ɛ2/4 can be found in the Supporting information (S12 Fig). We showed the early emergence of social engagement phenotypes (e.g., doing unpaid or voluntary work, attending adult education classes, family relationship satisfaction, number of people in household, and number of full siblings) across the different *APOE* gene variants suggesting that the contribution of social behaviours to risk-anatomy links transcend genetic risk. Ɛ3 carriership was characterised by the early branching of socioeconomic determinants (e.g., paid employment, average household income, number of vehicles in the household, time spent watching TV, and education score) as shown on the dendrograms for ɛ2/3, ɛ3/4, and ɛ3/3 (S12 Fig). While clusters of social engagement and socioeconomic determinants were shared across different *APOE* genotypes, we found that neuroticism was uniquely associated with ɛ2 carriership. Indeed, the dendrogram for ɛ2/2, ɛ2/3, and ɛ2/4 (S12 Fig) showed an early emerging cluster of neuroticism-related phenotypes (e.g., irritability, miserableness, being worried/anxious). This personality cluster was especially apparent for ɛ2 homozygotes, as reflected by the relatively high Euclidean distance of the first branching that split the neuroticism-related phenotypes from the rest of the risk factors. Data underlying this figure can be found at https://github.com/dblabs-mcgill-mila/HCDMNCOV_AD/tree/master/clustering_analysis (DOI: 10.5281/zenodo.7126809). *APOE*, Apolipoprotein E; DN, default network; HC, hippocampus.

We next examined ADRD-specific clusters of risk-anatomy links across each unique *APOE* gene variant (i.e., ɛ2/2, ɛ2/3, ɛ3/3, ɛ2/4, ɛ3/4, and ɛ4/4). We computed the interactions between the subject-specific expressions of HC-DN co-variation modes (canonical variates) and each *APOE* genotype (encoded as binary variables, such that participants who do not carry a given genotype were zeroed out). In doing so, we obtained 6 new population-wide indices, 1 for each distinct *APOE* genotype that we correlated, using Spearman’s coefficient, with 63 curated ADRD risk factors (a phenotype collection used previously [34]). We then performed an agglomerative clustering analysis that consisted of a nested merging of correlation coefficients with similar variance until all observations merged in a single cluster. The ensuing dendrograms indicated the distance between each cluster identified when retaining 3 levels of branching (Fig 4). A formal metric of statistical agreement between cluster models was provided as part of supplementary analyses (S11 Fig).

Our integrated analysis of risk-anatomy links showed the relatively early branching of social engagement phenotypes for ɛ2/2 (e.g., being a full- or part-time student and doing unpaid or voluntary work), ɛ2/3 (e.g., number of full siblings, looking after one’s home or family, family relationship satisfaction, and number of people in household), ɛ3/4 (e.g., number of full siblings), and ɛ4/4 (e.g., being a full- or part-time student, attending adult education classes, retirement, family relationship satisfaction, lack of social support, and friendships satisfaction) genotypes. The relevance of social engagement phenotypes across most *APOE* gene variants suggests that the contribution of social behaviours to risk-anatomy links transcend genetic risk. Ɛ3 carriership was characterised by the early branching of socioeconomic determinants as shown on the dendrograms for ɛ2/3 (e.g., past tobacco smoking frequency, time spent watching television, paid employment, average household income, and the number of vehicles in the household), ɛ3/4 (e.g., past tobacco smoking frequency, alcohol intake frequency, time spent watching television, and education score), and ɛ3/3 (time spent watching television, education score, past and current tobacco smoking frequency, alcohol consumption on a typical drinking day and alcohol intake frequency; see S12 Fig). We noted the early emergence of a personality cluster in ɛ2 carriers that comprised self-reported traits related to neuroticism as shown on the dendrograms for ɛ2/2 (e.g., irritability, miserableness, mood swings), ɛ2/3 (e.g., being worried/anxious and easily hurt), and ɛ2/4 (e.g., being worried/anxious, mood swings, and miserableness; see S12 Fig). All these personality traits have been identified as neurotic behaviour domains and are part of the neuroticism battery of the UKB (UKB data field 20127). We thus uncovered that neuroticism, which is known to be closely linked to loneliness [35], is a personality trait that shows distinct patterns of association with HC-DN co-variation expressions in ɛ2 carriers.

### Sex-specific dependencies between *APOE* gene variants and signatures of HC-DN co-variation explain ADRD risk

**Fig 5:**
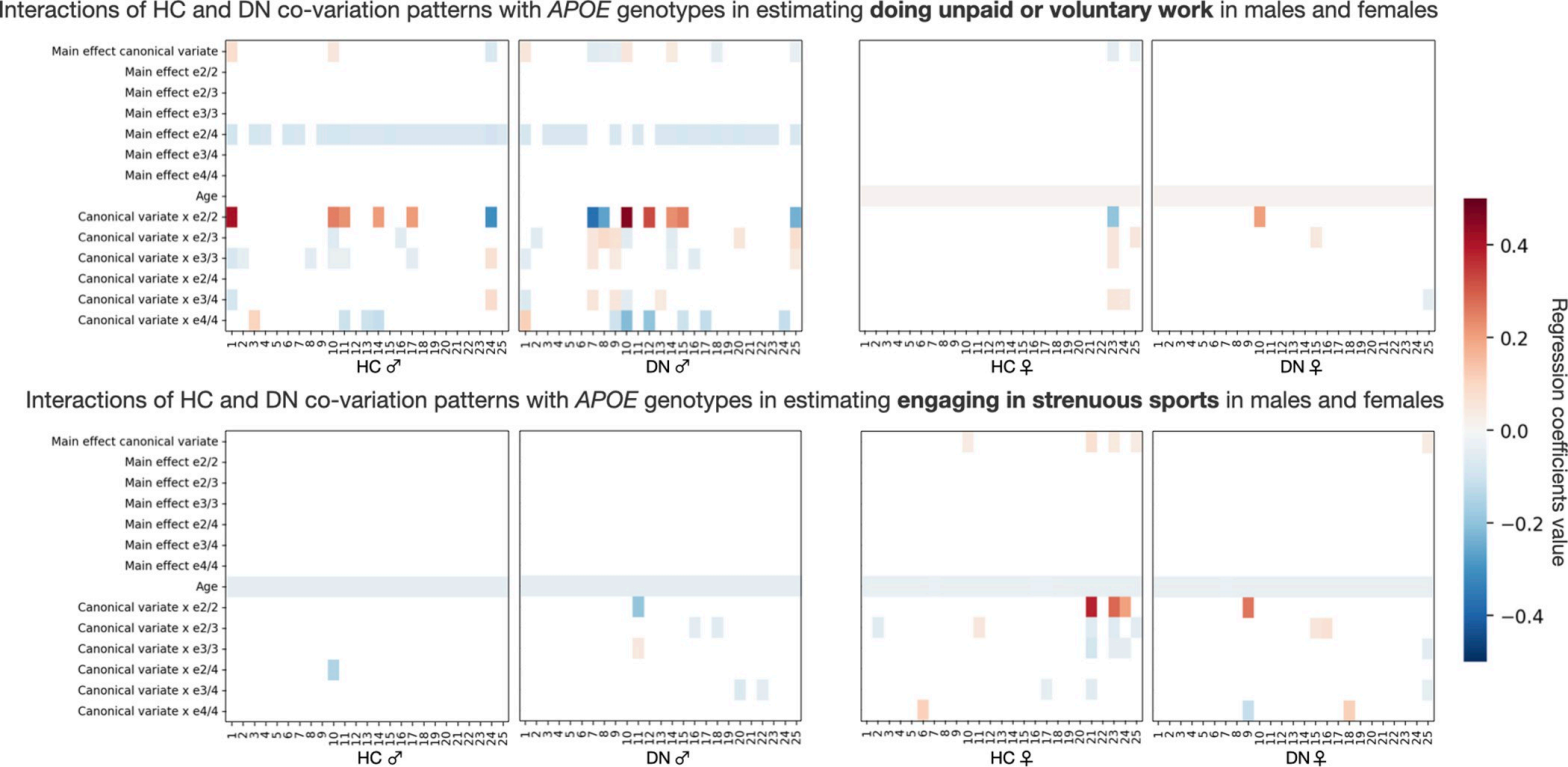
Brain-*APOE* ɛ2/2 interaction explains variance in social lifestyle in males and physical activity in females. We tested whether HC-DN signatures interacted with *APOE* genotypes in explaining variance on the 63 preselected ADRD risk factors. Separate analyses were run for males (leftmost plots) and females (rightmost plots). Each column on the heat maps represents the coefficients for a single linear regression model. The first 25 columns show the coefficients for HC patterns, whereas the last 25 columns show the coefficients for DN patterns. We assessed the robustness of our findings by comparing each coefficient to empirically built null distributions obtained through permutation testing. Only the coefficients that were statistically different from their respective null distributions 95% of the time are presented. We displayed the modifiable risk factors for which the strongest brain-*APOE* interactions were observed. In the top panels, we show that *APOE* ɛ2/2 preferentially interacts with HC and DN canonical variates in estimating doing unpaid or voluntary work in males. In the bottom panels, we show that *APOE* ɛ2/2 interacts with selective HC and DN canonical variates in estimating engaging in strenuous sport in females. We have thus shown that *APOE* ɛ2/2 interacts preferentially with HC-DN co-variation patterns in estimating social lifestyle in males and physical activity in females. These interactions profiles suggest that ɛ2, and not ɛ4, is driving most of the brain–*APOE* interactions in healthy individuals at risk of developing ADRD with a substantial level of sex-specificity. Data underlying this figure can be found at https://github.com/dblabs-mcgill-mila/HCDMNCOV_AD/tree/master/fig_5 (DOI: 10.5281/zenodo.7126809). ADRD, Alzheimer’s disease and related dementia; *APOE*, Apolipoprotein E; DN, default network; HC, hippocampus.

We next directed attention to sex-specific interactions between HC-DN co-variation regimes and *APOE* genotype status that would explain inter-individual differences in ADRD risk. To this end, we tested whether HC-DN signatures systematically interacted with specific *APOE* genotypes in explaining variation in a collection of 63 ADRD risk factors (cf. above). More formally, each risk factor was individually regressed on the subject-specific expressions of HC and DN patterns for each of the 25 modes. This analysis step hence supplied 50 estimated linear models per target risk factor. Each model took as input variables the main effect of the HC or DN pattern expressions, the main effects of the 6 *APOE* genotypes, and the interaction between each *APOE* genotype with the HC or DN pattern, controlling for age. Separate analyses were carried out in the male (Fig 5, leftmost panels) and female (Fig 5, rightmost panels) subgroups of our UKB cohort. To ascertain the robustness of our findings, we compared each coefficient estimate against empirically data-derived null distributions obtained through a rigorous permutation procedure (i.e., label shuffling permutation). We only interpreted the model coefficients that emerged as statistically relevant based on the respective null distributions at 95% confidence.

In a comprehensive set of analyses across 63 ADRD risk factors, we identified the strongest nonlinear interaction effects in homozygote ɛ2 carriers. Notably, brain-*APOE* interactions accounted for more variance in several modifiable social and cardiovascular risk factors than did the main effects of *APOE* ɛ2 and ɛ4. Male ɛ2 homozygotes showed strong interactions with HC and DN pattern expressions for doing unpaid or voluntary work. In parallel, female ɛ2 homozygotes showed strong interactions with HC-DN pattern expressions for engagement in strenuous sports. Across the different domains of risk factors investigated, we singled out brain-*APOE* interactions specific to ɛ2 homozygotes that were not identifiable in heterozygotes and non-carriers. While we observed no appreciable sex effect for the interaction of *APOE* ɛ4/4 and HC-DN co-variation expressions, we found defensible sex-specificity for the role of *APOE* ɛ2/2. More precisely, we showed strong interactions between *APOE* ɛ2/2 and HC-DN co-variation patterns for social lifestyle factors in males and physical activity factors in females. Through our analyses of a variety of risk factors, we have thus isolated brain-*APOE* interactions unique to ɛ2 carriers that depend on sex.

**Fig 6:**
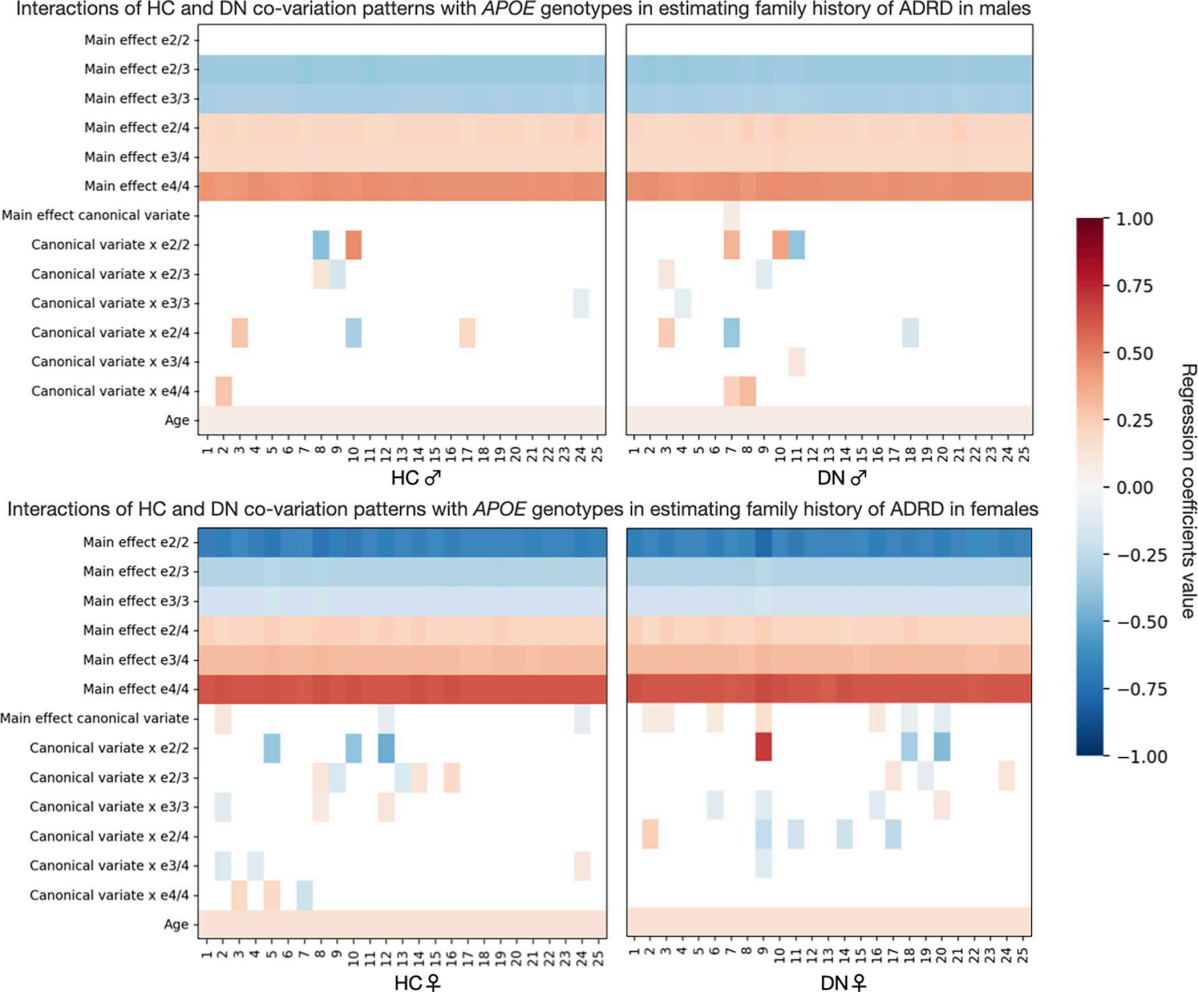
The protectiveness of ɛ2 is sex-dependent and modulated by HC-DN co-variation patterns. In separate analyses for males and females, we tested whether HC-DN signatures interacted with *APOE* genotypes in explaining variance in family history of ADRD. Separate analyses were run for males (higher plots) and females (lower plots). Each column on the heat maps represents the coefficients for a single linear regression model. The first 25 columns show the coefficients for HC patterns, whereas the last 25 columns show the coefficients for DN patterns. We assessed the robustness of our findings by comparing each coefficient to empirically built null distributions obtained through permutation testing. Only the coefficients that were statistically different from their respective null distributions 95% of the time are presented. We found that the main effect of *APOE* ɛ2/2 against ADRD risk was only statistically robust in females. We also showed a spectrum in the opposing effects of ɛ2 and ɛ4 among females, such that ɛ2/4 was associated with a lower increase in ADRD risk than did *APOE* ɛ3/4, which in turn was associated with lesser risk than ɛ4/4. We further found that the protectiveness of *APOE* ɛ2/2 interacts with brain structure and can even lead to an increase in ADRD risk among females with a strong expression of mode 9. These interactions profiles suggest that the protectiveness of ɛ2/2 is not only sex-specific but also modulated by HC-DN co-variation expressions. Data underlying this figure can be found at https://github.com/dblabs-mcgill-mila/HCDMNCOV_AD/tree/master/fig_6 (DOI: 10.5281/zenodo.7126809). ADRD, Alzheimer’s disease and related dementia; *APOE*, Apolipoprotein E; DN, default network; HC, hippocampus.

After examining target risk factors, we next put to the test whether expressions of HC-DN signatures bear relations with *APOE* genotypes in explaining ADRD risk. In dedicated analyses for males (Fig 6, upper panels) and females (Fig 6, lower panels), family history of ADRD was regressed on a single HC or DN pattern, resulting in 50 different linear models per sex. Each such model was fed as input variables the main effect of the HC or DN pattern, the main effects of the *APOE* genotypes, and the interactions between each *APOE* genotype and the HC or DN pattern, controlling for age. We assessed the robustness of our findings by comparing each coefficient to empirically built null distributions obtained through permutation testing (cf. above). We focused interpretation on the model coefficients that were statistically robust against their respective null distributions at 95% confidence. We found no statistically relevant main effect of *APOE* ɛ2/2 on ADRD risk among males. For *APOE* ɛ2/3 and ɛ3/3 carriers, we found similar effects on ADRD risk in males, lowering the odds of ADRD family history by approximately 30% across the different models investigated. Likewise, *APOE* ɛ2/4 and ɛ3/4 carriers showed similar effects in tracking ADRD risk in males, elevating the odds of ADRD family history by more than 20% on average. As expected from the literature, *APOE* ɛ4/4 increased the odds of ADRD family history by more than 56% in males across the different models investigated. In females, *APOE* ɛ2/2 status decreased the odds of ADRD family history by 50% on average, while ɛ2/3 and ɛ3/3 status led to decreases of approximately 25% and 17%, respectively. In contrast, *APOE* ɛ3/4 and ɛ4/4 status lifted the odds of ADRD family history by approximately 35% and 86%, respectively. Among females, *APOE* ɛ2/4 carriers were associated with dampened ADRD risk relative to *APOE* ɛ3/4 carriers. The odds of ADRD family history associated with *APOE* ɛ2/4 were only increased by 24% in females. This approximately 10% reduction in ADRD risk, uniquely observed among females, could be taken to suggest that ɛ2 can still be protective against ADRD risk in the presence of an ɛ4 allele. Females also showed some strong brain-*APOE* interactions above and beyond the well-established risk and protective effects associated with each *APOE* genotype. Notably, the interaction of mode 9 DN pattern expressions with *APOE* ɛ2/2 status was associated with a 2-fold increase in ADRD risk. It was considerably stronger than the main risk effect conferred by *APOE* ɛ4/4. This strong interaction effect can be taken to suggest that HC-DN co-variation plays a chief role in ADRD risk, which might have been overlooked by previous analyses restricted to genetic data. In sum, we identified and annotated sex-specificity in the opposing effects of ɛ2 and ɛ4 on ADRD risk, with demonstrably stronger brain-*APOE* interactions among females.

### Dominant principles of brain–behaviour associations uncovered a male-specific link with neuroticism

In a final suite of analyses, we conducted an exploratory principal component analysis (PCA) to disentangle the major sources of brain–behaviour variation in our UKB cohort. We first computed Pearson’s correlations between the 25 pairs of expressions (i.e., canonical variates) from the HC and those from the DN patterns and the 63 preselected ADRD risk factors. This step yielded 3,150 distinct coefficients represented by a risk by canonical variates matrix (X_63 × 50_). We then carried out a PCA to reduce the dimensionality to 3 major axes of brain-behaviour variation. These explained approximately 13.8%, approximately 9.6%, and approximately 8.2% of the total variance in the cross-correlation matrix, respectively (S13 Fig).

The leading axis of variation highlighted social phenotypes previously singled out in the clustering analysis (e.g., attending religious groups, attending adult education classes, and the number of people in the household). We also observed a strong expression of socioeconomic determinants among the first axis of brain–behaviour associations (e.g., age completed high school education, average household income, paid employment, and the number of vehicles in the household). The second most important axis mainly emphasised health-related phenotypes (e.g., stroke, hypertension, and diabetes) and lifestyle factors (e.g., alcohol intake frequency, difficulty getting up in the morning, being a morning person, and sleeplessness). The third most explanatory axis tracked neuroticism and its associated personality trait indicators (being worried/anxious, being easily hurt, and worrying too long after embarrassment) from the rest of the risk factors. We again emphasised the importance of social factors on HC-DN co-variation expressions along with other socioeconomic and lifestyle behaviours.

To certify the robustness of our findings, we performed a split-half reliability assessment of our principal component solution across 1,000 bootstrap iterations. At each iteration, we drew 37,291 participants with replacements to simulate random participant samples that we could have pulled from the same population. We then randomly split the sample in half to create 2 analogous subsets. We computed the Pearson’s correlation between possible pairs of the 50 canonical variates and 63 phenotypes across participants for each random subset. We then estimated 2 PCA models in parallel, 1 for each random half subset, on the z-scored correlation coefficients matrices (63 phenotypes × 50 canonical variates). We showed the average projection of each Pearson’s correlation coefficient on the 3 principal axes of brain–behaviour associations across the 1,000 iterations. We found that the projections of the risk-anatomy link on component 1 were robust. While of lesser strength than the first axis of brain–behaviour associations, the projections for components 2 and 3 are reminiscent of the original analysis. In particular, neuroticism-related personality traits are distinctly expressed on the third axis of brain–behaviour associations, as was found in our original analysis (S14 Fig). A formal account of statistical agreement between both subsets was provided as part of the Supporting information (S15 Fig).

We then repeated the identical pattern-learning workflow sex-stratifying in males and females separately. The top 3 principal components explained approximately 12.1%, approximately 9.9%, and approximately 9.0% of the total variance in males, and approximately 13.1%, approximately 9.5%, and approximately 7.3% of the total variance in females (S16 Fig). The first axis of brain–behaviour associations was roughly the same in males and females as in our original analysis. In fact, the same set of social phenotypes was emphasised on component 1 (e.g., attending religious groups, attending adult education classes, and the number of people in the household) for both sexes. In contrast, component 2 separated neuroticism-related items (e.g., miserableness, fed-up feelings, mood swings, and being worrier/anxious) from the rest of the risk factors in males only. The fact that the neuroticism-related component was the second most important axis of brain–behaviour associations in males but was found in third place on the whole population-derived PCA suggests that the association between neuroticism-related phenotypes and HC-DN co-variation expressions was most important in males. Lastly, the third axis of brain–behaviour associations emphasised different categories of risk factors in males and females. The male-derived component 3 emphasised socioeconomic determinants (e.g., education score and the number of vehicles in the household). In contrast, the female-derived one emphasised lifestyle risk factors (e.g., alcohol intake frequency, alcohol consumption on a typical drinking day, and past tobacco smoking frequency). Our sex-specific analysis hence revealed that the first and most robust axis of brain–behaviour associations was shared across sexes, whereas the second and third axis emphasised sexually dimorphic groups of risk factors.

We performed a bootstrap analysis of the sex-specific PCA solutions to assess the robustness of our findings. Across 1,000 bootstrap iterations, we drew 17,561 males and 19,730 females with replacements to simulate random participant samples that we could have gotten. At each iteration, we computed the Pearson’s correlation between possible pairs of the 50 canonical variates and the remaining 62 phenotypes (as sex was used for grouping) across males and females separately. We then estimated 2 PCA models in parallel, 1 for each sex, on the z-scored correlation coefficients matrices (62 phenotypes × 50 canonical variates). A formal assessment of statistical agreement in the PCA solutions between both sexes was performed (S17 Fig). We observed a low agreement between the male- and female-derived PCA solutions, thus emphasising the sex-specificity of our derived brain–behaviour axes.

### External validation

To externally validate our discovered associations between HC-DN co-variation signatures and ADRD risk factors, we have investigated whether our UKB-derived population signatures of HC-DN co-variation successfully track ADRD-related variation in unseen participants from an independent sample. We capitalised on the openly available PResymptomatic EValuation of Experimental or Novel Treatments for Alzheimer’s disease (AD) (PREVENT-AD) dataset, one of the largest single-site prospective cohorts of pre-symptomatic individuals with a family history of Alzheimer’s disease. Our final sample included image-derived phenotypes of grey matter morphology and *APOE* SNP genotyping from 318 participants, totaling data from 799 visits. For each visit, we computed the level of expression of each of the 25 HC-DN co-variation signatures, from the UKB, for a participant from PREVENT-AD (cf. methods). To test whether distinct derived modes of HC-DN co-variation track distinct aspects of ADRD-related behaviours in unseen participants, we correlated the individual expressions of the 25 modes, represented by pairs of latent expressions of the UKB-derived brain signatures for the HC and DN sides, with a collection of 157 widely established indicators of ADRD progression (e.g., cerebrospinal fluid and blood biochemistry, cognitive and neurosensory evaluations, and health and demographic profile). We assessed the Pearson’s correlations through permutation testing. We reported only the coefficients that were robustly different from the derived empirical null distribution in at least 95% of the 1,000 permutation iterations (S18 Fig).

We found that the several categories of risk factors that emerged in the phenome-wide profiling in the UKB dataset were also flagged in the PREVENT-AD dataset. For example, we have corroborated a link between individual expressions of mode 1 in PREVENT-AD participants and depression, a phenotype that emerged as statistically significant in the phenome-wide profiling for mode 1 for males and females in the UKB. Similarly, we have replicated associations between mode 2 and verbal-numerical reasoning by linking mode 2 expressions in PREVENT-AD participants to several measures of language fluency and working memory highlighted by the Montreal Cognitive Assessment (MoCA) and Repeatable Battery for Assessment of Neuropsychological Status (RBANS), respectively. The MoCA is a cognitive screening tool specially designed to track mild cognitive impairment [35]. Performance on the MoCA has previously been associated with grey matter volumes in subregions of the HC, including the HATA, in middle-aged patients with diabetes [36]. Looking at the individual expressions of mode 6 in PREVENT-AD subjects, we found robust ties of several sub-items of the MoCa (e.g., attention, subtraction, and language fluency) with the same HC-DN population signature that also showed HATA-specific divergence in the UKB participants.

The phenome-wide profiling for mode 6 further highlighted several indicators of vascular integrity (e.g., carotid intima-media thickness)—a cue to cardiovascular system implication that also emerged in PREVENT-AD participants as reflected by a correlation between mode 6 (on the HC side) and atrial fibrillation. Similarly, the phenome-wide profiling for mode 8 in the UKB highlighted several phenotypes related to body mass, while the expression of mode 8 in PREVENT-AD participants was related to arthritis, a joint disorder worsened by age and weight. In addition to replicating the UKB findings, we found complementarity in the associations between the HC-DN signatures and PREVENT-AD phenotypes such that distinct modes track different domains of ADRD risk. For example, DN variation captured by modes 6 and 8 tracks several global indices of the RBANS, a cognitive battery designed to monitor cognitive decline over time. Notably, only mode 6 tracked the visuospatial dimension of the test, as reflected by correlation with sub-items of the figure drawing tests. Further, only individual expressions of mode 6 in PREVENT-AD participants were also correlated to cognitive performance on the MoCA. These patterns of associations, specific to mode 6, reflect a sensitivity to general cognitive ability in PREVENT-AD participants, who all have a family history of ADRD. We found similar patterns of robust associations to PREVENT-AD phenotypes up to the 25th and last mode of HC-DN co-variation that showed noticeable associations with tau CSF levels on the HC side and cardiovascular factors (e.g., systolic blood pressure, pulse, and *APOE* ε4/4 genotype) on the DN side. We have thus shown that HC-DN signatures robustly track different aspects of ADRD risk in a cohort fully independent from the one in which the co-variation patterns have originally been derived. We have thus corroborated and extended the characterisation of our population-derived limbic-neocortical co-variation signatures by linking them with several known indicators of ADRD risk based on new data.

## Discussion

Longstanding research has insisted on the alteration of the DN and HC in early ADRD development (see, for example, [14]). However, brain-imaging investigations seldom had the opportunity to incorporate rare genotypes such as *APOE* ɛ2/2. At the same time, common epidemiological studies that have reported the protective effect of carrying an ɛ2 allele are not typically equipped to perform an adequately powered brain-imaging examination at a scale of thousands of people. We overcame several shortcomings by capitalising on *APOE* genotyping and structural brain scans from approximately 40,000 UK Biobank participants. Our mission-tailored analytical framework was specially designed for disentangling ADRD-specific differences in brain structure at the population level. Revisiting ADRD through this lens, we uncovered sex-specific associations between rarely investigated *APOE* gene variants and microstructurally defined HC-DN signatures hardly ever discerned in a prospective human cohort. Our collective findings paint a more concrete picture of the antagonistic effects of *APOE* ɛ2 and ɛ4 on population-wide HC-DN signatures, along with their interlocking divergences between men and women.

Epidemiological studies, without access to brain-imaging assessments, have provided evidence suggesting that an ɛ2 allele typically acts to protect against late-onset Alzheimer’s disease [22,33] and against Aβ accumulation [37–42]. Aβ accumulation in ɛ2 carriers could be delayed by 30 to 50 years compared to ɛ4 carriers, who start showing Aβ positivity in their early 40s [12,40,43]. The protective qualities of ɛ2 status have been noted even in the presence of an ɛ4 allele [12]. Nonetheless, the sex-specific impact of *APOE*, especially its ɛ2 gene variants, on brain structure could seldom be investigated at the population level. By deriving an envelope of distinct HC-DN signatures at a fine-grained resolution among thousands of healthy adults, we were able to uncover brain-*APOE* interactions systematically overlooked by traditional brain-imaging studies. Stratifying our population cohort by sex and *APOE* gene variants, we were in a position to conclude that the protective effect of *APOE* ɛ2/2 on ADRD risk was not statistically robust among males, even in a sample of approximately 20,000 participants. In contrast, we demonstrated a spectrum of ɛ2 and ɛ4 effects among females such that *APOE* ɛ2/4 was associated with milder ADRD risk than ɛ3/4, which in turn was associated with milder ADRD risk than ɛ4/4. Resilience towards cognitive decline generally observed among ɛ2 carriers could arise from relatively higher baseline APOE steady-state levels in regions including the HC and frontal cortex as compared to ɛ4 carriers and ɛ3 homozygotes [44–47]. Isoform-specific effects related to the APOE protein could be further enhanced by microglia-driven homeostatic responses to Aβ accumulation [48,49]. In fact, ɛ2 carriers are biologically more efficient at scavenging Aβ [50]. As a result, Aβ positivity in ɛ2 carriers with normal cognition is generally detected in much older age (approximately 95 years) as in ɛ4 carriers (40 to 55 years) [40]. Older ɛ2 carriers with amyloid pathology are likewise less likely to be diagnosed with dementia than ɛ3 homozygotes of the same age [51]. Cell proliferation and survival in the HC are thought to be particularly modulated by estrogens [52–54] that could have a downstream impact on microglial and astrocytic APOE synthesis [55]. The presence of an estrogen-dependent enhancer in the promoter region of the *APOE* gene is thus bound to favour female ɛ2 carriers [56]. These previous elements of evidence are in line with our present finding suggesting that the protective effect of *APOE* ɛ2 on ADRD risk is sex specific and also unique to particular HC-DN co-variation patterns. Notably, we found that female ɛ2 homozygotes with a high expression of mode 9 had twice the odds of having a family history of ADRD. We have thus shed light on important nuances in the predominant genetic account of ADRD by questioning the protectiveness of ɛ2 when placed in relation to sex and brain structure.

We expanded upon the discovered sex differences in ADRD risk by highlighting a female-specific constellation of brain–behaviour associations with cardiovascular traits. As the neuroprotective effect of estrogen weakens with older age, women become more vulnerable to neurovascular disorders that can ultimately lead to dementia [57]. Cardiovascular risk factors that are exacerbated in females following menopause, such as trunk fat mass, have been associated with chronic neuroinflammation and microstructural alteration of the fornix [58,59]—the main output tract from the HC that carries direct neural signals toward partner regions of the midline DN [60]. Building on existing literature, we identified ADRD-related divergences in the fimbria of the fornix in healthy participants for mode 8 that we have linked to selective brain–behaviour associations with proxies of cardiovascular health (e.g., water mass, fat-free mass, and weight). For the same HC-DN signature, we found a female-specific association with trunk fat mass, a correlate of estrogen declines [61]. This observation supports a link between cardiovascular health, female sex, and microstructural alteration of the fornix. Despite the protective effect of *APOE* ɛ2 against ADRD previously discussed, carrying an ɛ2 allele has been associated with elevated risks for cardio- and neurovascular disorders [62–66]. *APOE* ɛ2 is indeed limited in its ability to mediate the vascular clearance of cholesterol metabolites and triglycerides that could in turn precipitate the risks of cholesterol pathologies such as hyperlipoproteinemia and cardiovascular sequelae [67]. The variability of the protective effect of physical activity on dementia risk when stratifying participants by ɛ4 status might be taken to suggest that *APOE* ɛ2 is driving the relationship between physical activity and cognitive performance [68–72]. Hypothetically, engaging in physical activity could be particularly beneficial to older female ɛ2 carriers in counteracting the rising risk of neurovascular complications resulting from the combined effect of *APOE* ɛ2 and decreased estrogen levels. Bringing support for this claim, we have shown specific interactions between HC-DN signatures and *APOE* ɛ2/2 genotype in explaining variation in physical activity—an effect that we found exclusive to females. The specificity of this effect to ɛ2 homozygotes is consistent with previous findings that have associated ɛ2 with increased longevity in centenarians [73]. Given that almost 90% of centenarians are females, the sex-specificity of our results is consistent with a genotype-driven behaviour that favours longevity via exercise in female ɛ2 homozygotes.

Epidemiological studies have provided evidence that traffic-related air pollution and residence near major roadways are associated with decreased cognitive abilities [74–82] and a higher risk of developing dementia [83–92]. The detrimental effect of air pollution on cognition could even be exacerbated in *APOE* ɛ4 carriers [82,85]. Our phenome-wide assay tied mode 1 expressions to blood markers (e.g., erythrocytes, haemoglobin, and haematocrit) and air pollution. This phenome-wide profiling supports an interplay between environmental stressors, vascular integrity, and dementia. Mode 1 also showed 19 DN hits in the PFC—a subregion in which vascular and perivascular white matter damage has been specifically observed in humans and canines chronically exposed to high levels of air pollutants [93]. Accumulation of nanoscale particulate matter in endothelium cells, basement membranes, axons, and dendrites coincided with prefrontal white matter damage, which is in line with deficits in the blood–brain barrier [93]. Such pollution-driven prefrontal white matter damage is thought to be intensified in *APOE* ɛ4 carriers [94]. Autopsy samples from patients with Alzheimer’s disease have further shown reduced pericyte coverage in CA1 and PFC (Brodmann area 9/10). These were 2 subregions in which we showed ADRD-related structural divergences in mode 1, as compared to healthy blood vessels in controls [95]. Evidence suggests that alteration of pericytes in cortex and hippocampus subregions could be modulated by ɛ4 carriership [96]. We have thus identified subregions that are consistent with early vascular leakage in the aging brain, such as CA1 and PFC, as manifesting ADRD-related structural deviation in the same HC-DN signature associated with air pollution in our phenome-wide analysis pooled across *APOE* genotypes. In doing so, we extend the alleged role of vascular integrity in protecting the brain from environmental stressors that might precipitate ADRD onset in *APOE* ɛ4 carriers.

In a similar vein, in vitro analyses have suggested that exposure to air pollution can trigger microglial activation, which in turn can cause oxidative stress [97,98]. Pollution-triggered oxidative stress could be particularly detrimental to males as they are thought to display lower expression of antioxidant enzymes responsible for scavenging reactive oxygen species [99,100]. As a result, male mice show up to 4-fold higher rates of oxidative toxicity in astrocytes, neurons, and mitochondria compared to female mice [99,101]. Our results suggest that the association between HC-DN co-variation and air pollution is male specific, building on experimental findings primarily from rodent species. Parts of the DN are thought to be among the earliest sites of Aβ accumulation [29] and consume some of the highest oxygen levels in the entire brain [102]. As such, the DN sticks out as a hotspot for both oxidative stress and ADRD pathology. A previous study has indeed found widespread glucose hypometabolism in the DN of ADRD patients that was associated with increased levels of CSF lactate, a marker of mitochondrial damage, in the OFC and mPFC as compared to cognitively healthy controls [103]. Recent evidence suggests that Aβ_1–42_ acts on reactive oxygen species to induce glucose hypometabolism [104]. One could argue that the combined effect of air pollution and amyloid pathology could be particularly detrimental in exacerbating ADRD risk among males. In line with an effect on escalating ADRD risk, specifically in males, we have linked ADRD-related structural deviation in the OFC and mPFC with a profile of associations with environmental phenotypes for mode 3. As was the case for mode 1, these associations were more prominent in males than females. In addition to emphasising a male-specific vulnerability to neurotoxicity, our phenome-wide analysis pointed towards a female-specific resilience to pollution-mediated impairment and subsequent neuronal death. For example, our phenome-wide profile for mode 1, derived for females, did not show statistically relevant associations with air pollutants but displayed a significant correlation with IGF-1. Estrogen and IGF-1 are thought to exert synergetic, non-additive effects on neurite outgrowth and survival, presumably by acting on a single neuroendocrine pathway [105]. IGF-1 is secreted by neurons and glia and possibly acts as a neurotrophic factor regulating neuroendocrine function in the central nervous system [105]. Subcutaneous injection of IGF-1 has previously been associated with increased neurogenesis in the adult rat brain [106,107]. In mode 1, in addition to a female-specific association with IGF-1, we have shown HC hits in the granule cell layer of the DG and in CA4, which are 2 subfields in which neurogenesis has been observed in rodents [106,108,109] and primates [110]. Together with its associated divergences in HC-DN co-variation expressions, the phenome-wide profile for mode 1 shed light on a female-specific resilience towards pollution-induced impairment and subsequent neuronal death. While scarcely reported in human subjects, these sex-specific divergences in vulnerability to neurotoxicity—observed here for both modes 1 and 3—are hence in accordance with experimental findings from animal models.

Building on the knowledge that ADRD and verbal-numerical reasoning overlap in underlying genetic architecture [111], we showed significant brain–behaviour associations between ADRD risk and baseline cognitive performance on the fluid intelligence battery for top modes 1, 2, and 3. While previous investigations of fluid intelligence and ADRD in the UKB were often limited to genetic evidence [111–114], we highlighted distinct HC-DN signatures related to verbal-numerical reasoning at the population level. In doing so, we found prominent ADRD-related structural divergences in the left CA1, CA2/3, presubiculum, and fimbria, which are among the first and notorious regions to be affected by ADRD pathology [13,25–27]. Some authors have claimed that white matter disruption may trigger grey matter degradation in the HC and higher-order neocortex by activating a maladaptive neuroinflammatory response [115]. Changes in fornix microstructure have indeed been reported in individuals at risk of ADRD before the onset of clinical symptoms [26] and subsequently identified as an accurate predictor of progression from mild cognitive impairment to ADRD [27]. Consistent with the early involvement of the fornix in ADRD-associated cognitive deficits, we showed structural divergence in the fornix’s fimbria and 56 DN regions for mode 3, which were accompanied by a profile of associations with questions from the fluid intelligence battery.

Recent brain-imaging evidence has extended the concept of a hippocampally mediated cognitive map to interpersonal relationships by highlighting the involvement of the DN, and hence the fornix, in schematic representations of the self and others. Notably, fMRI results from Tavares and colleagues suggest that the HC tracks how we represent others in a social hierarchy while the PCC/PCu, key hubs of the DN, tracks the social distance between ourselves and others [116]. Consistent with a reliance on the HC-DN pathway for human-defining aspects of spatiotemporal processing, we found a brain–behaviour association with navigating family relationships, a subtest of the fluid intelligence battery, that was significant in males for mode 3. We have thus provided a plausible link between verbal-numerical reasoning and ADRD risk that was accompanied by alterations in HC and DN subregion co-variation regimes involved in episodic processing.

By exploring risk-anatomy links across the different *APOE* gene variants, we have tied social engagement measures to subject-specific expressions of HC-DN co-variation signatures. Notably, we found that the contribution of social behaviours to risk-anatomy links went beyond genetic risk and was prominent across the different *APOE* genotypes. In older age, a decrease in social activity possibly related to unemployment and/or retirement could increase feelings of loneliness and consequently escalate the risk of cognitive decline and ADRD [117]. Social disengagement has indeed been associated with the incidence of cognitive decline among older adults [118–120]. In contrast, engaging in social activities has been linked with up to a 40% decrease in ADRD risk [68,118,121]. While social support has been associated with a dampened stress response [122], loneliness is thought to affect not only neuroendocrine but also immune functions [123,124]. Volunteering and having student status, 2 social engagements that have repeatedly been flagged in our analyses, could possibly downplay the pathological stress response observed in lonely older adults. Our study has thus uncovered risk-anatomy links that are consistent with the involvement of social factors as potentially preventing or exacerbating ADRD risk.

Our clustering analyses also uncovered that neurotic behaviours show unique ties to HC-DN co-variation expressions in ɛ2 carriers. Neuroticism, which is intimately related to loneliness [125], could predispose individuals to ADRD by weakening strong social support ties and increasing chronic stress through dysregulation of the hypothalamus–pituitary–adrenal (HPA) axis [123,126]. In fact, the HC subiculum, presubiculum, and parasubiculum are believed to have direct connections to the hypothalamus via the fornix [127]. These connections could possibly provide a pathway through which the subjective appraisal of one’s relationships, which can in turn result in loneliness or neuroticism if social needs are unfulfilled, is conveyed to the HPA to affect the stress response. Prospective cohort studies have indeed linked neuroticism to higher risks of developing cognitive impairments [128] and dementia [129–131]. Yet, no effects of ɛ4 dosage on cognitive decline have been observed in neurotic individuals in these previous reports [128,130]. The absence of a relationship between *APOE* and neurotic traits reported by previous studies might arise from restricting analyses to ɛ4 carriers [128,130]. Indeed, the combined analysis of ɛ4 and the K variant of *BCHE*, another genetic risk factor associated with ADRD, revealed an intriguing association between the combined risk alleles, increased basal levels of serum glucocorticoids, cognitive performance, and lower self-esteem in older adults [132]. The ramifications of neuroticism for ADRD risk, which might be underscored by *APOE* ɛ2, have been overlooked in all these studies. Recent evidence has also shown that having a positive outlook on aging, such as a sense of purpose, amplified the protective effect of *APOE* ɛ2 against cognitive decline [133]. The protective effect of *APOE* ɛ2 on cognition was enhanced for individuals with positive beliefs about aging and reduced for those with negative beliefs to the point where ɛ2 carriers no longer showed a significant cognitive advantage [133]. Our results add elements to this literature by suggesting that having a negative outcome on life, which is characteristic of a neurotic personality type, is especially detrimental to ɛ2 carriers as reflected by unique patterns of brain–behaviours associations with specific HC and DN subregions. The opposing health effects of neuroticism and social activity are possibly reflected in the brain, as social and neurotic phenotypes were divided into 2 main groups when clustered based on their correlation with HC-DN co-variation regimes for ɛ2 homozygotes. Our study thus reinforces the detrimental effect of neuroticism on ADRD risk and characterised its unique interplay with HC-DN co-variation expressions in ɛ2 homozygotes.

In sum, the typically protective benefits conferred by *APOE* ɛ2 regarding ADRD risk have mainly been discussed in epidemiological cohorts that were not designed to incorporate inter-individual differences in high-resolution brain structure assessments. In contrast, neuroimaging investigations of healthy participants before the onset of ADRD-associated clinical symptoms have focused on characterising the functional correlates of ɛ4 carriership. Our present study has reconciled these 2 research streams by contrasting profiles of brain–behaviours associations characteristic of *APOE* ɛ2 and ɛ4 in a large epidemiological cohort of approximately 40,000 participants. In doing so, we were uniquely positioned to illuminate sex-specific associations with modifiable risk factors that were unique to ɛ2 and ɛ4 homozygotes. Key risk factors relevant to ɛ2 carriers included neuroticism, social disengagement, and physical inactivity. In contrast, environmental phenotypes that repeatedly emerged in our results as being linked to ADRD risk could be especially detrimental to ɛ4 carriers. These distinct risk factors could guide potential clinical interventions and governmental policies.

## Methods

### Population data source

The UK Biobank (UKB) is a large-scale data-collection initiative that offers in-depth information on approximately 500,000 participants recruited from across Great Britain (https://www.ukbiobank.ac.uk/). This rich epidemiological cohort comprises a wide variety of resources, including physical and cognitive assessments, as well as demographic and health records. In addition to the availability of genetic data for most participants through a genotyping array (and more recently through whole-exome sequencing), the UKB provides multimodal imaging scans that are routinely augmented and will extend to approximately 100,000 participants by the end of 2022. The present study was based on the data release from February/March 2020. To ensure reproducibility, we adopted the uniform preprocessing pipelines designed and carried out by FMRIB, Oxford University, United Kingdom [134]. Building on a uniform quality control workflow enables a better comparison to other and future UKB research. At the time of data release, expert-curated image-derived phenotypes of grey matter morphology (T1-weighted magnetic resonance imaging) were available for 38,292 participants. Grey matter phenotypes from these participants were used to compute dominant regimes of structural correspondence between the hippocampus (HC) and default network (DN) and identify anatomical subregions that systematically differentiate individuals with and without a family history of ADRD. As for all subsequent analysis steps, we focused on the 37,291 participants with both *APOE* single-nucleotide polymorphisms (SNPs) genotyping (rs429358 and rs7412) and brain-imaging measures (47% men and 53% women). When recruited, these participants were aged 40 to 70 years (mean age 54.8, standard deviation [SD] 7.5 years). The demographic information for the UKB participants included in the present study, grouped per *APOE* genotypes, can be found in Table 1. The present analyses were conducted under UK Biobank application number 25163. UK Biobank participants gave written, informed consent for the study, which was approved by the Research Ethics Committee under application 11/NW/0382. Further information on the consent procedure can be found elsewhere (http://biobank.ctsu.ox.ac.uk/crystal/field.cgi?id=200).

**Table 1:** UK Biobank demographic information.

	ɛ3/3	ɛ3/4	ɛ2/3	ɛ2/4	ɛ4/4	ɛ2/2
N (%)	22,129 (59.3)	8,613 (23.1)	4,625 (12.4)	885 (2.4)	822 (2.2)	217 (0.6)
Age, Mean ± SD	54.9 ± 7.5	54.5 ± 7.4	55.0 ± 7.5	55.0 ± 7.5	54.3 ± 7.3	54.6 ± 7.5
Sex, n (%)						
Females	11,579 (52.3)	4,634 (53.8)	2,464 (53.3)	489 (55.3)	447 (54.4)	117 (53.9)
Males	10,550 (47.7)	3,979 (46.2)	2,161 (46.7)	396 (44.7)	375 (45.6)	100 (46.1)
Family history of ADRD, n (%)						
Maternal	3,516 (15.9)	1,972 (22.9)	695 (15.0)	204 (23.1)	227 (27.6)	27 (12.4)
Paternal	1,871 (8.5)	1,078 (12.5)	382 (8.3)	100 (11.3)	136 (16.5)	18 (8.3)
Both	328 (1.5)	235 (2.7)	77 (1.7)	20 (2.3)	39 (4.7)	2 (0.9)
Household income, n (%)						
Less than 18,000 £	2,786 (12.6)	1,077 (12.5)	570 (12.3)	110 (12.4)	103 (12.5)	24 (11.1)
18,000 to 30,999 £	4,980 (22.5)	1,851 (21.5)	1,067 (23.1)	206 (23.3)	168 (20.4)	43 (19.8)
31,000 to 51,999 £	6,602 (29.8)	2,639 (30.6)	1,379 (29.8)	262 (29.6)	245 (29.8)	72 (33.2)
52,000 to 100,000 £	6,086 (27.5)	2,413 (28.0)	1,314 (28.4)	238 (26.9)	240 (29.2)	63 (29.0)
Greater than 100,000 £	1,675 (7.5)	633 (7.3)	278 (6.4)	69 (7.7)	66 (8.0)	15 (6.9)
Age completed full-time education, Mean ± SD	17.0 ± 2.4	17.0 ± 2.4	17.0 ± 2.4	16.9 ± 2.4	16.8 ± 2.5	16.9 ± 2.0
Fluid intelligence score, Mean ± SD	6.2 ± 2.2	6.2 ± 2.1	6.2 ± 2.2	6.3 ± 2.3	6.2 ± 2.2	6.1 ± 2.2

Distribution of the demographic information from the UK Biobank participants included in the present study grouped per *APOE* genotypes.

ADRD, Alzheimer’s disease and related dementia; SD, standard deviation.

### Target phenotype for ADRD risk

We used the self-reported family history of ADRD as a simple but accurately measurable estimate of ADRD risk. ADRD is the terminology adopted and recommended by the National Institute on Aging, one of the US Federal Government’s National Institutes of Health, to characterise the umbrella of symptoms, diagnoses, and risk factors characteristic of Alzheimer’s disease (https://www.nia.nih.gov/health/alzheimers). The acronym “ADRD” acknowledges the known heterogeneity of clinical diagnoses of dementia. Additionally, one can only ultimately confirm Alzheimer’s disease at the highest degree of certainty based on post-mortem tissue analysis. In the UKB resource, maternal (UKB data field 20110) and paternal (UKB data field 20107) history of ADRD was ascertained as part of the initial assessment (2006 to 2010). As per UKB protocol, participants were asked, “Has/did your mother ever suffer from Alzheimer’s disease or dementia?” and “Has/did your father ever suffer from Alzheimer’s disease or dementia?”. This exact phenotype has been successfully treated as a reliable estimate of maternal/paternal history of late-onset Alzheimer’s disease by previously published genome-wide association studies conducted in the UKB cohort that successfully recovered well-known genetic risk loci for this diagnosis [135–137]. There were a total of 9,776 (25.5%) participants with self-reported parental history of ADRD within the brain-imaging cohort of 38,292 participants. Of those with family risk, 6,820 UKB participants reported an occurrence of ADRD on their mother’s side and 3,675 participants on their father’s side. A minority of participants reported both maternal and paternal history of ADRD (719 individuals).

Most genome-wide association studies have adopted a case-control framework that focused on the difference in allele frequency between patients with ADRD and healthy controls [138,139]. While useful in identifying risk loci associated with clinical diagnosis, this approach might not be best suited to derive a reliable estimate of ADRD liability in the general population. When dealing with late-onset diseases, such as ADRD, using “proxy cases,” i.e., the relatives of affected individuals, could allow for a more complete characterisation of disease risk among individuals before the onset of clinical symptoms [136]. It was a key advantage that working with proxy cases also allowed us to boost the sample size and, thus, the statistical power of our quantitative analyses to identify more suitable effects. In particular, self-report of family history of ADRD in the UKB, precisely the same phenotype at the core of the present investigation, was found to replicate established risk loci from case-control investigations as well as identify novel loci [136,137].

### Brain-imaging and preprocessing procedures

Magnetic resonance imaging (MRI) scanners (3T Siemens Skyra) were matched at several dedicated data collection sites with the same acquisition protocols and standard Siemens 32-channel radiofrequency receiver head coils. Brain-imaging data were defaced, and any sensitive meta-information was removed to protect the anonymity of the study participants. Automated processing and quality control pipelines were deployed [134,140]. Noise was removed utilising 190 sensitivity features to improve the homogeneity of the imaging data. This approach allowed for the reliable identification and exclusion of problematic brain scans, such as due to excessive head motion.

The structural MRI data were acquired as high-resolution T1-weighted images of brain anatomy using a 3D MPRAGE sequence at 1 mm isotropic resolution. Preprocessing included gradient distortion correction (GDC), field of view reduction using the Brain Extraction Tool [141] and FLIRT [142,143], as well as nonlinear registration to MNI152 standard space at 1 mm resolution using FNIRT [144]. All image transformations were estimated, combined, and applied by a single interpolation step to avoid unnecessary interpolation. Tissue-type segmentation into cerebrospinal fluid, grey matter, and white matter was applied using FAST (FMRIB’s Automated Segmentation Tool, [145]) to generate full bias-field-corrected images. In turn, SIENAX [146] was used to derive volumetric measures normalised for head sizes.

Parcellation of the DN was anatomically guided by the Schaefer-Yeo reference atlas [147]. We extracted a total of 400 parcels among the 7 canonical networks, 91 of which were defined as belonging to the DN. Volume extraction for 38 HC subregions was conducted using Freesurfer automatic sub-segmentation [21], which drew on an ultra-high-resolution (approximately 0.1 mm isotropic) probabilistic atlas. As part of the Freesurfer 7.0 suite, HC sub-segmentation was refined by carefully considering surrounding anatomical structures.

As a preliminary procedure, these MRI-derived measures were cleaned to remove inter-individual variation in brain region volumes that could be explained by nuisance variables. Building on previous UK Biobank research [148,149], we regressed out the following variables of no interest from each brain-derived volume measure: body mass index, head size, head motion during task-related brain scans, head motion during task-unrelated brain scans, head position and receiver coil in the scanner (x, y, and z), position of scanner table, as well as the data acquisition site, in addition to age, age^2^, sex, sex*age, and sex*age^2^. Sex was acquired from the National Health Service (NHS) central registry and updated by the participant if incorrect (UKB data field 31). The nuisance-cleaned volumetric measures served as the basis of our primary co-decomposition analysis—seeking to quantify how the 91 DN subregions co-deviate with the 38 HC subregions in the context of ADRD risk.

### Population co-variation between hippocampus subregions and default-network subregions

At the heart of our analysis workflow, we derived dominant regimes of structural correspondence that provide insights into how structural variation among the finely segregated HC can track structural variation among the finely segregated DN. We employed canonical correlation analysis (CCA), a doubly multivariate statistical technique, to identify population “signatures” of HC-DN co-variation. CCA was a natural choice of method as it is specially designed to disentangle patterns of joint correlation between 2 high-dimensional variable sets [23,150,151]. The first variable set, *X*, was constructed from subject-level grey matter volume in DN subregions (number of participants × 91 DN parcels matrix). The second variable set, *Y*, was constructed from HC subregion volumes (number of participants × 38 HC parcels matrix). The 2 variable sets can be formally described as follows:

$$X\in R^{n\times p}$$

$$Y\in R^{n\times q},$$

where *n* denotes the number of observations or UKB participants, *p* is the number of DN subregions, and *q* is the number of HC subregions. Subregion volumes from both variable sets were z-scored across participants to zero mean (i.e., centering) and unit variance (i.e., rescaling). CCA then addressed the problem of maximising the linear correlation between low-rank projections from 2 variable sets or data matrices [23]. The 2 sets of linear combinations of the original variables are obtained by optimising the following target function:

$$L_{X}=XV\;L_{Y}=YU$$

$$l_{X,l}=Xv_{l}\;l_{Y,l}=Yu_{l}$$

$$corr(l_{X,l},l_{Y,l})\propto l_{X,l}^{T}l_{Y,l}=max,$$

where *V* and *U* denote the respective contributions of *X* and *Y*, *L*_*X*_ and *L*_*Y*_ denote the respective latent “modes” expression of joint variation (i.e., canonical variates) based on patterns derived from *X* and patterns derived from *Y*, *l*_*X*,*l*_ is the *l*th column of *L*_*X*_, and *l*_*Y*,*l*_ is the *l*th column of *L*_*Y*_.

Our CCA application thus sought to identify linear combinations of *X* and *Y* that optimise their low-rank projections in the derived latent embedding. Such an approach resulted in pairs of latent vectors with subject-specific expressions *l*_*X*,*l*_ and *l*_*Y*,*l*_ (i.e., canonical variates) with maximised joint correlation. Corresponding pairs of latent vectors were found by iteratively decomposing the data matrices *X* and *Y* into *k* components, where *k* denotes the number of modes given the model specification. In other words, CCA searched for the canonical vectors *u* and *v* that maximise the symmetric relationship between the data matrices of DN subregion volumes (*X*) and HC subregion volumes (*Y*). In doing so, CCA identified the 2 concomitant projections *Xv*_*l*_ and *Yu*_*l*_ that optimised the correspondence between structural variation in the segregated DN and HC.

Put differently, each principled signature of HC-DN co-variation, or mode, represents the cross-correlation between a constellation of within-DN volumetric variation and a constellation of within-HC volumetric variation that co-occurred in conjunction with each other. The set of *k* modes are mutually uncorrelated by construction (orthogonality) [23]. They are also naturally rank-ordered based on the amount of variance explained between the embedded allocortical and neocortical volume sets [23]. The first and strongest mode, therefore, explained the largest fraction of joint variation between (linear) combinations of HC subregions and (linear) combinations of DN subregions. Each ensuing cross-correlation signature captured a fraction of structural variation that is not explained by one of the *k*−1 other modes. The Pearson’s correlation between a pair of canonical variates (i.e., canonical correlation) is commonly used to quantify the linear correspondence between HC subregions and DN subregions for a given mode. The 2 variable sets were entered into CCA after a confound-removal procedure based on previous UK Biobank research (cf. above).

### Group difference analysis

After constructing population signatures of conjoint HC-DN co-variation, we performed a rigorous group difference analysis to single out microstructural divergences in specific anatomical subregions with respect to ADRD family history. For each of the derived modes of HC-DN co-variation, we aimed to isolate anatomical subregions that show statistically defensible deviation in individuals with and without a family history of ADRD. To do so, we carried out a principled test that assessed any statistically relevant differences in the solution vector obtained from the CCA (i.e., canonical vectors, cf. above) of individuals at ADRD risk compared to the control group without ADRD family history (cf. above for target phenotype).

Following previous UK Biobank research [24,152], we robustly characterised the difference between individuals with and without a family history of ADRD by carrying out a bootstrap difference test of the CCA solution at hand [153]. This approach aimed to identify consistent patterns of deviation that differentiate subjects with and without a family history of ADRD. We first proceeded by constructing several alternative datasets that we could have gotten (with the same sample size), which capture the underlying population variation. For each of the 100 bootstrap iterations, these alternative datasets were built by randomly pulling participant samples with replacements. In each such bootstrap iteration, we estimated 2 CCA models in parallel by fitting 1 separate model to each of the 2 groups. In doing so, we carried out 2 * 100 separate model estimations of the doubly multivariate correspondence between HC subregions and DN subregions.

To compare the CCA solution in individuals with and without a family history of ADRD, we matched corresponding modes based on sign invariance and mode rank order. Canonical vectors of a given mode that carried opposite signs were aligned by multiplying 1 with −1. The importance rank of the CCA modes was adjusted by sorting Pearson’s correlation coefficients between pairs of corresponding canonical vectors (i.e., canonical correlations) from strongest to weakest. To estimate a quantity of group difference in relation to ADRD risk, we performed the elementwise subtraction of the corresponding canonical vector entries of a given mode *k* between the 2 groups. Pooling outcomes across the 100 bootstrap iterations, we thus aggregated the difference estimate for each canonical vector entry, thereby quantifying the uncertainty deviation for each particular HC or DN subregion.

By probing the underlying population variation, we were able to quantify the degree of uncertainty within each of our derived modes of HC-DN co-variation. For each identified population signature, we therefore isolated statistically defensible group differences in microanatomically defined HC and DN subregions. ADRD-related structural divergences were determined by whether the two-sided confidence interval included zero or not according to the 10/90% bootstrap-derived distribution of difference estimates [149]. In doing so, we obtained a nonparametric estimate of how ADRD risk is manifested in specific subregions for each of the 25 examined HC-DN signatures.

### SNP genotyping: 6 variants of *APOE* gene

We capitalised on our large sample size to demystify the HC-DN co-variation expressions associated with ɛ2 allele and ɛ4 allele homozygotes compared to their heterozygous counterparts for the ɛ2, ɛ3, or ɛ4 alleles. Genotype-level sampling and quality control procedures for the UKB are available online (https://biobank.ndph.ox.ac.uk/showcase/label.cgi?id=263). *APOE* genotypes were determined based on 2 SNPs: rs429358 and rs7412. *APOE* ɛ4 was determined as the combination of rs429358(C) and rs7412(C). *APOE* ɛ2 was determined as the combination of rs429358(T) and rs7412(T). *APOE* ɛ3 was determined based on rs429358(T) and rs7412(C). A total of 37,291 participants had both *APOE* genotyping and brain-imaging-derived measures. Among those participants, 9,525 (25.5%) reported a family history of ADRD. We observed 6 different *APOE* gene variants in our population sample: ɛ3/3 (59.3%), ɛ3/4 (23.1%), ɛ2/3 (12.4%), ɛ2/4 (2.4%), ɛ4/4 (2.2%), and ɛ2/2 (0.6%), which correspond to frequencies expected from a population primarily composed of people from European decent [22]. Contrasting the effect of ɛ2 versus ɛ4 allele dosage on inter-individual expressions of HC-DN co-variation enabled us to quantify the degree to which distinct *APOE* allelic combinations are characteristic of ADRD risk (cf. next section). In doing so, we aimed to interrogate gradual dosage effects in brain-*APOE* associations rather than simply look at ɛ4 carrier versus non-carrier status.

### Phenome-wide analysis of brain–behaviour associations in relation to ɛ2 versus ɛ4 dosage

We performed a rich annotation of the HC-DN co-variation signatures by means of their phenome-wide association with UKB traits. We were interested in how ɛ2 versus ɛ4 allele dosage is manifested in inter-individual expressions of HC-DN co-variation and how these manifestations, in turn, relate to UKB traits among a variety of predefined risk categories. We benefited from a rich portfolio of phenotypes encompassing lifestyle, cognitive, mental, and physical health assessments to ascribe profiles of brain–behaviour associations to each of the 25 modes of HC-DN co-variation.

We started with a raw collection of approximately 15,000 phenotypes that we fed into the FMRIB UKB Normalisation, Parsing And Cleaning Kit (FUNPACK version 2.5.0; https://zenodo.org/record/4762700#.YQrpui2caJ8). FUNPACK was used to extract phenotype information covering 11 major categories, including cognitive and physiological assessments, physical and mental health records, blood assays, as well as sociodemographic and lifestyle factors. We removed any brain-imaging-derived information. The diet category was additionally excluded from downstream analyses as it contained only 4 phenotypes. FUNPACK was designed to perform automatic refinement on the UKB data, which included removing “do not know” responses and filling the blank left by unanswered sub-questions. For example, the amount of alcohol drunk on a typical drinking day for a participant who indicated not drinking would be scored as zero drinks, even though this sub-question was not actually asked at assessments. FUNPACK’s output consisted of a collection of 3,330 curated phenotypes which were then fed into PHEnome Scan ANalysis Tool (PHESANT [154], https://github.com/MRCIEU/PHESANT) for further refinement. In addition to data cleaning and normalisation, PHESANT categorised the data as belonging to 1 of 4 datatypes: categorical ordered, categorical unordered, binary, and numerical. Categorical unordered variables were one-hot encoded, such that each possible response was represented by a binary column (true or false). The final curated inventory comprised 977 phenotypes spanning 11 FUNPACK-defined categories.

We next checked for statistically robust associations between HC-DN signatures and the portfolio of 977 extracted phenotypes with respect to ADRD genetic risk. We used a one-step stacking strategy [155,156] to predict genetic risk as a function of individual expressions of HC-DN co-variation. Data stacking consists of using a “base” model, often linear regression [156], to express an input vector in a lower-dimensional space. The output of the base model, which often consists of a single variable, can then be used as a single predictor in a new “stacking” model. Therefore, data stacking addressed the problem of selecting a single best predictor out of a combination of highly correlating input variables—which in our case were the corresponding HC and DN co-variation patterns. Such an approach allowed us to re-express a whole signature of HC-DN co-variation in terms of the degree it tracked the associated risk conferred by *APOE*. We formed a single continuous number representing how much a given HC-DN signature reflects ɛ2 versus ɛ4 dosage for a given individual. Investigations of *APOE* ɛ4 dosage effects have been prevalent in brain imaging research [114,157,158].

The Alzheimer’s disease research community has widely endorsed encoding ɛ4 dosage in a stepwise fashion, i.e., based on the number of allele copies carried by a given patient [112,114,157,158]. By adopting such target variable representation, Lyall and colleagues have found a significant interaction between *APOE* genotype dosage and coronary artery disease in estimating verbal-numerical scores from the fluid intelligence battery in the UK Biobank [112]. Lyall and colleagues, however, missed looking at ɛ2 dosage despite the well-established association between the ɛ2 allele and neurovascular diseases [62,63]. More recently, *APOE* ɛ4 dosage, stepwise encoded as 0, 1, or 2, was shown to be significantly associated with right hippocampal volume and white matter intensity in the UK Biobank [114]. The authors, however, did not benefit from investigating HC anatomical segmentations besides the standard head/body/tail subdivision [114]. Again, *APOE* ε2 dosage was not considered in this previous work even though neuroimaging evidence has lent support for a dose-dependent increase in hippocampal volume of 769.3 mm3 per copy of the ε2 allele, on average [158].

Consequently, the present study builds on the widely shared belief that the ε2 and ε4 alleles have largely opposing effects on Alzheimer’s risk and pathophysiology [38,159,160]. We sought an analogous composite dosage scale that readily captures opposite effects in modelling the HC and DN volume variation dependent on the copy number of ε2 and ε4 alleles. We thus created a bivariate dosage scale by summing up positive “ɛ2” and negative “ɛ4” alleles, such that a homozygous individual carrying *APOE* ɛ2/2 would have a score of +2 and one carrying *APOE* ɛ4/4 a score of −2. The neutral *APOE* ɛ3 allele, usually considered as a baseline risk in epidemiological studies [22], was scored as 0. Using a bivariate dosage scale made it possible to investigate the antagonistic effects of ɛ2 and ɛ4 in a single model. In doing so, we stayed faithful to our overarching goal of unravelling their adversarial impact on HC-DN co-variation.

Aiming to capture possible sex-specific effects, we regressed the ɛ2 versus ɛ4 dosage on inter-individual expressions of a given mode in males and females separately. We thus estimated 2 * 25 different base models, 1 for each HC-DN signature and each sex, that each had 2 parameters: the pair of co-variation expressions (i.e., canonical vectors, cf. above) associated with the HC and DN patterns. We used these 25 regression models to explain the subject-level ɛ2 versus ɛ4 dosage as a function of HC-DN co-variation expressions. For each subject and mode combination, we asked what would the expected ɛ2 versus ɛ4 dosage be given this subject’s specific expression of HC-DN co-variation? For each subject, we hence used the regression model to explain a range from −2 to +2 for each mode, which represented the ɛ2 versus ɛ4 dosage associated with their individual expression of HC-DN co-variation. For each mode, we selected the 5th and top 95th percentiles to identify the top 5% and lower 5% of individuals who were more versus less likely to develop ADRD based on the derived ɛ2 versus ɛ4 dosage risk. We focused on the extreme of the dosage distribution to target the brain-*APOE* associations especially linked to ɛ2 and ɛ4. The analogous approach is widely adopted in genome-wide analyses to remove associations not directly linked to the target genotype [161,162].

For each sex separately and for a given mode, the designated participants were put to a test of association with the 977 curated UKB phenotypes, with appropriate correction for multiple comparisons. The Pearson’s correlation between a phenotype and genetic risk predicted based on a specific HC-DN signature revealed both the association strength and accompanying statistical significance of the given mode-trait association. For each HC-DN signature, 2 widely used procedures were carried out to adjust for the multitude of associations being assessed. First, we adjusted for the number of tested phenotypes by using Bonferroni’s correction for multiple comparisons (0.05/977 = 5.11 × 10^−5^). Second, we used the FDR, another popular adjustment, although less stringent than Bonferroni’s correction. The FDR [163] was set as 5% [140,164,165] and computed for each HC-DN signature in accordance with standard practice [166]. For the sake of visualisation, we used Miami plots to compare the profiles of brain–behaviour associations derived from males and females. For visualisation purposes, phenotypes in Miami plots were coloured and grouped according to the category membership defined by FUNPACK.

### Clustering of risk factors based on their correlation with HC-DN co-variation expressions

We next systematically explored nonlinear associations between established ADRD risk phenotypes and HC-DN co-variation expressions across the different *APOE* gene variants. Our goal was to probe for clusters of risk factors that are interrelated with the derived patterns of HC and DN co-variation. To this end, we used a hierarchical clustering approach that allowed us to assess the relative importance of ensuing clusters in each of the different *APOE* genotypes to explore gradual *APOE* dosage effects on risk-anatomy links.

We adopted a targeted approach by focusing on a set of 63 risk factors (collection of phenotypes used previously [34]), including classical cardiovascular and demographic traits, as well as social richness indicators recently linked to ADRD in the UKB cohort. The first step of the clustering analysis consisted of multiplying the z-scored canonical variates by each of the 6 one-hot encoded *APOE* genotypes (i.e., ɛ2/2, ɛ2/3, ɛ3/3, ɛ2/4, ɛ3/4, and ɛ4/4) such that participants without a given genotype were zeroed out. The 6 ensuing matrices (number of participants × 50 canonical variates) represented the individual expressions of HC-DN co-variation signatures for participants with a given *APOE* genotype, whereas other participants were scored as 0s. We then computed Spearman’s correlation between these 6 genotype-specific matrices and the z-scored risk factor matrix (37,291 participants × 63 risk factors) to investigate risk-anatomy links. Spearman’s correlation is a nonparametric measure of statistical dependence between the rankings of 2 variables that can be used to capture monotonic nonlinear phenomena. The Spearman’s correlation coefficients reduce to the Pearson’s correlation between the rank values of 2 variables and hence range from −1 (inversely proportional association) to +1 (proportional association). We obtained a new cross-association matrix *X*∈R^63 x 50^ which represented the Spearman’s correlation between the 63 risk factors and the 50 canonical variates for each of the 6 *APOE* genotypes. The obtained Spearman’s correlation coefficients thus carried the nonlinear association strength of a given risk-anatomy link for a particular *APOE* genotype.

For each of the 6 *APOE* genotypes, we performed an agglomerative hierarchical clustering analysis on *X* to regroup risk factors based on their 50 associations with HC-DN co-variation pattern expressions. We used Ward’s minimum variance method [167] to compute the linkage matrix between the Spearman’s correlation coefficients of each risk-anatomy link in Euclidian space. Ward’s minimum variance criterion consists in minimising the total within-cluster variance defined as the error sum of squares:

$$d_{ij}=d(\left\{X_{i}\},\{X_{j}\right\})=||{X_{i}-X_{j}||}^{2},$$

where *d*_*ij*_ represents the squared Euclidean distance between 2 points (or cluster of points) *i* and *j*. At each step, the pair of coefficients or preceding candidate clusters that give the minimum increase in within-cluster variance is selected for merging. The procedure was performed recursively until all coefficients were merged into a single cluster. For each of the 6 *APOE* genotypes, we could thus create a dendrogram that represented the distance in Euclidian space between the clusters retained after 3 levels of branching. The level of branching refers to the number of divisions from the final merge. The dendrograms allowed us to visualise the clustering results for each of the 6 *APOE* genotypes at the same level of branching and identify meaningful clusters of risk-anatomy links that are shared or unique. To provide a more direct assessment of the degree of dissimilarity, we have compared the spread between nodes in the analogous dendrograms for each *APOE* genotype. We used Pearson’s correlation to examine the Euclidean distance between the 2 descendent links across corresponding hierarchical merging steps in the 6 genotype-specific cluster models.

### Regression of ADRD risk on HC-DN signatures and *APOE* gene variants

We next tested whether specific *APOE* genotypes showed interaction effects with signatures of HC-DN co-variation in explaining inter-individual differences in ADRD risk. As our goal was to highlight previously overlooked sex effects, we conducted our interaction analyses in males and females separately. In doing so, we aimed to characterise brain-*APOE* interactions in relation to their sex-specific impact on ADRD risk.

A first series of analyses consisted in regressing each of the previously investigated ADRD risk factors on *APOE* genotypes, co-variation patterns from the HC and DN sides (i.e., canonical variates), and the interaction between *APOE* genotypes and co-variation patterns, controlling for age. Aiming to capture possible sex-specific effects, we conducted separate analyses on males and females. We, therefore, looked at 61 ADRD risk factors, while age was used as a covariate and sex was the grouping factor for stratification. Each of the 25 modes of HC-DN co-variation was represented by 2 regression models: 1 for its HC pattern and 1 for its DN pattern. We thus formed 50 univariate regression models, in males and females, for each of the 61 risk factors. In each of these models, a given risk factor was regressed on 1 HC or DN canonical variate, the 6 *APOE* genotypes (ɛ2/2, ɛ2/3, ɛ3/3, ɛ2/4, ɛ3/4, and ɛ4/4), and 6 interaction terms capturing the nonlinear association between each of the 6 *APOE* genotypes and the given HC or DN pattern, controlling for age. Each regression model thus aimed at explaining variance in one of the 61 risk factors for a given sex based on these 14 parameters.

As a conjoint analysis across the regression models, we performed a rigorous permutation analysis to assess the robustness of each of the 14 regression coefficients. In as many as 61,000 iterations (i.e., 61 risk factors * 1,000 iterations), we randomly shuffled the outcome variable (i.e., a given risk) across participants. We recomputed the otherwise identical regression model based on the data with randomised outcomes. We recorded the regression coefficients from each of the 61,000 iterations and used them to build empirical null distributions on which we performed two-tail statistical tests. We considered statistically relevant coefficients that differ from their respective null distributions in at least 95% of the iterations, which ensured that we were at least 5% certain that the effect was robustly different from zero. This threshold remains arbitrary as our post hoc interaction analyses were merely descriptive and designed to provide a coarse portrait of gene–brain interactions rather than claiming statistical significance. For that reason, we have made publicly available masked permutations plots at the 0.0001, 0.001, 0.01, 0.05, 0.1, 0.2, 0.5, 0.8, 0.95 percentiles for the coefficient estimates of each regression model for males (https://github.com/dblabs-mcgill-mila/HCDMNCOV_AD/tree/master/fig_5/permutation_analysis/males/masked_plots) and females (https://github.com/dblabs-mcgill-mila/HCDMNCOV_AD/tree/master/fig_5/permutation_analysis/females/masked_plots).

A second series of analyses consisted in regressing the family history of ADRD on a set of explanatory input variables including (i) *APOE* genotypes; (ii) co-variation patterns from the HC and DN sides (i.e., canonical variates); and (iii) the interaction between *APOE* genotypes and co-variation patterns, controlling for age. For each sex, we built separate logistic models for each of the 25 HC and 25 DN canonical variates, for a total of 50 models per sex. In each model, the family history of ADRD (encoded as 0 for no and 1 for yes) was regressed on 1 HC or DN canonical variate, the 6 *APOE* genotypes (ɛ2/2, ɛ2/3, ɛ3/3, ɛ2/4, ɛ3/4, and ɛ4/4), and 6 interaction terms capturing the nonlinear association between each of the 6 *APOE* genotypes and the given HC or DN pattern, controlling for age. We thus obtained a total of 100 logistic models that sought to explain variance in the family history of ADRD as a function of these 14 parameters. We performed the analogous permutation analysis (described above) to assess the robustness of each of the 14 regression coefficients derived from these 100 logistic models. We have made publicly available the permutation distributions at the 0.0001, 0.001, 0.01, 0.05, 0.1, 0.2, 0.5, 0.8, 0.95 percentiles for the coefficients of each regression model (https://github.com/dblabs-mcgill-mila/HCDMNCOV_AD/tree/master/fig_6/permutation_analyses).

### Latent factor analysis of brain–behaviour associations

To finally distill latent factor embeddings of brain–behaviour associations from our HC-DN population signatures, we used the classical linear dimensionality reduction method PCA [168]. PCA was a natural choice of method to uncover linearly independent groupings of risk factors with similar relatedness to HC-DN co-variation patterns. Latent factors uncovered by the PCA are naturally ordered from most to least important which allows us to select candidate principles of brain–behaviour association that account for the most inter-individual variance.

We started by computing the Pearson’s correlation between the z-scored canonical variate matrix (number of participants × 50 canonical variates) and the z-scored risk factor matrix (number of participants × 63 risk factors). We obtained a new matrix *M*∈R^63 x 50^, which represented the Pearson’s correlation coefficients between the 63 risk factors and the 50 canonical variates. We next decomposed *M* into latent factor groupings by using singular value decomposition (SVD). Every correlation coefficient in *M* had already been z-scored to abide by zero mean and unit variance prior to computing the SVD, as per common practice [169]. More formally, solving the SVD problem took the following form:

$$M=U\;S\;V^{T},$$

Where *U* is a 63 × 63 orthonormal matrix, *S* is a 63 × 50 diagonal matrix carrying the singular values, and *V* is a 50 × 50 orthonormal matrix carrying singular vectors.

We retained the top 3 singular vectors and expressed our correlation matrix in terms of the dot product *US*∈*R*^63 *x* 3^ to be able to represent the latent-factor projections of *M* onto the new 3D latent space. In doing so, we obtained the distinct expression levels of the 63 risk factors for each of the top 3 brain–behaviour association axes (i.e., principal component expressions). These 3 axes are by construction orthonormal and rank-ordered, representing an uncorrelated partition of the overall variance in brain–behaviour association. The leading axis captured the largest fraction of variance and was, therefore, the most explanatory, as reflected by its associated singular value.

We then conducted an acid test of the robustness of the PCA solution by performing a rigorous split-half reliability assessment across 1,000 bootstrap iterations. At each iteration, we drew 37,291 participants with replacements to simulate random participant samples that we could have pulled from the same population. We then derived 2 random subsets of equal size (*N* = 18,645) from the original sample and re-computed the Pearson’s correlation matrix *M* for each random subset separately. SVD was then performed on both matrices in parallel according to the procedure described above. We retained the same number of top 3 singular vectors and expressed each correlation matrix in terms of its projection onto its corresponding latent space. In doing so, we were able to compare the expression levels of each risk factor along the 3 main axes of brain–behaviour associations derived from each random subset. If the PCA solution is robust, similar groups of risk factors should be emphasised along corresponding dimensions, which, in turn, should explain similar fractions of the total variance. We also provided a more formal assessment of statistical agreement between both random subsets by computing the Pearson’s correlation between the weights of the 3 first principal components for random subsets 1 and 2 across the 1,000 iterations. Higher Pearson’s correlations are indicative of a substantial degree of agreement between both subsets, which in turn attests to the robustness of the original PCA solution.

Based on the desire to audit our cohort analysis for sex-specific associations, we computed the Pearson’s correlation matrix *M* in males and females separately and repeated the PCA procedure described above for each group. Once more, we retained the top 3 singular vectors and expressed the correlation matrices in terms of their projection onto their corresponding latent embedding. We compared the expression levels of the risk factors along corresponding latent dimensions to highlight sex-specific brain–behaviour associations. In the absence of major sex differences, similar groups of risk factors should be emphasised along analogous dimensions, which should correspondingly explain similar fractions of the total variance.

We performed a similar bootstrap analysis of the sex-specific PCA solutions to formally assess the robustness of our findings. Across 1,000 bootstrap iterations, we drew 17,561 males and 19,730 females with replacements to simulate random participant samples that we could have gotten from the original population. At each iteration, re-computed the Pearson’s correlation matrix for each random subset separately and repeated the analogous SVD decomposition. As for the split-half reliability assessment, we Pearson’s correlated the weights of the 3 first principal components for male- and female-derived solutions in each of the 1,000 iterations. Lower Pearson’s correlations would suggest a higher degree of sex-specificity in the PCA solutions.

### External validation

Using the openly available PREVENT-AD (PResymptomatic EValuation of Experimental or Novel Treatments for Alzheimer’s disease (AD); [170]) cohort, we have performed a rigorous test of the external validation for our HC-DN co-variations signatures derived from the UKB cohort. The PREVENT-AD cohort is composed of older individuals with a known family history of Alzheimer’s disease that were cognitively unimpaired at the time of enrollment from 2011 to 2017 (mean age 63, SD 5 years) [170]. Participants of the PREVENT-AD initiative have undergone extensive annual health and cognitive assessments for up to 5 years. This resource creates a unique opportunity to monitor longitudinal trajectories of brain-imaging assessments, cerebral fluid biochemistry, neurosensory capacities, and medical charts in pre-symptomatic individuals at Alzheimer’s risk. Our independent PREVENT-AD sample consisted of 386 participants (27% men, 73% women) with the following APOE genotype distribution: ɛ3/3 (51.2%), ɛ3/4 (33.1%), ɛ2/3 (10.5%), ɛ2/4 (3.0%), ɛ4/4 (2.1%). Further information on the PREVENT-AD cohort and access to the open data inventory can be found online (https://prevent-alzheimer.net).

The PREVENT-AD resources provide structural brain-imaging scanning (T1-weighted images of brain anatomy) for up to 4 years of follow-up for 362 participants, totaling 980 participant assessment visits. For the brain-imaging data from each participant visit, we first performed a full FreeSurfer reconstruction followed by subcortical volumetric sub-segmentation of the 38 hippocampal subfields, analogous to the UKB brain-imaging preprocessing pipeline. We next parsed the structural brain scans according to the Schaefer-Yeo parcellation (400 parcels, 7 networks) to obtain the analogous 91 parcels defined as belonging to the DN (https://github.com/ThomasYeoLab/CBIG/tree/master/stable_projects/brain_parcellation/Schaefer2018_LocalGlobal/Parcellations/project_to_individual). Age, age2, sex, sex*age, and sex*age2 were regressed out from each brain-derived grey matter volume measure as part of the deconfounding procedure. The final brain-imaging sample consisted of 344 participants with a total of 916 individual visits (64 visits were excluded based on errors in the preprocessing pipeline). Of the remaining visits, 117 came from participants without *APOE* SNP genotyping and were hence excluded.

In so doing, we extracted the same collection of brain-image-derived phenotypes of grey matter morphology as in the UKB. We were thus in a position to compute the expression of the 25 UKB-derived modes of HC-DN co-variation based on grey matter measurements for the 91 DN and 38 HC subregions in PREVENT-AD participants. For each visit, we obtained 25 pairs of subject-specific expressions of each of the 25 brain signatures of HC-DN structural co-variation (i.e., canonical variates), which served as a basis for our external validation analyses in unseen subjects.

Across MRI visits, we tested whether 25 different signatures of HC-DN co-variations are associated with different subsets among the rich palette of PREVENT-AD phenotypes designed to track ADRD progression in pre-symptomatic individuals.

To do so, using Pearson’s correlation, we computed the association strength between the individual expressions of the 25 modes of HC-DN co-variation and 157 PREVENT-AD phenotypes that spanned CSF and blood samples, comprehensive cognitive and functional assessments, as well as demographic and health records. To assess the robustness of the correlation coefficients, we randomly permuted the PREVENT-AD phenotypes across participants in 1,000 iterations and recomputed the Pearson’s correlation coefficients. Recording the results from these 1,000 iterations, we built an empirical null distribution for each correlation coefficient. We reported only the coefficients that were robustly different from their respective empirical null distributions in at least 95% of the 1,000 permutation iterations.

## Supporting information

S1 Fig ADRD-related divergences in HC and DN subregions for mode 2 and the associated phenome-wide profile. Shown here are ADRD-related subregion divergences for mode 2 for the HC (leftmost panel) and DN (central panel). We identified 10 HC hits, most of them located in the left hemisphere. The strongest HC divergences were observed for the presubiculum, hippocampal fissure, and CA2/3. We found corresponding DN hits in posterior midline structure (posterior cingulate cortex and restrosplenial cortex), the dorsomedial prefrontal cortex, and the posterior and temporal cortices. In males and females separately, we regressed *APOE* dosage on HC and DN co-variation patterns from mode 2. We then used these sex-specific models to predict *APOE* dosage based on inter-individual expressions of mode 2. The right panel displays the Miami plot for the correlations between *APOE* scores in the context of mode 2 and the portfolio of UKB phenotypes for males (upper half) and females (lower half). We found significant associations with the fluid intelligence battery that were unique to males. Data underlying this figure can be found at https://github.com/dblabs-mcgill-mila/HCDMNCOV_AD/tree/master/Miami_Plots (DOI: 10.5281/zenodo.7126809). ADRD, Alzheimer’s disease and related dementia; CA, cornu amonis; DG, granule cell layer of the dentate gyrus; dmPFC, dorsomedial prefrontal cortex; DN, default network; FDR, false discovery rate correction; HC, hippocampus; IPL, inferior parietal lobule; MTS, middle temporal sulcus; PrS, presubiculum; PCC, posterior cingulate cortex; RSC, retrosplenial cortex; STS, superior temporal sulcus.
(TIFF)

S2 Fig ADRD-related divergences in HC and DN subregions for mode 6 and the associated phenome-wide profile. Shown here are ADRD-related subregion divergences for mode 6 for the HC (leftmost panel) and DN (central panel). We identified 1 HC hit to the hippocampus–amygdala transition area with no concurrent DN divergences. In males and females separately, we regressed *APOE* dosage on HC and DN co-variation patterns from mode 6. We then used these sex-specific models to predict *APOE* dosage based on inter-individual expressions of mode 6. The right panel displays the Miami plot for the correlations between *APOE* scores in the context of mode 6 and the portfolio of UKB phenotypes for males (upper half) and females (lower half). We found significant associations with physical phenotypes and blood assays that were unique to females. Data underlying this figure can be found at https://github.com/dblabs-mcgill-mila/HCDMNCOV_AD/tree/master/Miami_Plots (DOI: 10.5281/zenodo.7126809). ADRD, Alzheimer’s disease and related dementia; DN, default network; FDR, false discovery rate correction; HATA, hippocampus–amygdala transition area; HC, hippocampus; IMT, intima-medial thickness.
(TIFF)

S3 Fig ADRD-related divergences in HC and DN subregions for mode 10 and the associated phenome-wide profile. Shown here are ADRD-related subregion divergences for mode 10 for the HC (leftmost panel) and DN (central panel). We identified 1 HC hit to the hippocampus–amygdala transition area with no concurrent DN divergences. In males and females separately, we regressed *APOE* dosage on HC and DN co-variation patterns from mode 10. We then used these sex-specific models to predict *APOE* dosage based on inter-individual expressions of mode 10. The right panel displays the Miami plot for the correlations between *APOE* scores in the context of mode 10 and the portfolio of UKB phenotypes for males (upper half) and females (lower half). We found 1 significant association with sitting height unique to males. Data underlying this figure can be found at https://github.com/dblabs-mcgill-mila/HCDMNCOV_AD/tree/master/Miami_Plots (DOI: 10.5281/zenodo.7126809). ADRD, Alzheimer’s disease and related dementia; DN, default network; FDR, false discovery rate correction; HATA, hippocampus–amygdala transition area; HC, hippocampus.
(TIFF)

S4 Fig ADRD-related divergences in HC and DN subregions for mode 4 and the associated phenome-wide profile. Shown here are ADRD-related subregion divergences for mode 4 for the HC (leftmost panel) and DN (central panel). We identified 4 DN hits to the dorsomedial prefrontal cortex with no concurrent HC divergences. In males and females separately, we regressed *APOE* dosage on HC and DN co-variation patterns from mode 4. We then used these sex-specific models to predict *APOE* dosage based on inter-individual expressions of mode 4. The right panel displays the Miami plot for the correlations between *APOE* scores in the context of mode 4 and the portfolio of UKB phenotypes for males (upper half) and females (lower half). We found 1 significant association with receiving an attendance, disability or mobility allowance that was unique to females. Data underlying this figure can be found at https://github.com/dblabs-mcgill-mila/HCDMNCOV_AD/tree/master/Miami_Plots (DOI: 10.5281/zenodo.7126809). ADRD, Alzheimer’s disease and related dementia; dmPFC, dorsomedial prefrontal cortex; DN, default network; FDR, false discovery rate correction; HC, hippocampus.
(TIFF)

S5 Fig ADRD-related divergences in HC and DN subregions for mode 7 and the associated phenome-wide profile. Shown here are ADRD-related subregion divergences for mode 7 for the HC (leftmost panel) and DN (central panel). We identified 9 DN hits to the frontal lobe with no concurrent HC divergences. In males and females separately, we regressed *APOE* dosage on HC and DN co-variation patterns from mode 7. We then used these sex-specific models to predict *APOE* dosage based on inter-individual expressions of mode 7. The right panel displays the Miami plot for the correlations between *APOE* scores in the context of mode 7 and the portfolio of UKB phenotypes for males (upper half) and females (lower half). We found 1 significant association with diastolic blood pressure that was unique to females. Data underlying this figure can be found at https://github.com/dblabs-mcgill-mila/HCDMNCOV_AD/tree/master/Miami_Plots (DOI: 10.5281/zenodo.7126809). ADRD, Alzheimer’s disease and related dementia; dmPFC, dorsomedial prefrontal cortex; DN, default network; FDR, false discovery rate correction; HC, hippocampus; OFC, orbitofrontal cortex.
(TIFF)

S6 Fig ADRD-related divergences in HC and DN subregions for mode 11 and the associated phenome-wide profile. Shown here are ADRD-related subregion divergences for mode 11 for the HC (leftmost panel) and DN (central panel). We identified 1 DN hit to the posterior cingulate cortex with no concurrent HC divergences. In males and females separately, we regressed *APOE* dosage on HC and DN co-variation patterns from mode 11. We then used these sex-specific models to predict *APOE* dosage based on inter-individual expressions of mode 11. The right panel displays the Miami plot for the correlations between *APOE* scores in the context of mode 11 and the portfolio of UKB phenotypes for males (upper half) and females (lower half). We found 1 significant association with the standing height that was unique to females. Data underlying this figure can be found at https://github.com/dblabs-mcgill-mila/HCDMNCOV_AD/tree/master/Miami_Plots (DOI: 10.5281/zenodo.7126809). ADRD, Alzheimer’s disease and related dementia; DN, default network; FDR, false discovery rate correction; HC, hippocampus; PCC, posterior cingulate cortex.
(TIFF)

S7 Fig ADRD-related divergences in HC and DN subregions for mode 13 and the associated phenome-wide profile. Shown here are ADRD-related subregion divergences for mode 13 for the HC (leftmost panel) and DN (central panel). We identified 1 DN hit to the superior temporal sulcus with no concurrent HC divergences. In males and females separately, we regressed *APOE* dosage on HC and DN co-variation patterns from mode 13. We then used these sex-specific models to predict *APOE* dosage based on inter-individual expressions of mode 13. The right panel displays the Miami plot for the correlations between *APOE* scores in the context of mode 13 and the portfolio of UKB phenotypes for males (upper half) and females (lower half). We found significant associations with physical measurements related to height as well as feelings of guilt that were unique to females. Data underlying this figure can be found at https://github.com/dblabs-mcgill-mila/HCDMNCOV_AD/tree/master/Miami_Plots (DOI: 10.5281/zenodo.7126809). ADRD, Alzheimer’s disease and related dementia; DN, default network; FDR, false discovery rate correction; HC, hippocampus; STS, superior temporal sulcus.
(TIFF)

S8 Fig Difference in associations between males and females for the phenome-wide profiling of mode 1. Absolute difference in *p*-values for the 33 brain-phenotype associations that passed the Bonferroni correction for multiple comparisons in either males or females in the original phenome-wide profiling of mode 1. Data underlying this figure can be found at https://github.com/dblabs-mcgill-mila/HCDMNCOV_AD/tree/master/Miami_Plots (DOI: 10.5281/zenodo.7126809).
(PNG)

S9 Fig Difference in associations between males and females for the phenome-wide profiling of mode 3. Absolute difference in *p*-values for the 20 brain-phenotype associations that passed the Bonferroni correction for multiple comparisons in either males or females in the original phenome-wide profiling of mode 3. Data underlying this figure can be found at https://github.com/dblabs-mcgill-mila/HCDMNCOV_AD/tree/master/Miami_Plots (DOI: 10.5281/zenodo.7126809).
(PNG)

S10 Fig Difference in associations between males and females for the phenome-wide profiling of mode 8. Absolute difference in *p*-values for the 18 brain-phenotype associations that passed the Bonferroni correction for multiple comparisons in either males or females in the original phenome-wide profiling of mode 8. Data underlying this figure can be found at https://github.com/dblabs-mcgill-mila/HCDMNCOV_AD/tree/master/Miami_Plots (DOI: 10.5281/zenodo.7126809).
(PNG)

S11 Fig Similarity between the 6 genotype-specific clustering models. We computed Pearson’s correlation of the distance between the 2 descendent links of corresponding hierarchical merging steps among the cluster analyses for the 6 APOE genotypes (i.e., ɛ2/2, ɛ2/3, ɛ3/3, ɛ2/4, ɛ3/4, and ɛ4/4). These derived distances made it possible to formally compare the cluster nodes of analogous dendrograms for each genotype-specific cluster model. We show that ɛ2 carriers are most similar to each other, as reflected by an agglomeration of strong Pearson’s correlation coefficients in the top left corner of the heat map. The most dissimilar cluster models were ɛ2/4 and ɛ3/4, followed by ɛ2/4 and ɛ3/3, and lastly by ɛ3/3 and ɛ4/4. Data underlying this figure can be found at https://github.com/dblabs-mcgill-mila/HCDMNCOV_AD/tree/master/clustering_analysis (DOI: 10.5281/zenodo.7126809).
(TIFF)

S12 Fig Ɛ3 carriership shows risk-anatomy links with socioeconomic determinants, while ɛ2 carriership is associated with neuroticism. We multiplied the population-wide HC and DN co-variation patterns by APOE genotypes ɛ3/3 (*N* = 22,129) and ɛ2/4 (*N* = 885) such that participants who do not carry a given genotype were zeroed out. We then computed the Spearman’s correlations between these 2 new vectors and the 63 preselected Alzheimer’s disease risk factors to test for risk-anatomy links. We performed an agglomerative clustering analysis on these Spearman’s correlations, which consists in repeatedly merging Spearman’s correlations with similar variance together until all observations are merged into a single cluster. Here are shown the dendrograms, which indicate the distance between each cluster identified when retaining 3 levels of branching for APOE ɛ3/3 (leftmost panel) and ɛ2/4 (rightmost panel). We found the early branching of socioeconomic determinants ɛ3/3 (time spent watching television, education score, past and current tobacco smoking frequency, alcohol consumption on a typical drinking day, and alcohol intake frequency) in the clustering model for ɛ3/3. For ɛ2/4, we found that neuroticism-related behaviours (e.g., being worried/anxious, mood swings, and miserableness) were singled out from the other risk-anatomy links at the first branching, as was observed for other ɛ2 carriers. We thus confirm the association between ɛ3 carriership and socioeconomic determinants and between ɛ2 carriership and neurotic personality traits. Data underlying this figure can be found at https://github.com/dblabs-mcgill-mila/HCDMNCOV_AD/tree/master/clustering_analysis (DOI: 10.5281/zenodo.7126809).
(TIFF)

S13 Fig Latent factors of brain–behaviour associations emphasise satisfaction with social relationships, socioeconomic status, and neuroticism-related traits. We conducted an exploratory PCA to disentangle latent factor of brain–behaviour association in our UK Biobank sample. We first computed the Pearson’s correlations between the 25 pairs of co-variation patterns from the HC and DN sides and the 63 preselected ADRD risk factors. We then ran singular value decomposition on the risk by canonical variates matrix (X_63 × 50_) and retained the 3 first PCs that explained approximately 13.8%, approximately 9.6%, and approximately 8.2% of the total variance in the data, respectively. The upper plot displays the projections of the Pearson’s correlations onto each of the 3 main axes of brain–behaviour associations. The lower plot displays the eigenvectors for the top 10 HC and DN co-variation patterns. The first axis of brain–behaviour associations emphasises phenotypes from the social cluster previously identified on the clustering analysis of risk-anatomy links (Fig 4), e.g., attending religious group, attending adult education classes, and number of people in household. The second axis rather accented health-related phenotypes and lifestyle factors. Lastly, the third axis of brain–behaviour associations separated neuroticism-related items (being worried/anxious, being easily hurt, and worrying too long after embarrassment) from the rest of the risk factors. Data underlying this figure can be found at https://github.com/dblabs-mcgill-mila/HCDMNCOV_AD/blob/master/PCA (DOI: 10.5281/zenodo.7126809). ADRD, Alzheimer’s disease and related dementia; DN, default network; HC, hippocampus; PCA, principal component analysis.
(TIFF)

S14 Fig Reliability assessment of the principal component solution. We assessed the robustness of the derived brain–behaviour association axes by performing a split-half reliability assessment of our principal component solution across 1,000 bootstrap iterations. At each iteration, we drew 37,291 participants with replacements to simulate random participant samples that we could have pulled from the same population. We then derived 2 random subsets of equal size (*N* = 18,645) from the original sample. For each subset, we re-computed the Pearson’s correlation between all possible combinations of the 50 canonical variates and 63 target indicators. We then estimated 2 PCA models in parallel, one for each random half subset, on the z-scored correlation coefficients matrices. We show the average projections of the Pearson’s correlation coefficients on the 3 first axes of brain–behaviour associations. We found that the projections on component 1 were robust and consistent across subsets. The projections on the first axis of brain–behaviour associations accurately depicted those of the original PCA solution, with the same set of social phenotypes (e.g., attending religious group, attending adult education classes, and the number of people in the household) and socioeconomic determinants (e.g., age completed high school education, average household income, and the number of vehicles in the household) emphasised. Data underlying this figure can be found at https://github.com/dblabs-mcgill-mila/HCDMNCOV_AD/blob/master/PCA (DOI: 10.5281/zenodo.7126809).
(TIFF)

S15 Fig Statistical agreement between the PCA solutions for random subsets 1 and 2. We computed the Pearson’s correlation between the weights of the 3 first principal components for random subsets 1 and 2 across 1,000 bootstrap iterations. The weights of the first 2 components were robust, as reflected by a substantial degree of agreement between both subsets on components 1 (mean Pearson’s rho: 0.59, 90% CI: [0.38, 0.74]) and 2 (mean Pearson’s rho: 0.51, 90% CI: [0.15, 0.77]). In contrast, we showed volatility in the weights associated with component 3, as reflected by a wider and right-skewed distribution (mean Pearson’s rho: 0.25, 90% CI: [0.02, 0.56]). Data underlying this figure can be found at https://github.com/dblabs-mcgill-mila/HCDMNCOV_AD/blob/master/PCA (DOI: 10.5281/zenodo.7126809).
(TIFF)

S16 Fig Neuroticism-related items expressed distinctive brain–behaviour associations in males and females. We repeated the PCA in males (left; *N* = 17,561) and females right; *N* = 19,730) separately. In each sex, we first computed the Pearson’s correlations between the 25 pairs of co-variation patterns from the HC and DN sides and the 63 preselected ADRD risk factors. We then ran singular value decomposition on the risk by canonical variates matrix (X_63 × 50_) and retained the 3 first PCs. The PCs obtained from males had explained variance of approximately 14.6%, approximately 11.9%, and approximately 9.6%, respectively. The PCs obtained from females had explained variance of approximately 14.6%, approximately 11.9%, and approximately 7.4%, respectively. The upper plots display the projections of the Pearson’s correlations onto each of the 3 axes of brain–behaviour associations for the 2 sexes. The lower plots display the eigenvectors for the top 10 HC and DN co-variation patterns. The projections of the Pearson’s correlations onto the 2 first axes of brain–behaviour association were roughly the same in males and females. In contrast, neuroticism-related items were only emphasised on the third axis of brain–behaviour association in males. We thus supplemented our population analysis by showing that the relationship between neuroticism and patterns of HC-DN co-variation was mainly male specific. Data underlying this figure can be found at https://github.com/dblabs-mcgill-mila/HCDMNCOV_AD/blob/master/PCA (DOI: 10.5281/zenodo.7126809). ADRD, Alzheimer’s disease and related dementia; DN, default network; HC, hippocampus; PCA, principal component analysis.
(TIFF)

S17 Fig Statistical agreement between the PCA solutions for males and females. We computed the Pearson’s correlation between the weights of the first 3 principal components for the sex-specific PCA solutions across 1,000 bootstrap iterations. We observed a low agreement between the male- and female-derived PCA solutions on all 3 components, as reflected by the widespread of the distributions and small average values. Data underlying this figure can be found at https://github.com/dblabs-mcgill-mila/HCDMNCOV_AD/blob/master/PCA (DOI: 10.5281/zenodo.7126809).
(TIFF)

S18 Fig HC-DN signatures tracked different aspects of ADRD risk in independent PREVENT-AD participants. We externally validated our UKB-derived population signatures of HC-DN co-variation by investigating their mapping to ADRD-related risk factors in an unseen, independent participant sample. We tracked subject-specific expressions of the 25 modes of HC-DN co-variation in PREVENT-AD participants to a collection of 157 widely established indicators of ADRD progression. We computed the Pearson’s correlation between the HC and DN pattern expressions and the PREVENT-AD phenotypes for each mode. Only the Pearson’s correlation coefficients that were statistically different from their respective null distributions 95% of the time are present. We replicated several phenotypic associations highlighted in the UKB, such as with mode 1 and depression, mode 2 and verbal-numerical reasoning, and mode 6 and vascular integrity. We also showed that our modes of HC-DN co-variation track meaningful aspects of ADRD progression up to the 25th and last signature, for which we found associations with tau CSF levels on the HC side and cardiovascular factors (e.g., systolic blood pressure, pulse, and *APOE* ε4/4 genotype) on the DN side. We thus showed that HC-DN signatures robustly link to different aspects of ADRD risk in a completely independent cohort from the one in which the co-variation patterns have originally been derived. Data underlying this figure can be found at https://github.com/dblabs-mcgill-mila/HCDMNCOV_AD/blob/master/external_validation (DOI: 10.5281/zenodo.7126809). ADRD, Alzheimer’s disease and related dementia; DN, default network; HC, hippocampus.
(TIFF)

## Abbreviations

ADRD: Alzheimer’s disease and related dementia
*APOE*: Apolipoprotein E
CA: cornu ammonis
CCA: canonical correlation analysis
DG: dentate gyrus
dmPFC: dorsomedial prefrontal cortex
DN: default network
FDR: false discovery rate
GDC: gradient distortion correction
HC: hippocampus
HPA: hypothalamus–pituitary–adrenal
IGF-1: insulin-like growth factor 1
IPL: inferior parietal lobule
MTS: middle temporal sulcus
MoCA: Montreal Cognitive Assessment
MRI: magnetic resonance imaging
OFC: orbitofrontal cortex
PCA: principal component analysis
PCC: posterior cingulate cortex
PCu: precuneus
PREVENT-AD: PResymptomatic EValuation of Experimental or Novel Treatments for Alzheimer’s disease (AD)
RBANS: Repeatable Battery for Assessment of Neuropsychological Status
RSC: retrospenial cortex
SD: standard deviation
SNP: single-nucleotide polymorphism
STG: superior temporal gyrus
SPL: superior parietal lobule
STS: superior temporal sulcus
SVD: singular value decomposition
TPJ: temporoparietal junction
UKB: UK Biobank
vlPFC: ventrolateral prefrontal cortex
vmPFC: ventromedial prefrontal cortex
