# Supplementary figures and images for: APOE alleles are associated with sex-specific structural differences in brain regions affected in Alzheimer’s disease and related dementia
Source: PLoS Biol. 2022 Dec 13;20(12):e3001863. doi: 10.1371/journal.pbio.3001863 (PMC9747055; doi:10.1371/journal.pbio.3001863)

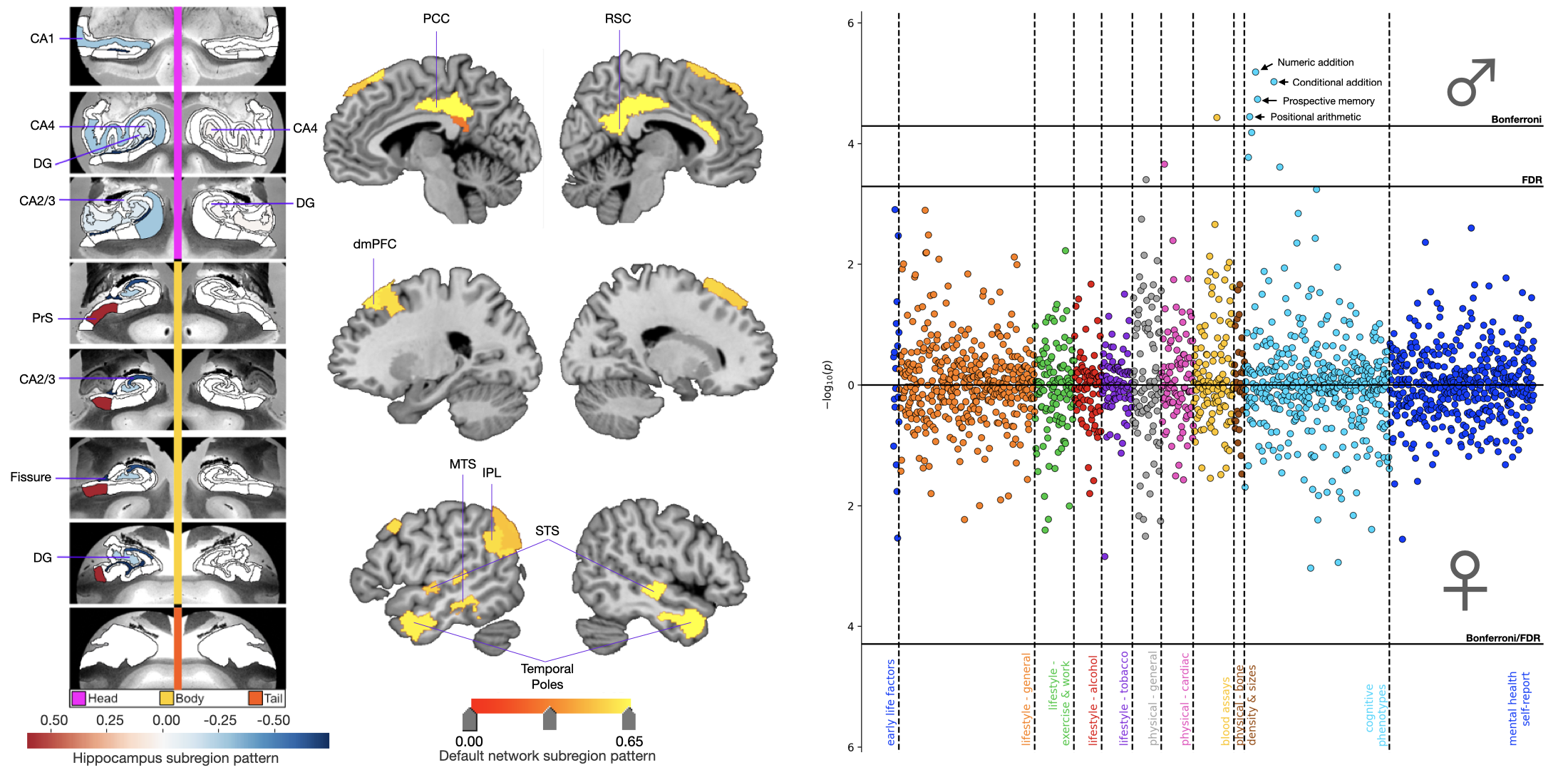

Supplement: S1 Fig — Shown here are ADRD-related subregion divergences for mode 2 for the HC (leftmost panel) and DN (central panel). We identified 10 HC hits, most of them located in the left hemisphere. The strongest HC divergences were observed for the presubiculum, hippocampal fissure, and CA2/3. We found corresponding DN hits in posterior midline structure (posterior cingulate cortex and restrosplenial cortex), the dorsomedial prefrontal cortex, and the posterior and temporal cortices. In males and females separately, we regressed APOE dosage on HC and DN co-variation patterns from mode 2. We then used these sex-specific models to predict APOE dosage based on inter-individual expressions of mode 2. The right panel displays the Miami plot for the correlations between APOE scores in the context of mode 2 and the portfolio of UKB phenotypes for males (upper half) and females (lower half). We found significant associations with the fluid intelligence battery that were unique to males. Data underlying this figure can be found at https://github.com/dblabs-mcgill-mila/HCDMNCOV_AD/tree/master/Miami_Plots (DOI: 10.5281/zenodo.7126809). ADRD, Alzheimer’s disease and related dementia; CA, cornu amonis; DG, granule cell layer of the dentate gyrus; dmPFC, dorsomedial prefrontal cortex; DN, default network; FDR, false discovery rate correction; HC, hippocampus; IPL, inferior parietal lobule; MTS, middle temporal sulcus; PrS, presubiculum; PCC, posterior cingulate cortex; RSC, retrosplenial cortex; STS, superior temporal sulcus. (TIFF) [file pbio.3001863.s001.tiff]

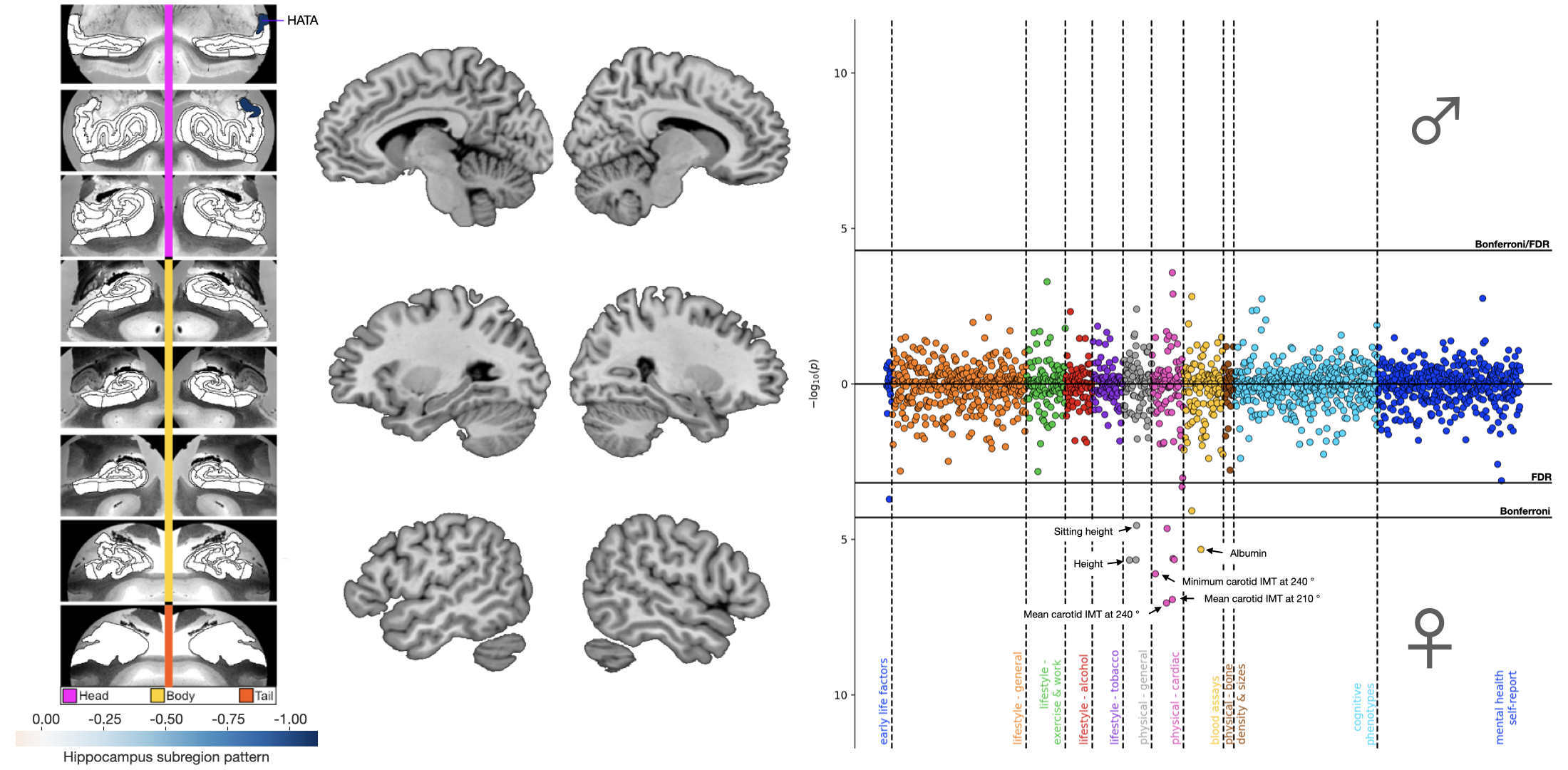

Supplement: S2 Fig — Shown here are ADRD-related subregion divergences for mode 6 for the HC (leftmost panel) and DN (central panel). We identified 1 HC hit to the hippocampus–amygdala transition area with no concurrent DN divergences. In males and females separately, we regressed APOE dosage on HC and DN co-variation patterns from mode 6. We then used these sex-specific models to predict APOE dosage based on inter-individual expressions of mode 6. The right panel displays the Miami plot for the correlations between APOE scores in the context of mode 6 and the portfolio of UKB phenotypes for males (upper half) and females (lower half). We found significant associations with physical phenotypes and blood assays that were unique to females. Data underlying this figure can be found at https://github.com/dblabs-mcgill-mila/HCDMNCOV_AD/tree/master/Miami_Plots (DOI: 10.5281/zenodo.7126809). ADRD, Alzheimer’s disease and related dementia; DN, default network; FDR, false discovery rate correction; HATA, hippocampus–amygdala transition area; HC, hippocampus; IMT, intima-medial thickness. (TIFF) [file pbio.3001863.s002.tiff]

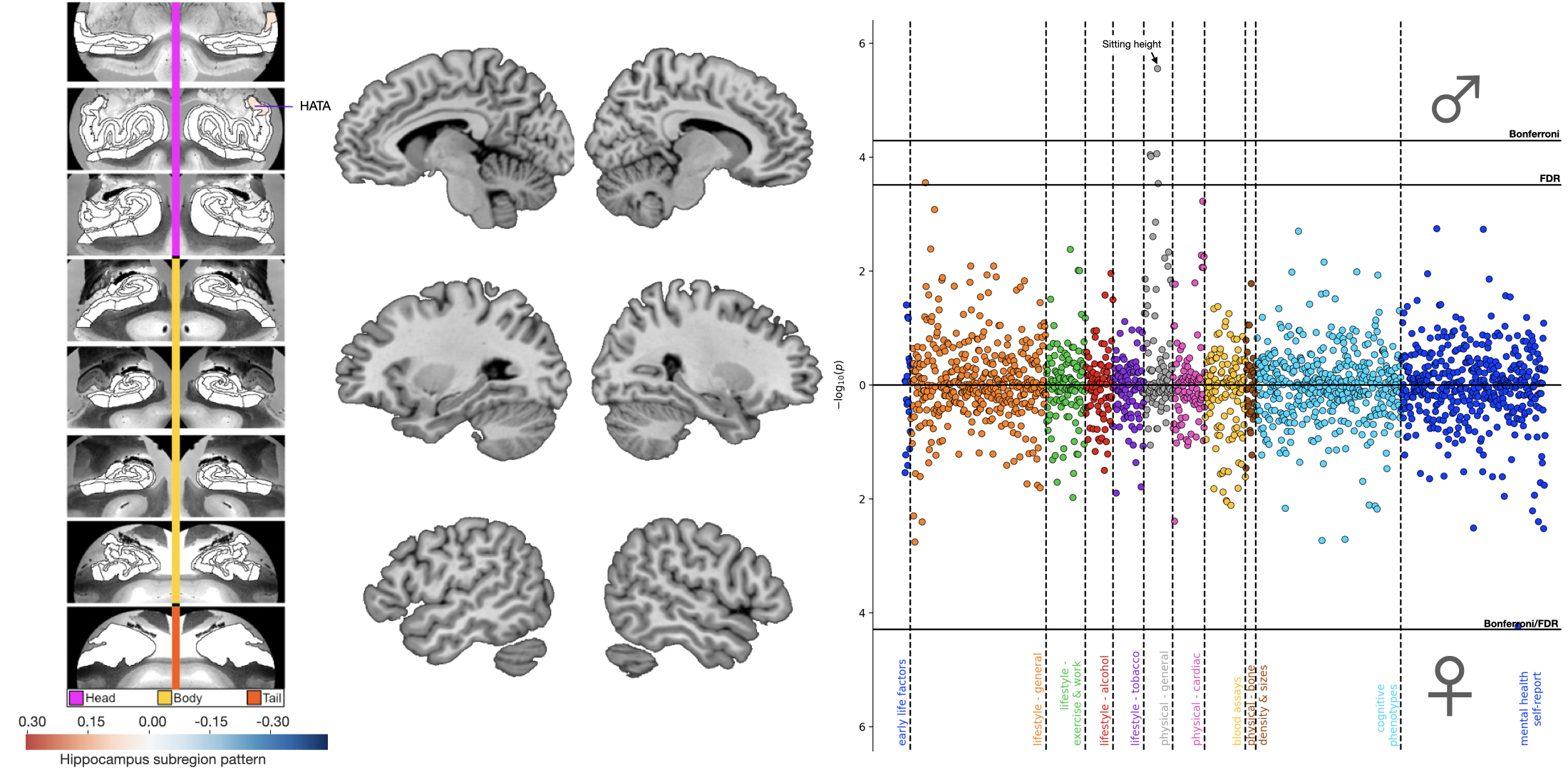

Supplement: S3 Fig — Shown here are ADRD-related subregion divergences for mode 10 for the HC (leftmost panel) and DN (central panel). We identified 1 HC hit to the hippocampus–amygdala transition area with no concurrent DN divergences. In males and females separately, we regressed APOE dosage on HC and DN co-variation patterns from mode 10. We then used these sex-specific models to predict APOE dosage based on inter-individual expressions of mode 10. The right panel displays the Miami plot for the correlations between APOE scores in the context of mode 10 and the portfolio of UKB phenotypes for males (upper half) and females (lower half). We found 1 significant association with sitting height unique to males. Data underlying this figure can be found at https://github.com/dblabs-mcgill-mila/HCDMNCOV_AD/tree/master/Miami_Plots (DOI: 10.5281/zenodo.7126809). ADRD, Alzheimer’s disease and related dementia; DN, default network; FDR, false discovery rate correction; HATA, hippocampus–amygdala transition area; HC, hippocampus. (TIFF) [file pbio.3001863.s003.tiff]

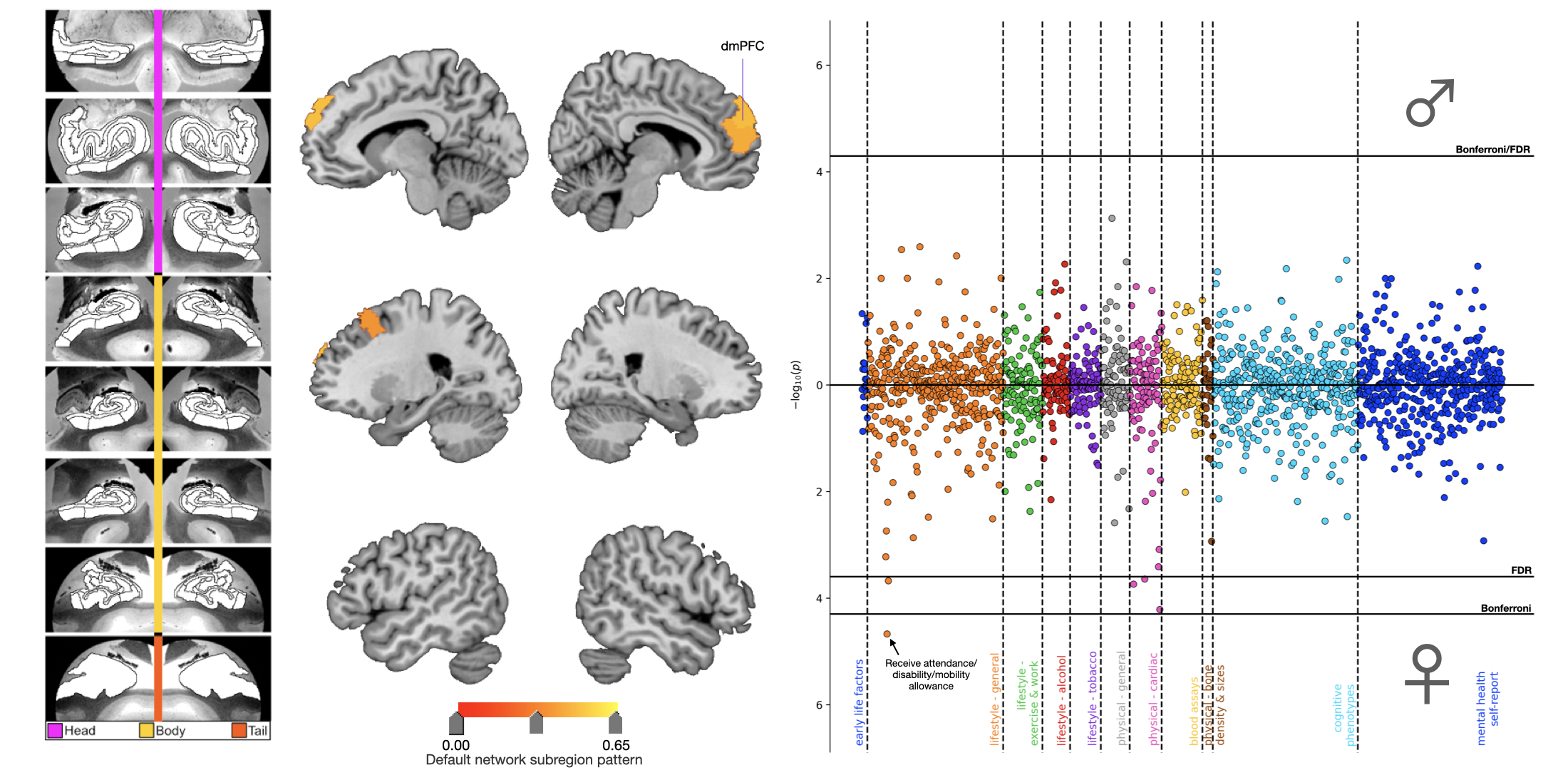

Supplement: S4 Fig — Shown here are ADRD-related subregion divergences for mode 4 for the HC (leftmost panel) and DN (central panel). We identified 4 DN hits to the dorsomedial prefrontal cortex with no concurrent HC divergences. In males and females separately, we regressed APOE dosage on HC and DN co-variation patterns from mode 4. We then used these sex-specific models to predict APOE dosage based on inter-individual expressions of mode 4. The right panel displays the Miami plot for the correlations between APOE scores in the context of mode 4 and the portfolio of UKB phenotypes for males (upper half) and females (lower half). We found 1 significant association with receiving an attendance, disability or mobility allowance that was unique to females. Data underlying this figure can be found at https://github.com/dblabs-mcgill-mila/HCDMNCOV_AD/tree/master/Miami_Plots (DOI: 10.5281/zenodo.7126809). ADRD, Alzheimer’s disease and related dementia; dmPFC, dorsomedial prefrontal cortex; DN, default network; FDR, false discovery rate correction; HC, hippocampus. (TIFF) [file pbio.3001863.s004.tiff]

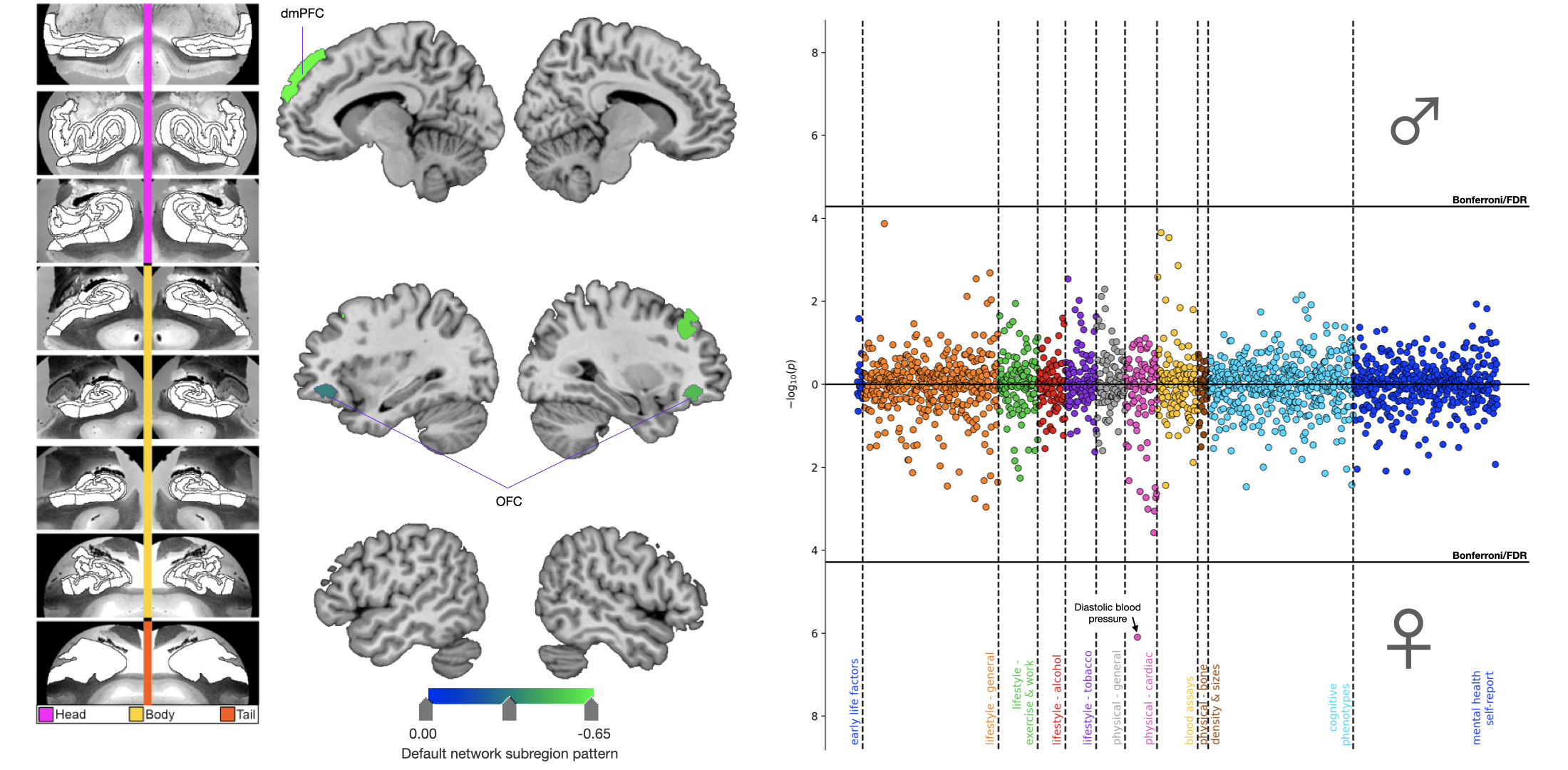

Supplement: S5 Fig — Shown here are ADRD-related subregion divergences for mode 7 for the HC (leftmost panel) and DN (central panel). We identified 9 DN hits to the frontal lobe with no concurrent HC divergences. In males and females separately, we regressed APOE dosage on HC and DN co-variation patterns from mode 7. We then used these sex-specific models to predict APOE dosage based on inter-individual expressions of mode 7. The right panel displays the Miami plot for the correlations between APOE scores in the context of mode 7 and the portfolio of UKB phenotypes for males (upper half) and females (lower half). We found 1 significant association with diastolic blood pressure that was unique to females. Data underlying this figure can be found at https://github.com/dblabs-mcgill-mila/HCDMNCOV_AD/tree/master/Miami_Plots (DOI: 10.5281/zenodo.7126809). ADRD, Alzheimer’s disease and related dementia; dmPFC, dorsomedial prefrontal cortex; DN, default network; FDR, false discovery rate correction; HC, hippocampus; OFC, orbitofrontal cortex. (TIFF) [file pbio.3001863.s005.tiff]

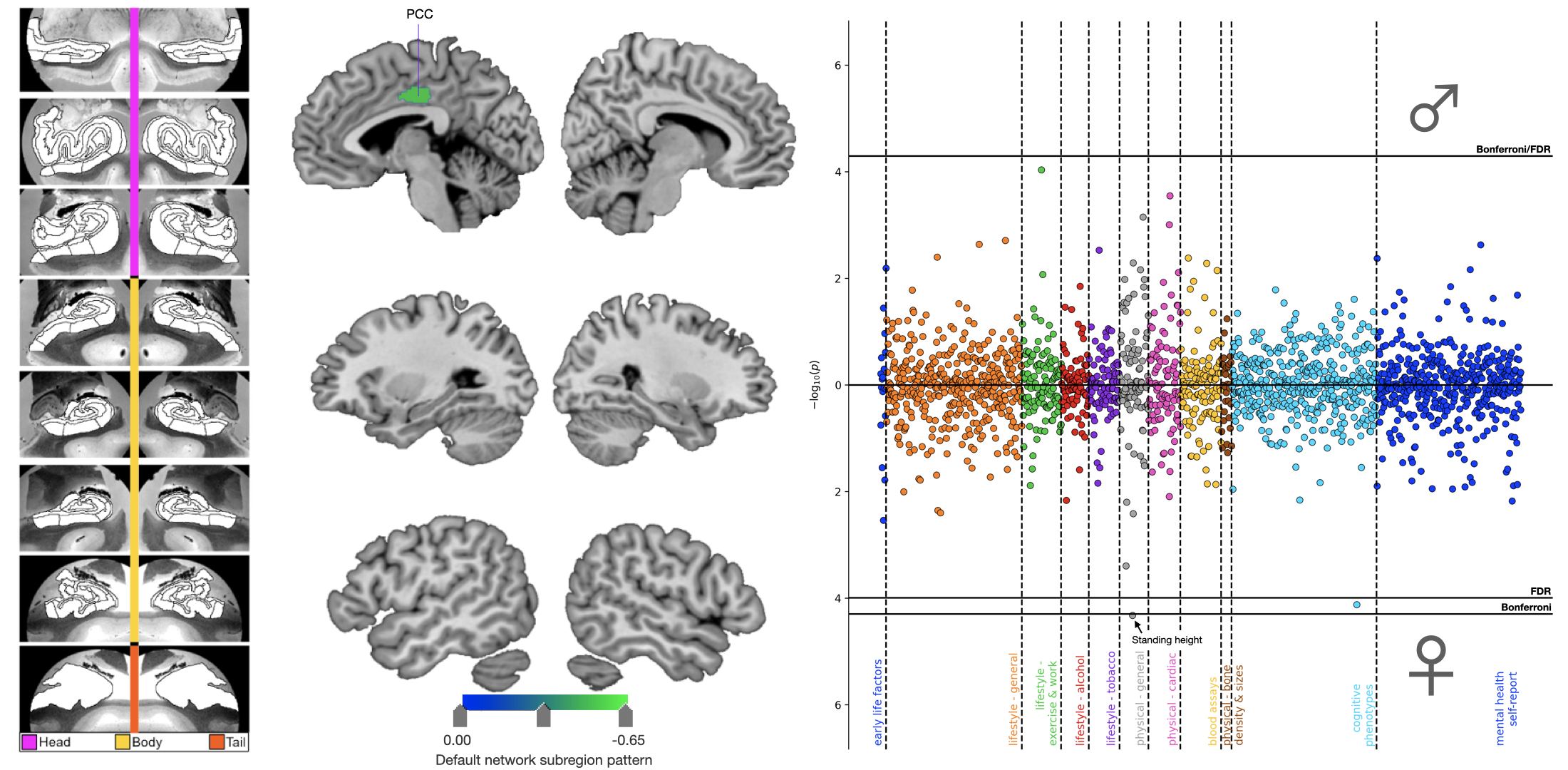

Supplement: S6 Fig — Shown here are ADRD-related subregion divergences for mode 11 for the HC (leftmost panel) and DN (central panel). We identified 1 DN hit to the posterior cingulate cortex with no concurrent HC divergences. In males and females separately, we regressed APOE dosage on HC and DN co-variation patterns from mode 11. We then used these sex-specific models to predict APOE dosage based on inter-individual expressions of mode 11. The right panel displays the Miami plot for the correlations between APOE scores in the context of mode 11 and the portfolio of UKB phenotypes for males (upper half) and females (lower half). We found 1 significant association with the standing height that was unique to females. Data underlying this figure can be found at https://github.com/dblabs-mcgill-mila/HCDMNCOV_AD/tree/master/Miami_Plots (DOI: 10.5281/zenodo.7126809). ADRD, Alzheimer’s disease and related dementia; DN, default network; FDR, false discovery rate correction; HC, hippocampus; PCC, posterior cingulate cortex. (TIFF) [file pbio.3001863.s006.tiff]

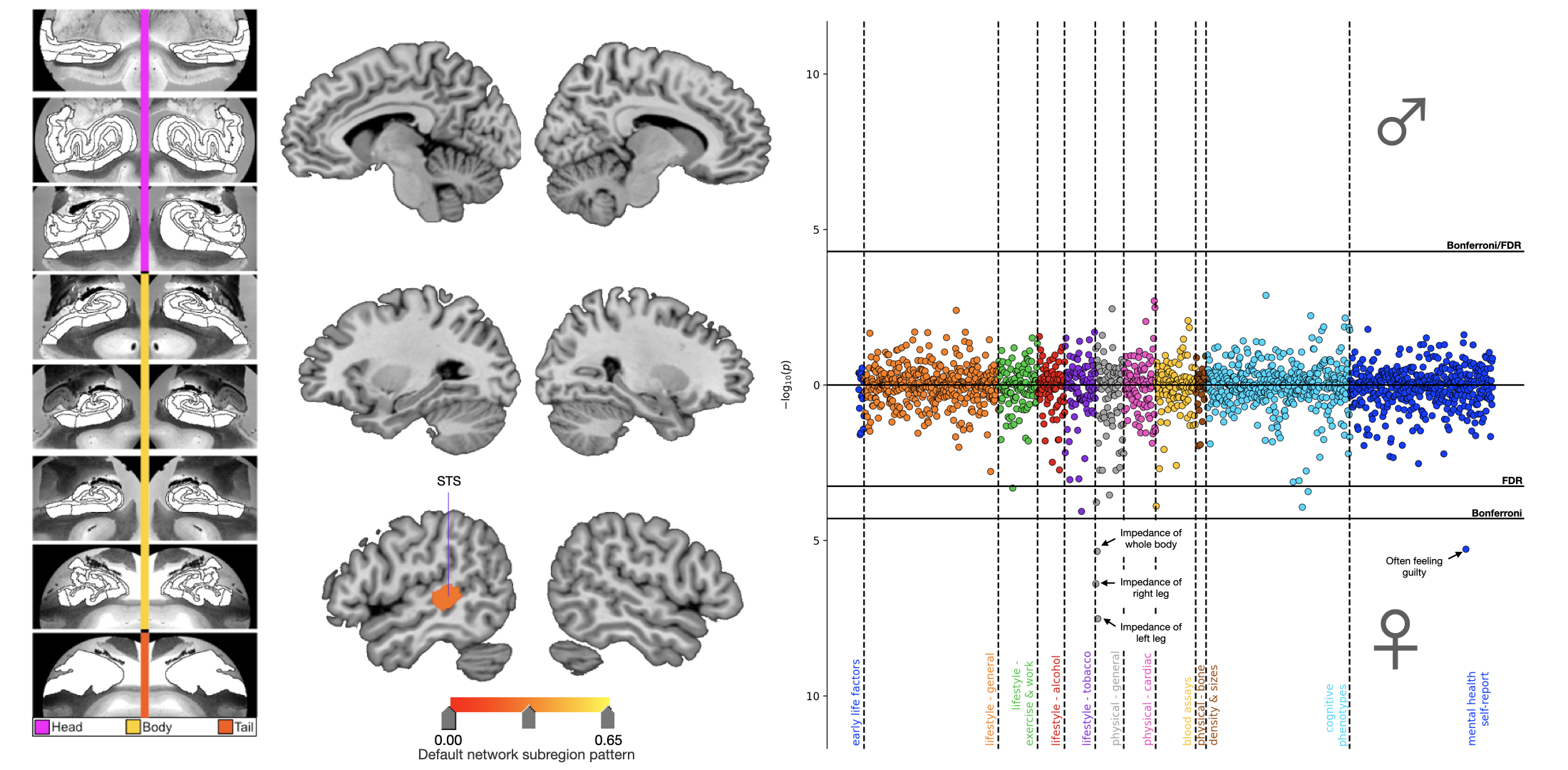

Supplement: S7 Fig — Shown here are ADRD-related subregion divergences for mode 13 for the HC (leftmost panel) and DN (central panel). We identified 1 DN hit to the superior temporal sulcus with no concurrent HC divergences. In males and females separately, we regressed APOE dosage on HC and DN co-variation patterns from mode 13. We then used these sex-specific models to predict APOE dosage based on inter-individual expressions of mode 13. The right panel displays the Miami plot for the correlations between APOE scores in the context of mode 13 and the portfolio of UKB phenotypes for males (upper half) and females (lower half). We found significant associations with physical measurements related to height as well as feelings of guilt that were unique to females. Data underlying this figure can be found at https://github.com/dblabs-mcgill-mila/HCDMNCOV_AD/tree/master/Miami_Plots (DOI: 10.5281/zenodo.7126809). ADRD, Alzheimer’s disease and related dementia; DN, default network; FDR, false discovery rate correction; HC, hippocampus; STS, superior temporal sulcus. (TIFF) [file pbio.3001863.s007.tiff]

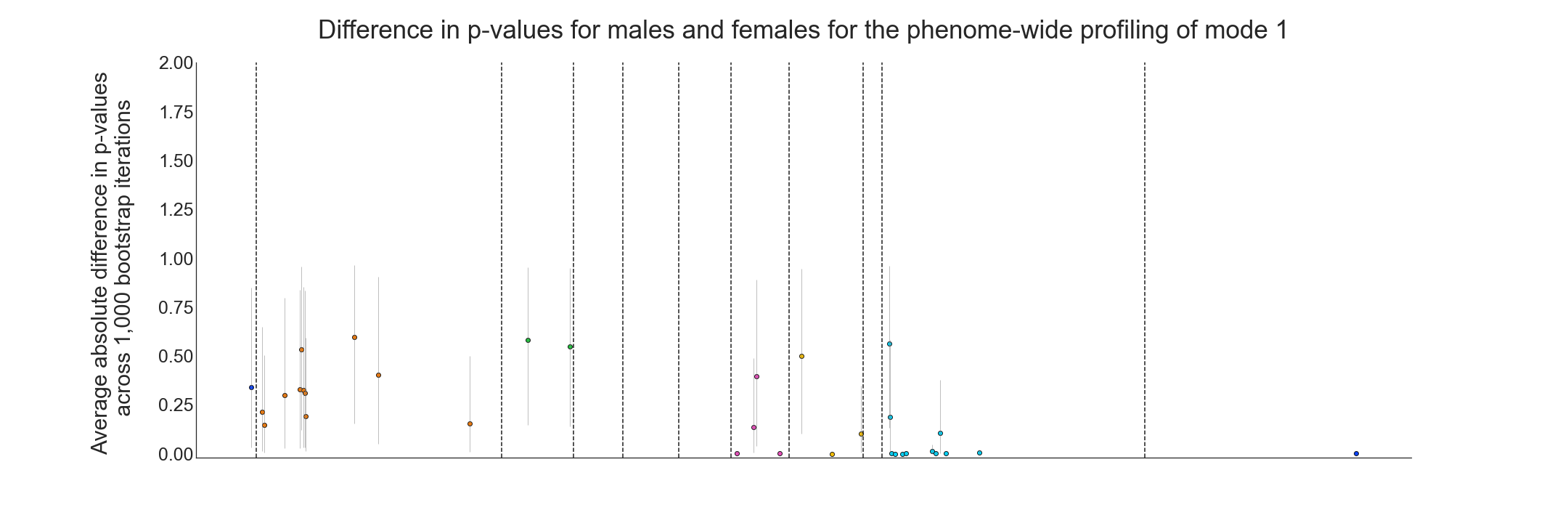

Supplement: S8 Fig — Absolute difference in p-values for the 33 brain-phenotype associations that passed the Bonferroni correction for multiple comparisons in either males or females in the original phenome-wide profiling of mode 1. Data underlying this figure can be found at https://github.com/dblabs-mcgill-mila/HCDMNCOV_AD/tree/master/Miami_Plots (DOI: 10.5281/zenodo.7126809). (PNG) [file pbio.3001863.s008.png]

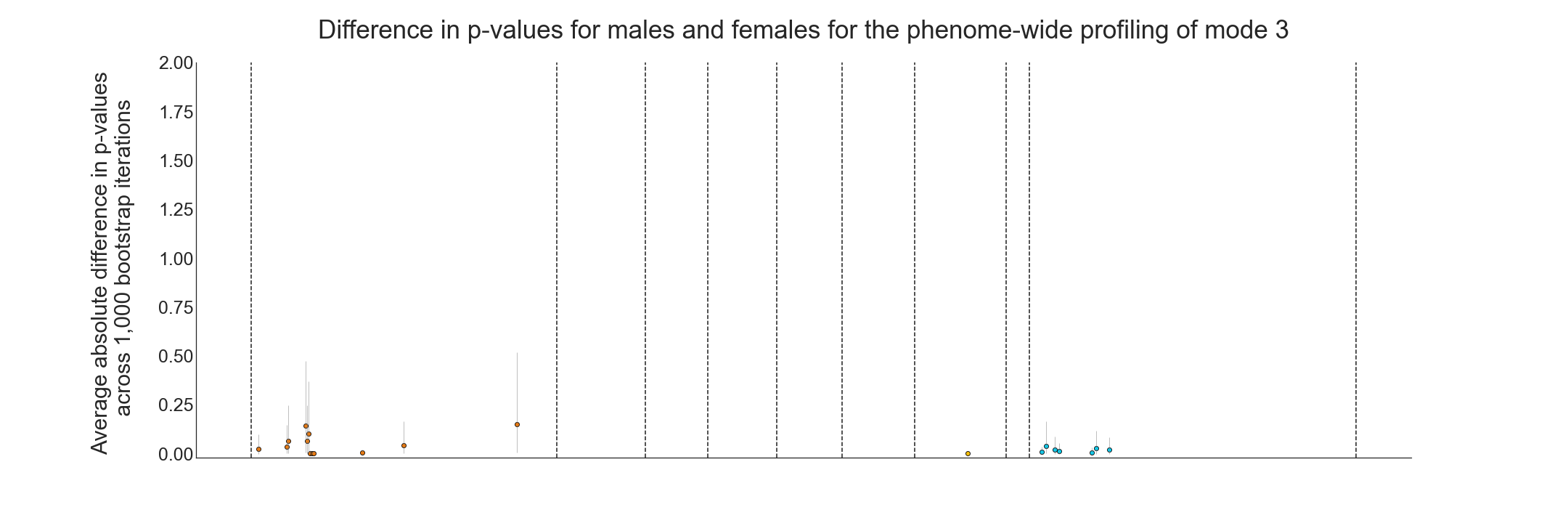

Supplement: S9 Fig — Absolute difference in p-values for the 20 brain-phenotype associations that passed the Bonferroni correction for multiple comparisons in either males or females in the original phenome-wide profiling of mode 3. Data underlying this figure can be found at https://github.com/dblabs-mcgill-mila/HCDMNCOV_AD/tree/master/Miami_Plots (DOI: 10.5281/zenodo.7126809). (PNG) [file pbio.3001863.s009.png]

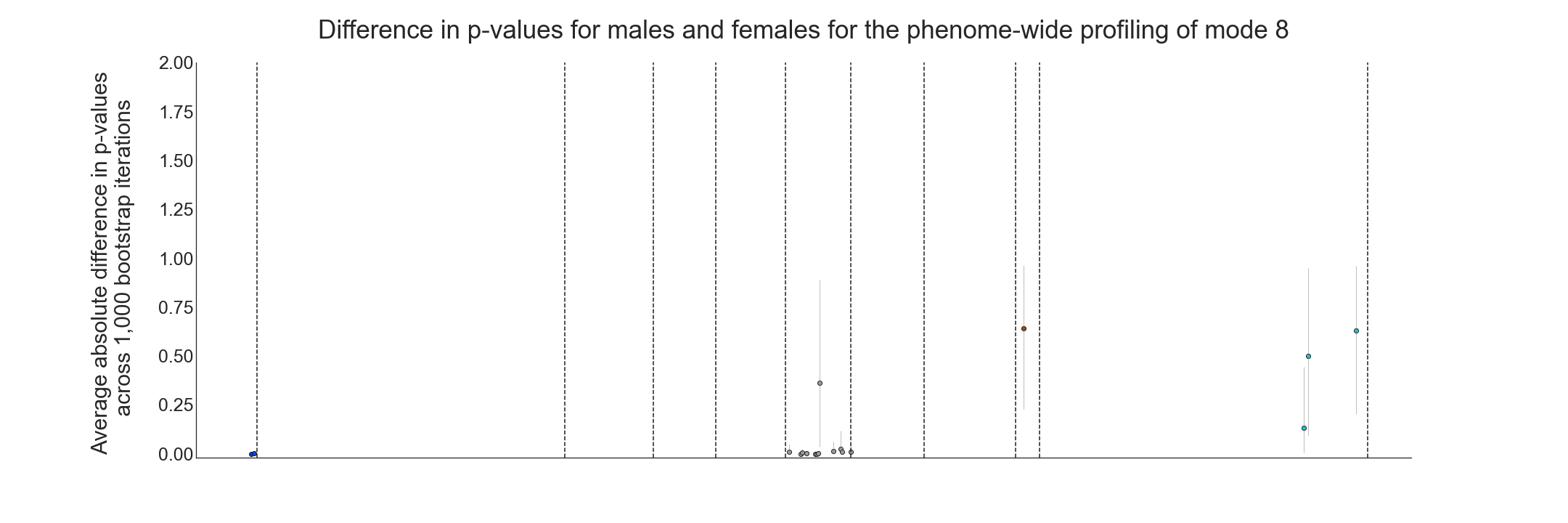

Supplement: S10 Fig — Absolute difference in p-values for the 18 brain-phenotype associations that passed the Bonferroni correction for multiple comparisons in either males or females in the original phenome-wide profiling of mode 8. Data underlying this figure can be found at https://github.com/dblabs-mcgill-mila/HCDMNCOV_AD/tree/master/Miami_Plots (DOI: 10.5281/zenodo.7126809). (PNG) [file pbio.3001863.s010.png]

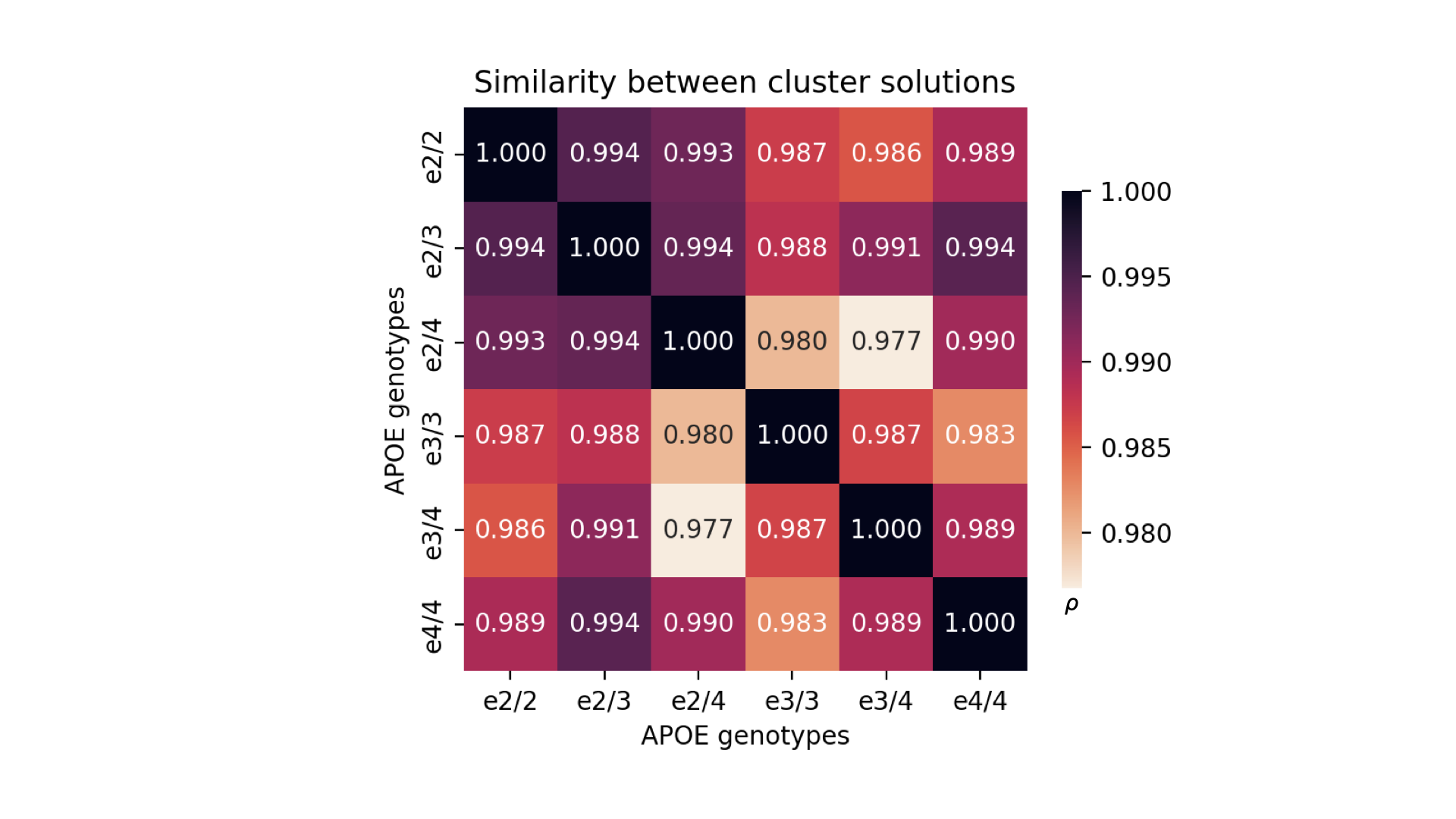

Supplement: S11 Fig — We computed Pearson’s correlation of the distance between the 2 descendent links of corresponding hierarchical merging steps among the cluster analyses for the 6 APOE genotypes (i.e., ɛ2/2, ɛ2/3, ɛ3/3, ɛ2/4, ɛ3/4, and ɛ4/4). These derived distances made it possible to formally compare the cluster nodes of analogous dendrograms for each genotype-specific cluster model. We show that ɛ2 carriers are most similar to each other, as reflected by an agglomeration of strong Pearson’s correlation coefficients in the top left corner of the heat map. The most dissimilar cluster models were ɛ2/4 and ɛ3/4, followed by ɛ2/4 and ɛ3/3, and lastly by ɛ3/3 and ɛ4/4. Data underlying this figure can be found at https://github.com/dblabs-mcgill-mila/HCDMNCOV_AD/tree/master/clustering_analysis (DOI: 10.5281/zenodo.7126809). (TIFF) [file pbio.3001863.s011.tiff]

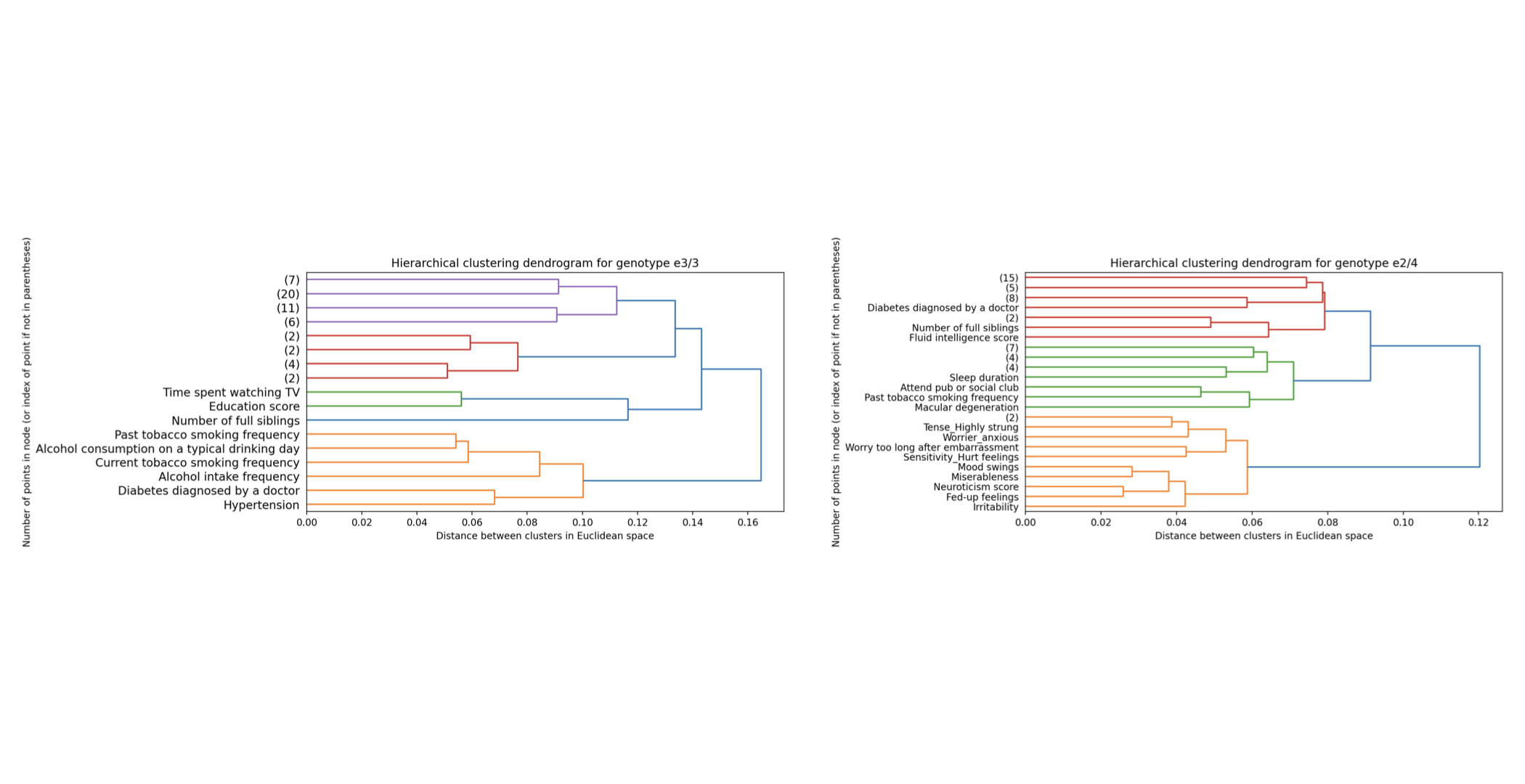

Supplement: S12 Fig — We multiplied the population-wide HC and DN co-variation patterns by APOE genotypes ɛ3/3 (N = 22,129) and ɛ2/4 (N = 885) such that participants who do not carry a given genotype were zeroed out. We then computed the Spearman’s correlations between these 2 new vectors and the 63 preselected Alzheimer’s disease risk factors to test for risk-anatomy links. We performed an agglomerative clustering analysis on these Spearman’s correlations, which consists in repeatedly merging Spearman’s correlations with similar variance together until all observations are merged into a single cluster. Here are shown the dendrograms, which indicate the distance between each cluster identified when retaining 3 levels of branching for APOE ɛ3/3 (leftmost panel) and ɛ2/4 (rightmost panel). We found the early branching of socioeconomic determinants ɛ3/3 (time spent watching television, education score, past and current tobacco smoking frequency, alcohol consumption on a typical drinking day, and alcohol intake frequency) in the clustering model for ɛ3/3. For ɛ2/4, we found that neuroticism-related behaviours (e.g., being worried/anxious, mood swings, and miserableness) were singled out from the other risk-anatomy links at the first branching, as was observed for other ɛ2 carriers. We thus confirm the association between ɛ3 carriership and socioeconomic determinants and between ɛ2 carriership and neurotic personality traits. Data underlying this figure can be found at https://github.com/dblabs-mcgill-mila/HCDMNCOV_AD/tree/master/clustering_analysis (DOI: 10.5281/zenodo.7126809). (TIFF) [file pbio.3001863.s012.tiff]

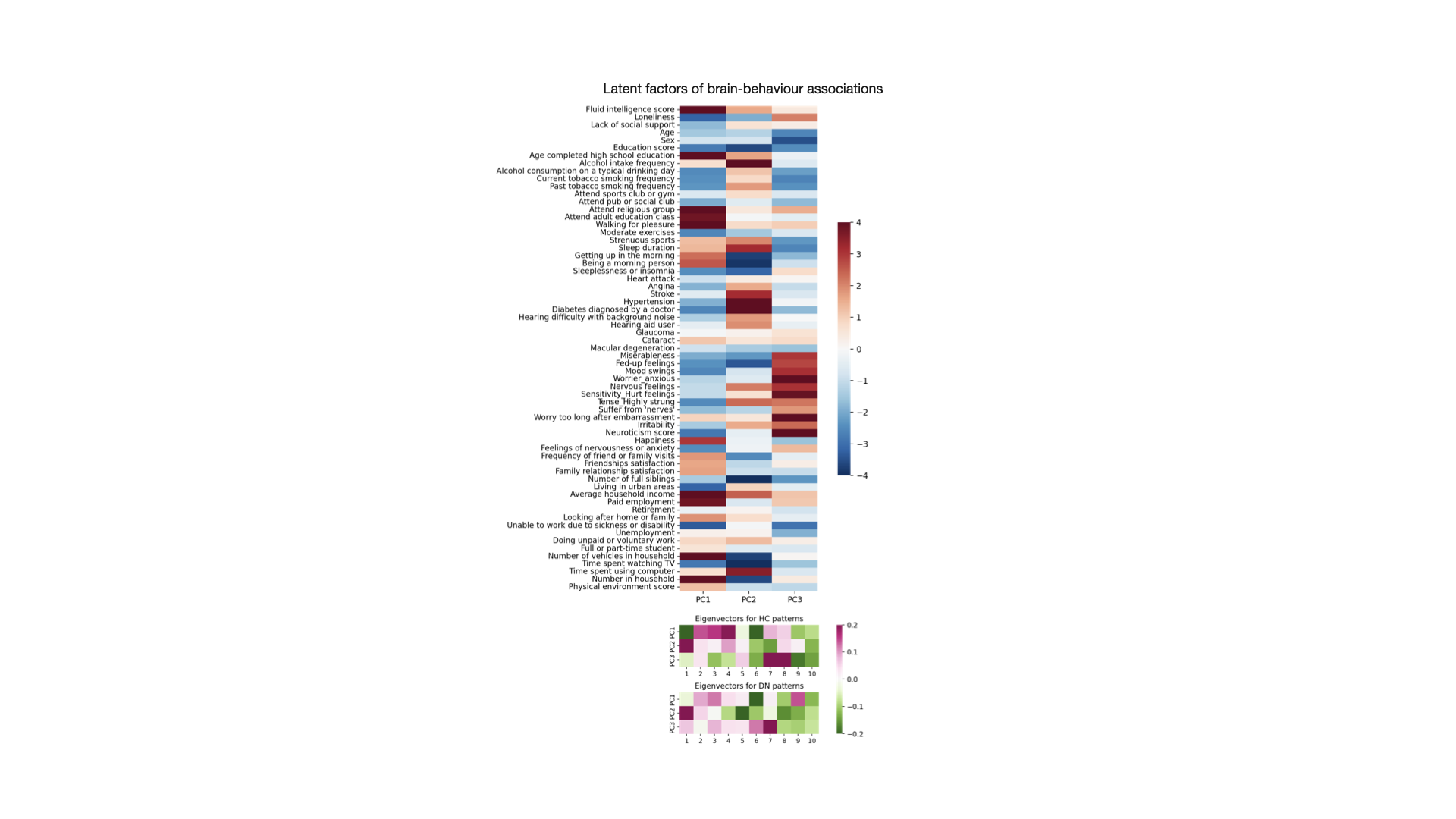

Supplement: S13 Fig — We conducted an exploratory PCA to disentangle latent factor of brain–behaviour association in our UK Biobank sample. We first computed the Pearson’s correlations between the 25 pairs of co-variation patterns from the HC and DN sides and the 63 preselected ADRD risk factors. We then ran singular value decomposition on the risk by canonical variates matrix (X63 × 50) and retained the 3 first PCs that explained approximately 13.8%, approximately 9.6%, and approximately 8.2% of the total variance in the data, respectively. The upper plot displays the projections of the Pearson’s correlations onto each of the 3 main axes of brain–behaviour associations. The lower plot displays the eigenvectors for the top 10 HC and DN co-variation patterns. The first axis of brain–behaviour associations emphasises phenotypes from the social cluster previously identified on the clustering analysis of risk-anatomy links (Fig 4), e.g., attending religious group, attending adult education classes, and number of people in household. The second axis rather accented health-related phenotypes and lifestyle factors. Lastly, the third axis of brain–behaviour associations separated neuroticism-related items (being worried/anxious, being easily hurt, and worrying too long after embarrassment) from the rest of the risk factors. Data underlying this figure can be found at https://github.com/dblabs-mcgill-mila/HCDMNCOV_AD/blob/master/PCA (DOI: 10.5281/zenodo.7126809). ADRD, Alzheimer’s disease and related dementia; DN, default network; HC, hippocampus; PCA, principal component analysis. (TIFF) [file pbio.3001863.s013.tiff]

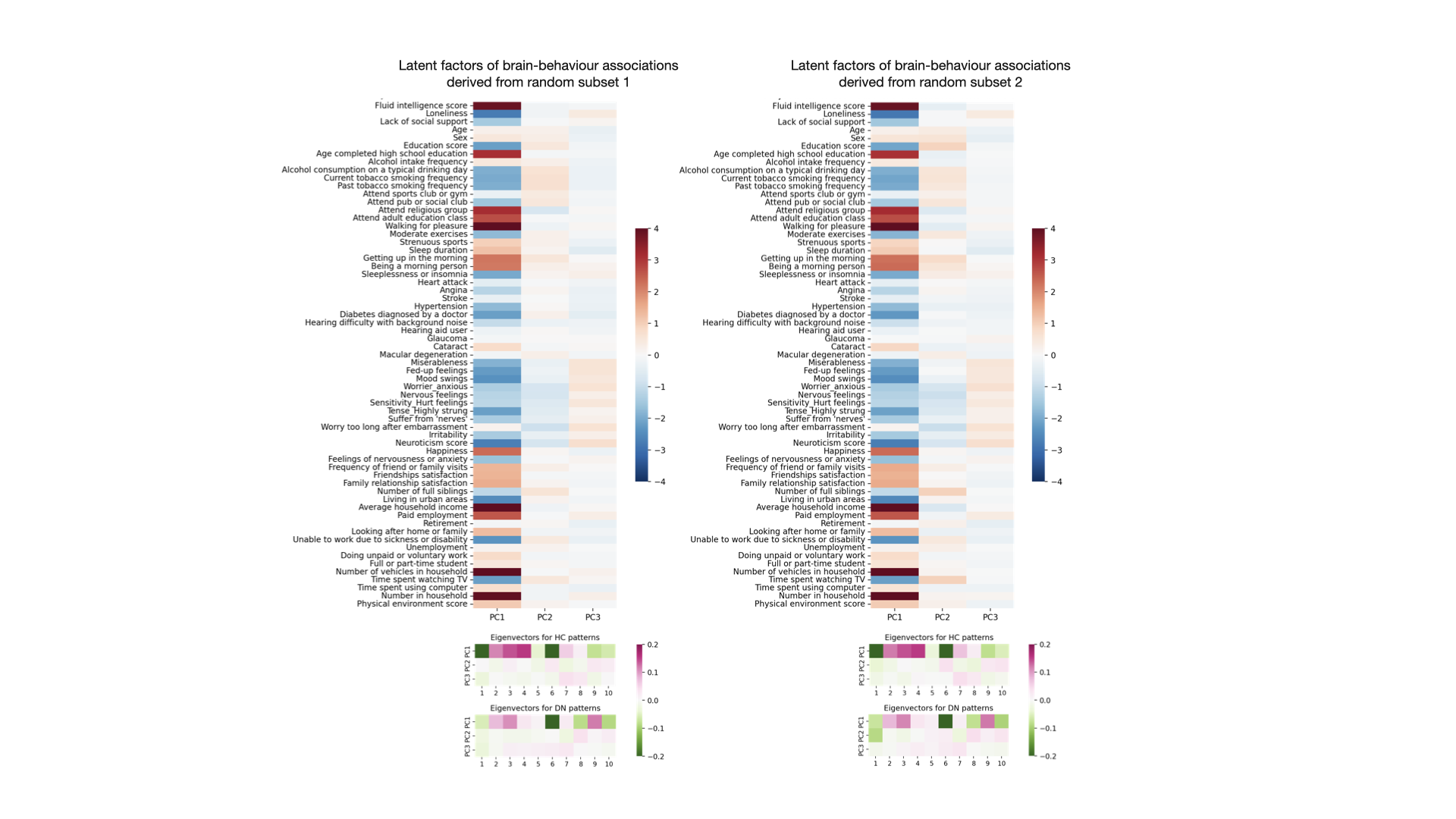

Supplement: S14 Fig — We assessed the robustness of the derived brain–behaviour association axes by performing a split-half reliability assessment of our principal component solution across 1,000 bootstrap iterations. At each iteration, we drew 37,291 participants with replacements to simulate random participant samples that we could have pulled from the same population. We then derived 2 random subsets of equal size (N = 18,645) from the original sample. For each subset, we re-computed the Pearson’s correlation between all possible combinations of the 50 canonical variates and 63 target indicators. We then estimated 2 PCA models in parallel, one for each random half subset, on the z-scored correlation coefficients matrices. We show the average projections of the Pearson’s correlation coefficients on the 3 first axes of brain–behaviour associations. We found that the projections on component 1 were robust and consistent across subsets. The projections on the first axis of brain–behaviour associations accurately depicted those of the original PCA solution, with the same set of social phenotypes (e.g., attending religious group, attending adult education classes, and the number of people in the household) and socioeconomic determinants (e.g., age completed high school education, average household income, and the number of vehicles in the household) emphasised. Data underlying this figure can be found at https://github.com/dblabs-mcgill-mila/HCDMNCOV_AD/blob/master/PCA (DOI: 10.5281/zenodo.7126809). (TIFF) [file pbio.3001863.s014.tiff]

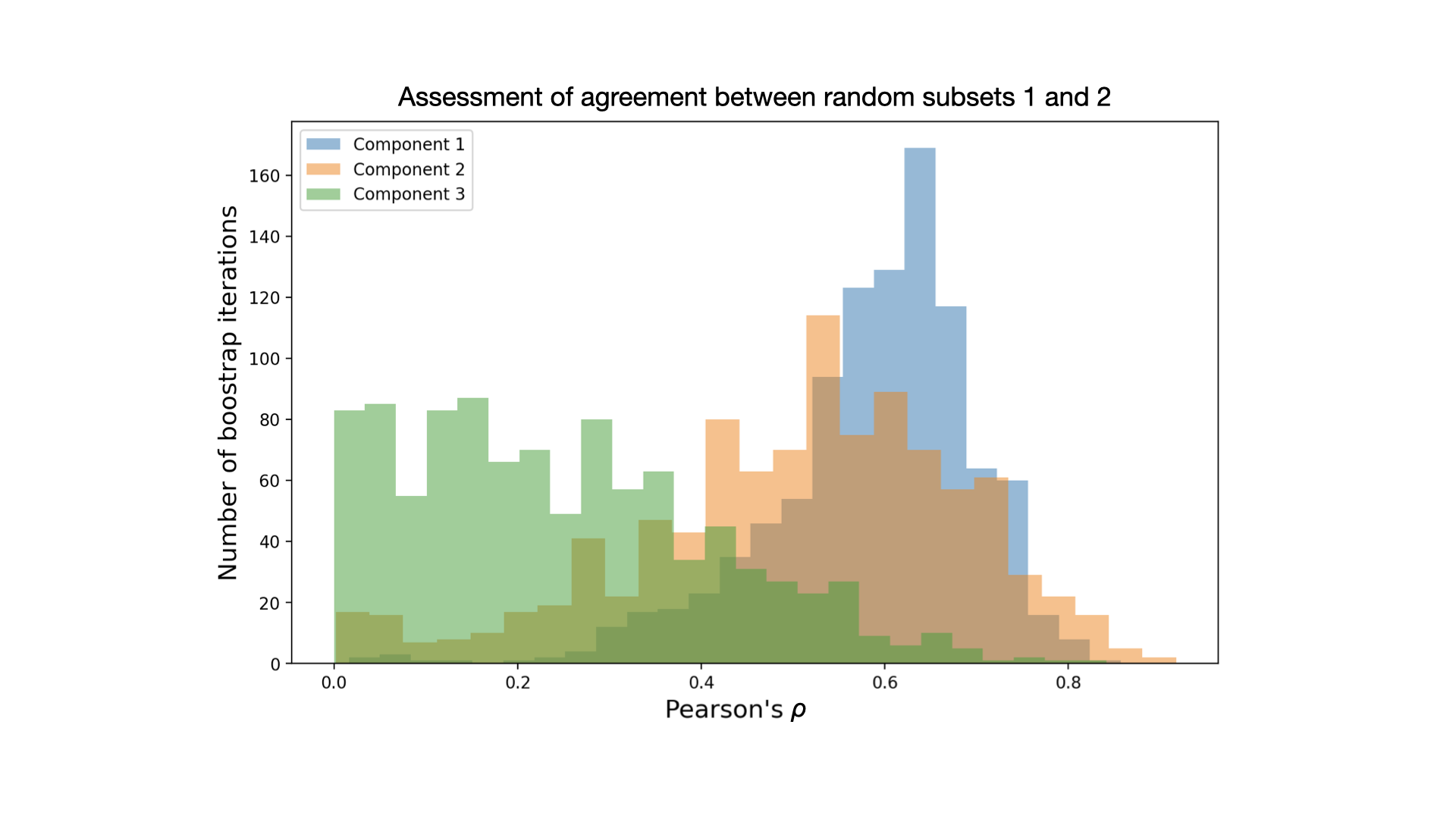

Supplement: S15 Fig — We computed the Pearson’s correlation between the weights of the 3 first principal components for random subsets 1 and 2 across 1,000 bootstrap iterations. The weights of the first 2 components were robust, as reflected by a substantial degree of agreement between both subsets on components 1 (mean Pearson’s rho: 0.59, 90% CI: [0.38, 0.74]) and 2 (mean Pearson’s rho: 0.51, 90% CI: [0.15, 0.77]). In contrast, we showed volatility in the weights associated with component 3, as reflected by a wider and right-skewed distribution (mean Pearson’s rho: 0.25, 90% CI: [0.02, 0.56]). Data underlying this figure can be found at https://github.com/dblabs-mcgill-mila/HCDMNCOV_AD/blob/master/PCA (DOI: 10.5281/zenodo.7126809). (TIFF) [file pbio.3001863.s015.tiff]

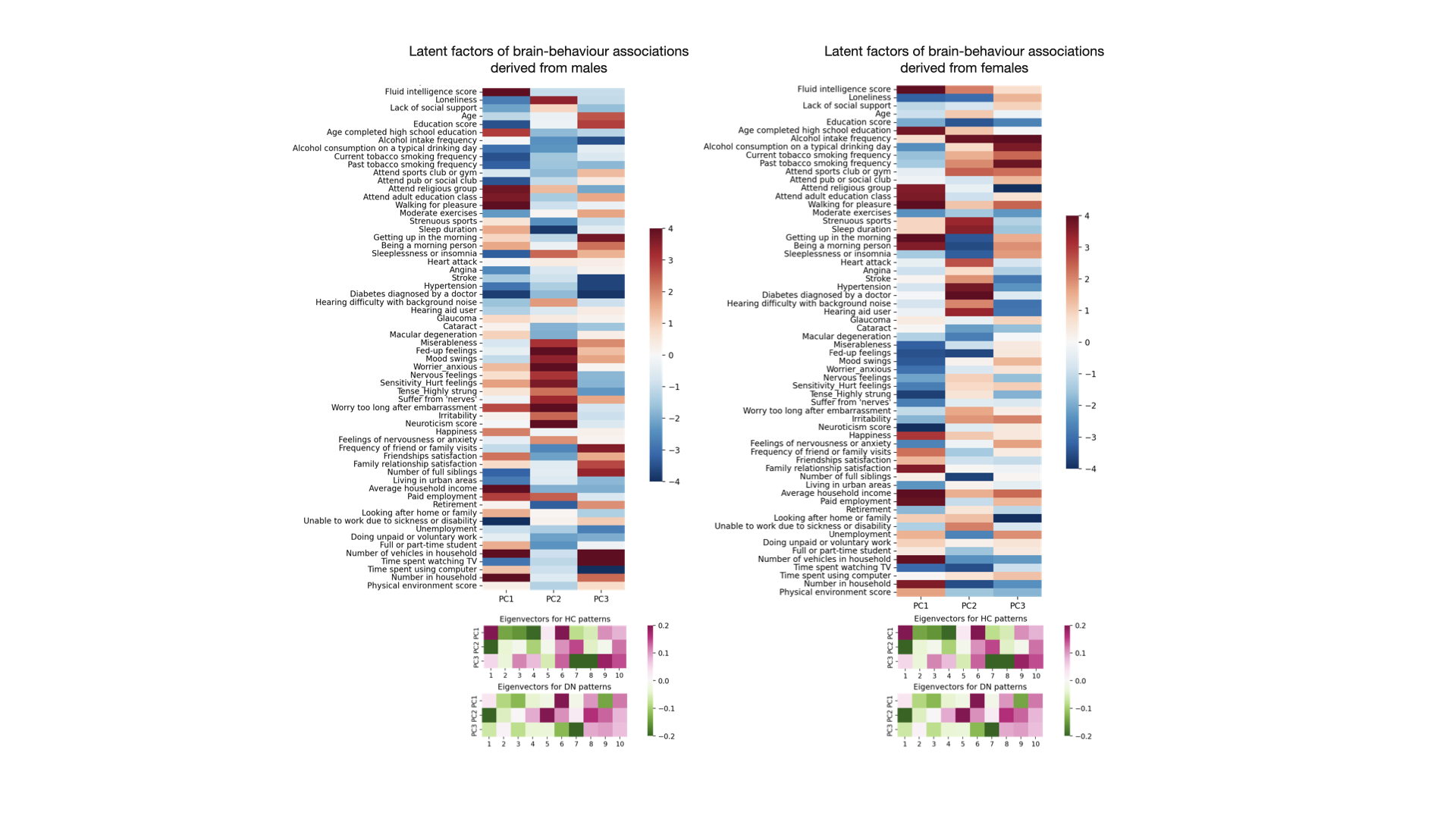

Supplement: S16 Fig — We repeated the PCA in males (left; N = 17,561) and females right; N = 19,730) separately. In each sex, we first computed the Pearson’s correlations between the 25 pairs of co-variation patterns from the HC and DN sides and the 63 preselected ADRD risk factors. We then ran singular value decomposition on the risk by canonical variates matrix (X63 × 50) and retained the 3 first PCs. The PCs obtained from males had explained variance of approximately 14.6%, approximately 11.9%, and approximately 9.6%, respectively. The PCs obtained from females had explained variance of approximately 14.6%, approximately 11.9%, and approximately 7.4%, respectively. The upper plots display the projections of the Pearson’s correlations onto each of the 3 axes of brain–behaviour associations for the 2 sexes. The lower plots display the eigenvectors for the top 10 HC and DN co-variation patterns. The projections of the Pearson’s correlations onto the 2 first axes of brain–behaviour association were roughly the same in males and females. In contrast, neuroticism-related items were only emphasised on the third axis of brain–behaviour association in males. We thus supplemented our population analysis by showing that the relationship between neuroticism and patterns of HC-DN co-variation was mainly male specific. Data underlying this figure can be found at https://github.com/dblabs-mcgill-mila/HCDMNCOV_AD/blob/master/PCA (DOI: 10.5281/zenodo.7126809). ADRD, Alzheimer’s disease and related dementia; DN, default network; HC, hippocampus; PCA, principal component analysis. (TIFF) [file pbio.3001863.s016.tiff]

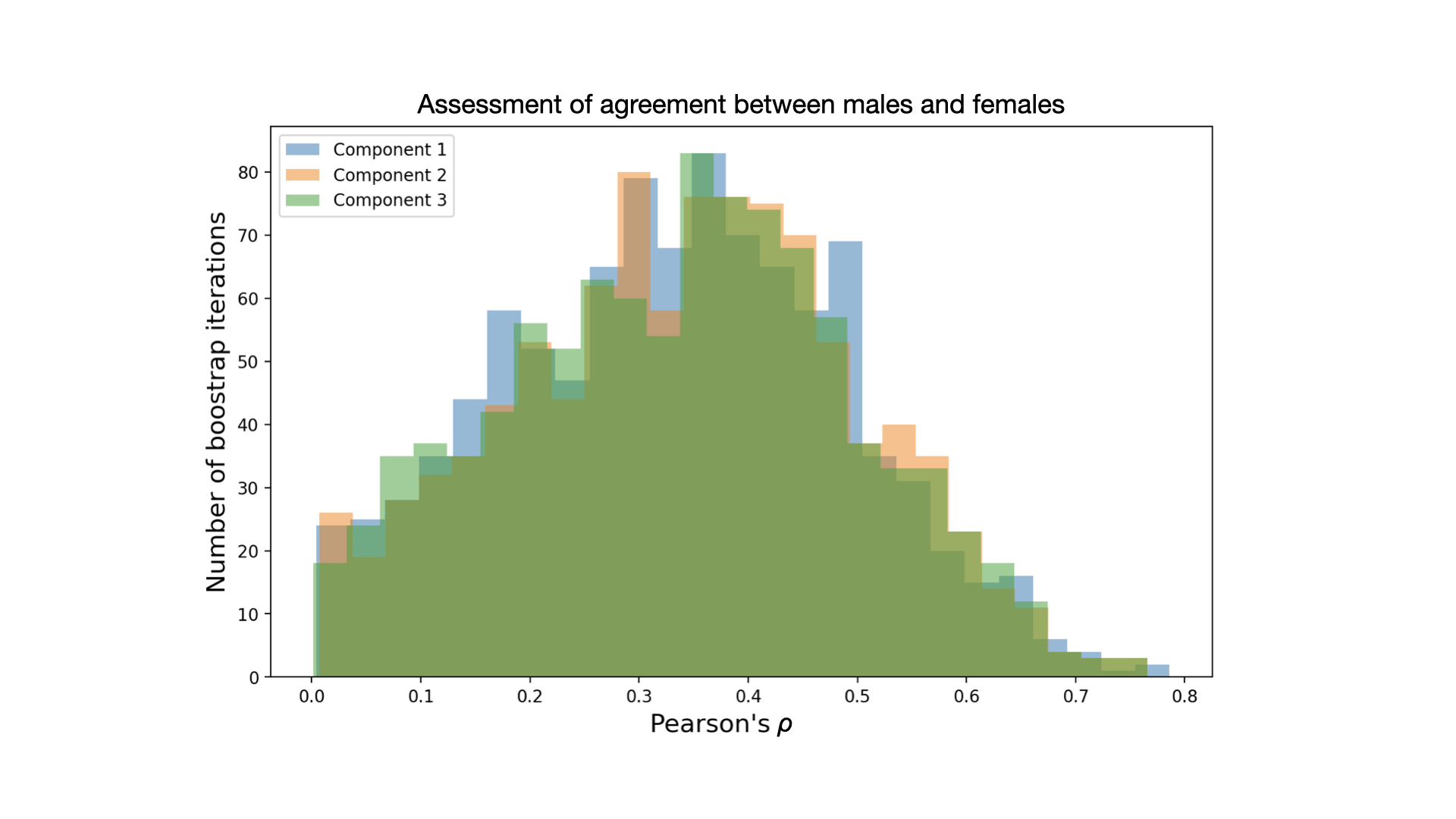

Supplement: S17 Fig — We computed the Pearson’s correlation between the weights of the first 3 principal components for the sex-specific PCA solutions across 1,000 bootstrap iterations. We observed a low agreement between the male- and female-derived PCA solutions on all 3 components, as reflected by the widespread of the distributions and small average values. Data underlying this figure can be found at https://github.com/dblabs-mcgill-mila/HCDMNCOV_AD/blob/master/PCA (DOI: 10.5281/zenodo.7126809). (TIFF) [file pbio.3001863.s017.tiff]

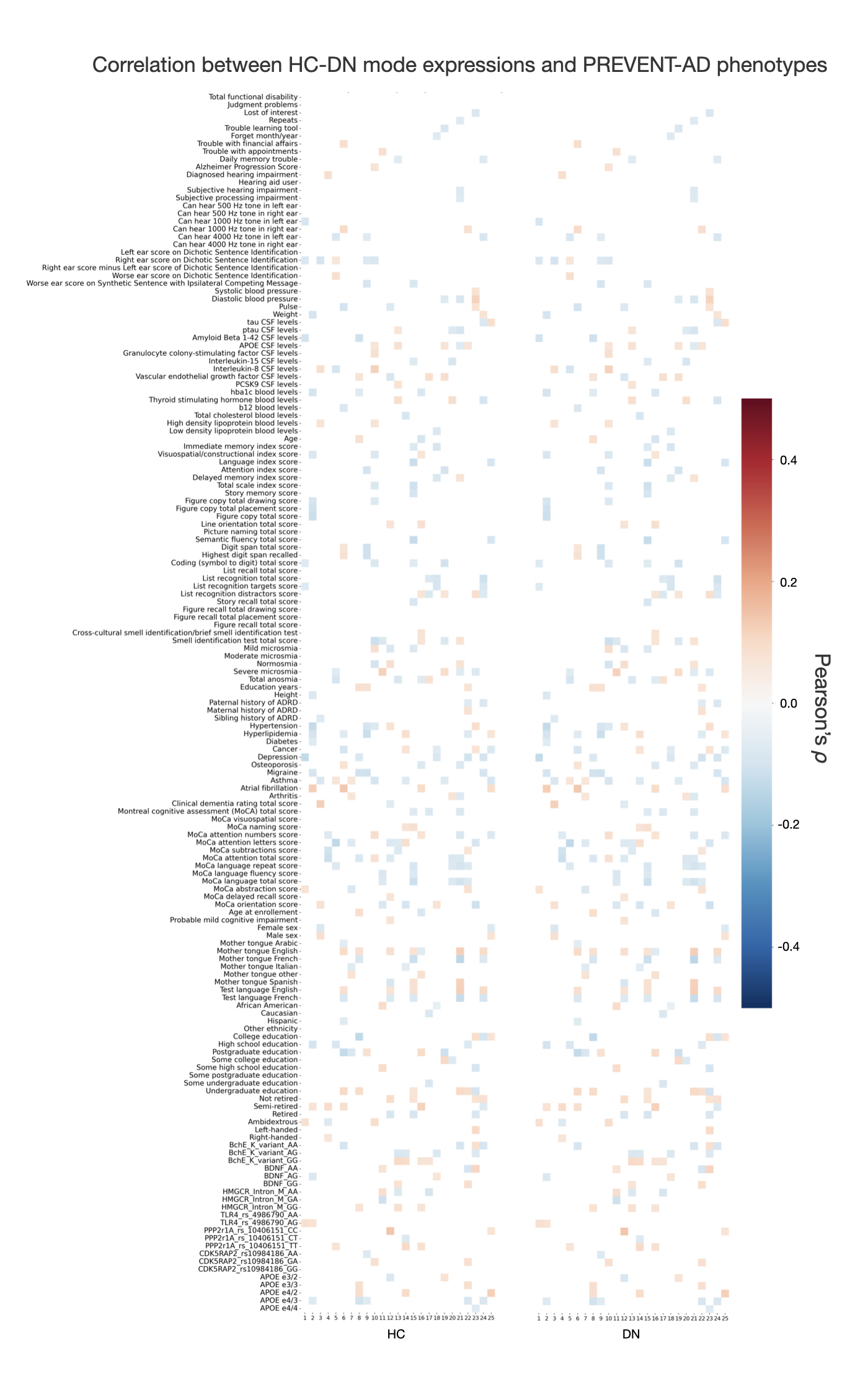

Supplement: S18 Fig — We externally validated our UKB-derived population signatures of HC-DN co-variation by investigating their mapping to ADRD-related risk factors in an unseen, independent participant sample. We tracked subject-specific expressions of the 25 modes of HC-DN co-variation in PREVENT-AD participants to a collection of 157 widely established indicators of ADRD progression. We computed the Pearson’s correlation between the HC and DN pattern expressions and the PREVENT-AD phenotypes for each mode. Only the Pearson’s correlation coefficients that were statistically different from their respective null distributions 95% of the time are present. We replicated several phenotypic associations highlighted in the UKB, such as with mode 1 and depression, mode 2 and verbal-numerical reasoning, and mode 6 and vascular integrity. We also showed that our modes of HC-DN co-variation track meaningful aspects of ADRD progression up to the 25th and last signature, for which we found associations with tau CSF levels on the HC side and cardiovascular factors (e.g., systolic blood pressure, pulse, and APOE ε4/4 genotype) on the DN side. We thus showed that HC-DN signatures robustly link to different aspects of ADRD risk in a completely independent cohort from the one in which the co-variation patterns have originally been derived. Data underlying this figure can be found at https://github.com/dblabs-mcgill-mila/HCDMNCOV_AD/blob/master/external_validation (DOI: 10.5281/zenodo.7126809). ADRD, Alzheimer’s disease and related dementia; DN, default network; HC, hippocampus. (TIFF) [file pbio.3001863.s018.tiff]
